# Efficacy and Safety of Automated Insulin Delivery in People With Type 2 Diabetes: A Systematic Review and Meta‐Analysis

**DOI:** 10.1111/dom.70708

**Published:** 2026-03-27

**Authors:** Sergio Di Molfetta, Ludovico Di Gioia, Irene Caruso, Mariangela Caporusso, Sebastio Perrini, Annalisa Natalicchio, Luigi Laviola, Francesco Giorgino

**Affiliations:** ^1^ Endocrinology Unit University Hospital “Consorziale Policlinico” of Bari Bari Italy; ^2^ Endocrinology Unit Ecclesiastical Entity Regional General Hospital “F. Miulli” Acquaviva delle Fonti Italy; ^3^ Section of Endocrinology, Department of Medicine and Surgery Libera Università Mediterranea (LUM) University Bari Italy; ^4^ Section of Internal Medicine, Endocrinology, Andrology and Metabolic Diseases, Department of Precision and Regenerative Medicine and Ionian Area University of Bari Aldo Moro Bari Italy

**Keywords:** automated insulin delivery, insulin therapy, meta‐analysis, time in range, type 2 diabetes

## Abstract

**Aims:**

Studies evaluating automated insulin delivery (AID) in type 2 diabetes are limited in number and often conducted in small cohorts. We aimed to summarize efficacy and safety data through a systematic review and meta‐analysis.

**Materials and Methods:**

We searched MEDLINE, PubMed, Web of Science, and CENTRAL databases and performed hand searching until July 1, 2025. We included randomized controlled trials enrolling individuals with type 2 diabetes, evaluating AID against other glucose‐lowering treatments, and reporting 24‐h time spent in the 70–180 mg/dL glucose range (TIR 70–180 mg/dL) as an outcome. Risk of bias was assessed through the RoB2 tool.

**Results:**

A total of nine studies, accounting for 714 adults with type 2 diabetes, were included. AID was associated with improved TIR 70–180 mg/dL (mean difference [MD] 18.43%, 95% confidence interval [CI]: 12.40 to 24.46; low certainty), time above range (TAR; low certainty), TAR > 250 mg/dL, mean glucose, standard deviation (moderate certainty), and HbA1c (moderate certainty) compared to controls, without differences in time below range (TBR; moderate certainty), TBR < 54 mg/dL, and coefficient of variation. Despite similar total daily insulin doses, AID led to a modest gain in body weight (MD 1.58 kg; 95% CI: 0.75 to 2.40) compared to controls. Severe adverse events, including severe hypoglycemia, were rare. No episodes of diabetic ketoacidosis were reported.

**Conclusions:**

In individuals with type 2 diabetes, AID is associated with short‐term improvements in glycemic control, although the certainty of evidence is low to moderate.

**Trail Registration:** PROSPERO 2025 CRD420251083874.

## 1 Introduction

Despite the recent development of efficacious innovative drugs, including sodium‐glucose cotransporter‐2 (SGLT‐2) inhibitors and incretin‐based agents, insulin therapy remains a cornerstone of treatment in 30% to 40% of people with type 2 diabetes [1, 2, 3].

More importantly, a substantial proportion of patients fail to achieve adequate glycemic control irrespective of the adopted therapeutic approach. A recent systematic review reported that the prevalence of inadequate glycemic control among individuals with type 2 diabetes ranged from 45.2% to 93%, with only 23.75% (95% confidence interval [CI]: 21.47 to 26.18) of insulin‐treated patients achieving an HbA1c level below 7% [4].

In people with type 1 diabetes, automated insulin delivery (AID) systems—consisting of a portable insulin pump, a disposable sensor for continuous glucose monitoring (CGM), and a control algorithm continuously adjusting insulin delivery in response to changing glucose levels—have been evaluated in a large number of studies showing unprecedented improvements of HbA1c, time spent in the 70–180 mg/dL glucose range (TIR 70–180 mg/dL), time spent in hyperglycemia, coefficient of variation of glucose (CV), and hypoglycemia risk as compared with other insulin‐based therapies [5, 6]. Notably, beneficial effects on glycemic outcomes have been reported for both hybrid and fully (i.e., without user‐led announcements of meals and/or physical activity) closed‐loop system configurations [6].

Different from type 1 diabetes, studies evaluating AID in type 2 diabetes are limited in number and often conducted in small cohorts. However, among commercially available systems, MiniMed 780G (European Union and United States), Omnipod 5 (United States), and Tandem t:slim X2 with Control‐IQ+ technology (United States) are licensed for type 2 diabetes. The aim of this meta‐analysis of randomized controlled trials (RCTs) is to summarize efficacy and safety data for use of AID systems in people with type 2 diabetes.

## 2 Methods

The study protocol was registered prior to conduct (PROSPERO 2025 CRD420251083874).

### 2.1 Data Sources

We searched MEDLINE (via Ovid), PubMed, Web of Science, and CENTRAL from inception to July 1, 2025 (Appendix S1) and performed hand‐searching to find all relevant studies outside the databases.

### 2.2 Ethics Statement

Analyses were performed on data extracted from published papers. Patient consent for publication was not required.

### 2.3 Study Selection

We included RCTs enrolling individuals with type 2 diabetes, evaluating AID systems through either hybrid closed‐loop (HCL) or fully closed‐loop (FCL) configurations, compared with other glucose‐lowering treatments, and reporting 24‐h TIR 70–180 mg/dL as an outcome. Trials conducted in pregnant women or evaluating AID during experimentally‐induced stress challenges (physical exercise, gastronomic meals, etc.) were excluded, as insulin therapy in these subgroups and/or situations may not reflect the usual practice. Trials conducted in mixed cohorts of people with diabetes not reporting outcomes separately for individuals with type 2 diabetes were also excluded.

The primary outcome of the meta‐analysis was 24‐h TIR 70–180 mg/dL (mean difference [MD] with 95% CI). Secondary outcomes were 24‐h time spent in the 70–140 mg/dL glucose range (time in tight range, TITR), 24‐h time below 70 mg/dL (time below range, TBR), 24‐h time below 54 mg/dL (TBR < 54 mg/dL), 24‐h time above 180 mg/dL (time above range, TAR), 24‐h time above 250 mg/dL (TAR > 250 mg/dL), 24‐h mean sensor glucose, standard deviation (SD) of glucose, CV, HbA1c, total daily insulin dose (TDD), change in body weight (BW), occurrence of severe hypoglycemia (SH), occurrence of diabetic ketoacidosis (DKA), and patients' reported outcome measures (PROMs) including treatment satisfaction and quality of life. Continuous and binary outcomes were reported as MD and odds ratio (OR), respectively, with their 95% CI and 95% prediction interval (PI). All causes of death, occurrence of major adverse cardiovascular events, infected ulcers, lower limb gangrene or lower limb amputation, development/worsening of diabetic retinopathy or nephropathy, and need for renal replacement therapy were also collected.

### 2.4 Data Extraction

Three reviewers (IC, LDG, SDM) independently evaluated article titles and abstracts based on inclusion and exclusion criteria. Disagreements were settled by discussion. The following information was collected from the included studies: study characteristics (design, outpatient or inpatient setting, duration, year of publication, and sample size), participants' characteristics (age, sex, body mass index [BMI], HbA1c at baseline [%], diabetes duration, and insulin requirements), type of AID system under evaluation, comparator(s), TIR 70–180 mg/dL (%), TITR (%), TBR (%), TBR < 54 mg/dL (%), mean sensor glucose (mg/dL), TAR (%), TAR > 250 mg/dL (%), CV (%), HbA1c (%, mmol/mol), proportion of patients having SH or DKA. Study investigators were contacted in case of missing data. If the mean was missing, it was calculated by dividing the sum of the median, first quartile, and third quartile by 3 [7]. If the SD was missing, it was calculated by multiplying the standard error (SE) by the square root of the sample size [7]. When the SD was neither available nor calculable, it was imputed based on the higher value within each group [8]. Disagreements in data extraction were settled by debate.

### 2.5 Risk of Bias Assessment

Risk of bias was assessed independently by two reviewers (IC, LDG) through the Cochrane Collaboration's tool (RoB 2, version 22 August 2019; RoB 2 crossover, version 18 March 2021) evaluating the following domains: randomization process; bias arising from period and carryover effects; deviations from intended intervention; missing outcome data; measurement of the outcome; selection of the reported result; overall bias. Each domain was deemed to be at low, with some concerns or high risk of bias. Any differences in assessment were resolved by discussion.

### 2.6 Statistical Analysis

The systematic review and the meta‐analysis were performed in line with recommendations from the Cochrane Collaboration and the Preferred Reporting Items for Systematic Reviews and Meta‐Analysis (PRISMA) statement guidelines. In the descriptive analyses, continuous variables were summarized as mean ± SD or as median with interquartile range (IQR) in case of non‐normal distribution, and categorical variables as absolute counts and/or percentages. In the meta‐analysis, continuous outcomes were reported as means with 95% CIs, and binary outcomes as proportions with the corresponding 95% CIs. Ninety‐five percent PIs were also computed to show the range within which the relative treatment effect of a future study is expected to fall, accounting for between‐study heterogeneity. In addition, heterogeneity was assessed using Cochran's *Q* test and quantified by the *I* ^2^ statistic: *p* values < 0.05 and *I* ^2^ > 25% were considered significant for heterogeneity. Given the substantial heterogeneity observed across studies, all meta‐analyses were conducted using random‐effects models [9]. The presence of publication bias was evaluated using funnel plots and Egger's tests, when feasible. Certainty of evidence was assessed according to the Grading of Recommendations Assessment, Development and Evaluation (GRADE) methodology, through GRADEpro. For all outcomes, we performed sensitivity analyses by restricting the dataset to trials with an intervention duration of ≥ 2 weeks, to those at low risk of bias, to those evaluating AID systems compared with other types of insulin therapy, and excluding trials for which SDs were imputed. Prespecified subgroup analyses were performed by type of system (HCL vs. FCL) and setting of care (outpatient vs. inpatient). Meta‐regression analyses were also performed to explore the association between MD in TIR 70–180 mg/dL and variables such as participants' age, BMI, baseline HbA1c, diabetes duration and intervention duration. For each of these variables, regression plots were generated, displaying predicted TIR 70–180 mg/dL estimates at representative values of the covariates. The Instrument for assessing the Credibility of Effect Modification Analyses (ICEMAN) tool was used to assess credibility of subgroup effects for interaction *p* values < 0.1. All analyses were performed using RStudio and R package meta [10].

## 3 Results

**FIGURE 1:**
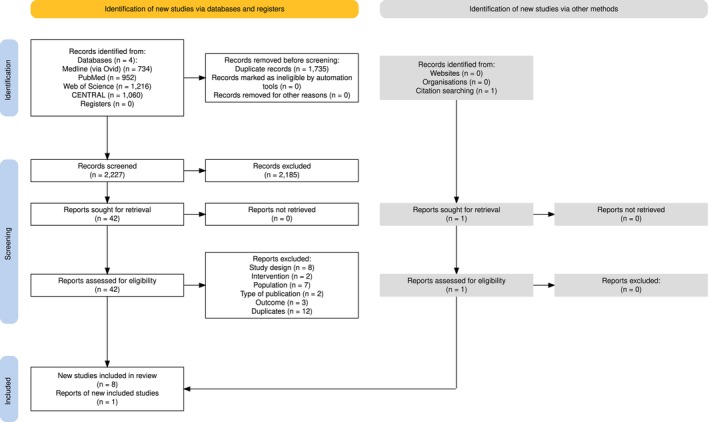
Preferred Reporting Items for Systematic reviews and Meta‐Analyses (PRISMA) flow diagram of study search strategy.

A total of nine studies [11, 12, 13, 14, 15, 16, 17, 18, 19] (714 subjects) were included in the meta‐analysis. After an initial screening based on title and abstract, 42 full‐text articles were excluded, either because they were duplicates (*n* = 12) or because their study design (*n* = 8), intervention (*n* = 2), population (*n* = 7), type of publication (*n* = 2), or outcomes (*n* = 3) were not relevant to our research question. Specifically, studies by Bally L et al. [20] and Thabit et al. [21] were excluded because they only reported Time in Range 100–180 mg/dL, whereas a study by Boughton CK et al. [22] was excluded because approximately 50% of participants had non‐type 2 or non‐purely type 2 diabetes (i.e., pancreatogenous, steroid‐induced, and feeding‐induced diabetes) and did not report outcomes separately for type 2 diabetes. Differently from this latter study, the study by Herzig et al. was included because the proportion of participants with non‐type 2 diabetes was apparently marginal (around 9%) [20]. Details of the screening process and the key characteristics of the included studies are provided in Figure 1 and Appendix S2, respectively. All studies included, except one [11], enrolled insulin‐treated subjects. Prior CGM use was reported in four studies [16, 17, 18, 19], with the proportion of participants already using CGM ranging from 12% to 100%. Participant age ranged from 57.2 ± 14.4 [11] to 69.5 ± 8.6 years [17], diabetes duration from 7.6 ± 6.1 [11] to 24 ± 9 years [18], and baseline HbA1c from 7.2% ± 1.3% (55 ± 14 mmol/mol) [14] to 9.1% ± 1.1% (76 ± 12 mmol/mol) [17]. Ethnicity was reported in three studies [11, 16, 19], which indicated a predominance of White participants, whereas socioeconomic status was reported in a single study [19] and showed a balanced distribution across the population. Five studies (*n* = 307) evaluated FCL systems, while four (*n* = 407) evaluated HCL systems. Time spent in automode was reported in 7 out of 9 studies and exceeded 90% in all of them, except for the study by Reznik Y et al. (median: 85.5%, *n* = 11). In participants treated with HCL systems, fixed prandial doses or semiquantitative meal estimation were the preferred strategies for meal announcement rather than carbohydrate counting. Control treatments comprised oral anti‐diabetic agents such as metformin, sulfonylureas and dipeptidyl peptidase‐4 inhibitors in one study [11], continuous subcutaneous insulin infusion (CSII) in another [18], and insulin injections in the rest. Two studies (*n* = 180) involved hospitalized patients, whereas seven studies (*n* = 534) were conducted in outpatient populations. Of these, two (Kumareswaran K et al. and Taleb N et al.) were carried out in clinic settings, and the remaining five under free‐living conditions.

**FIGURE 2:**
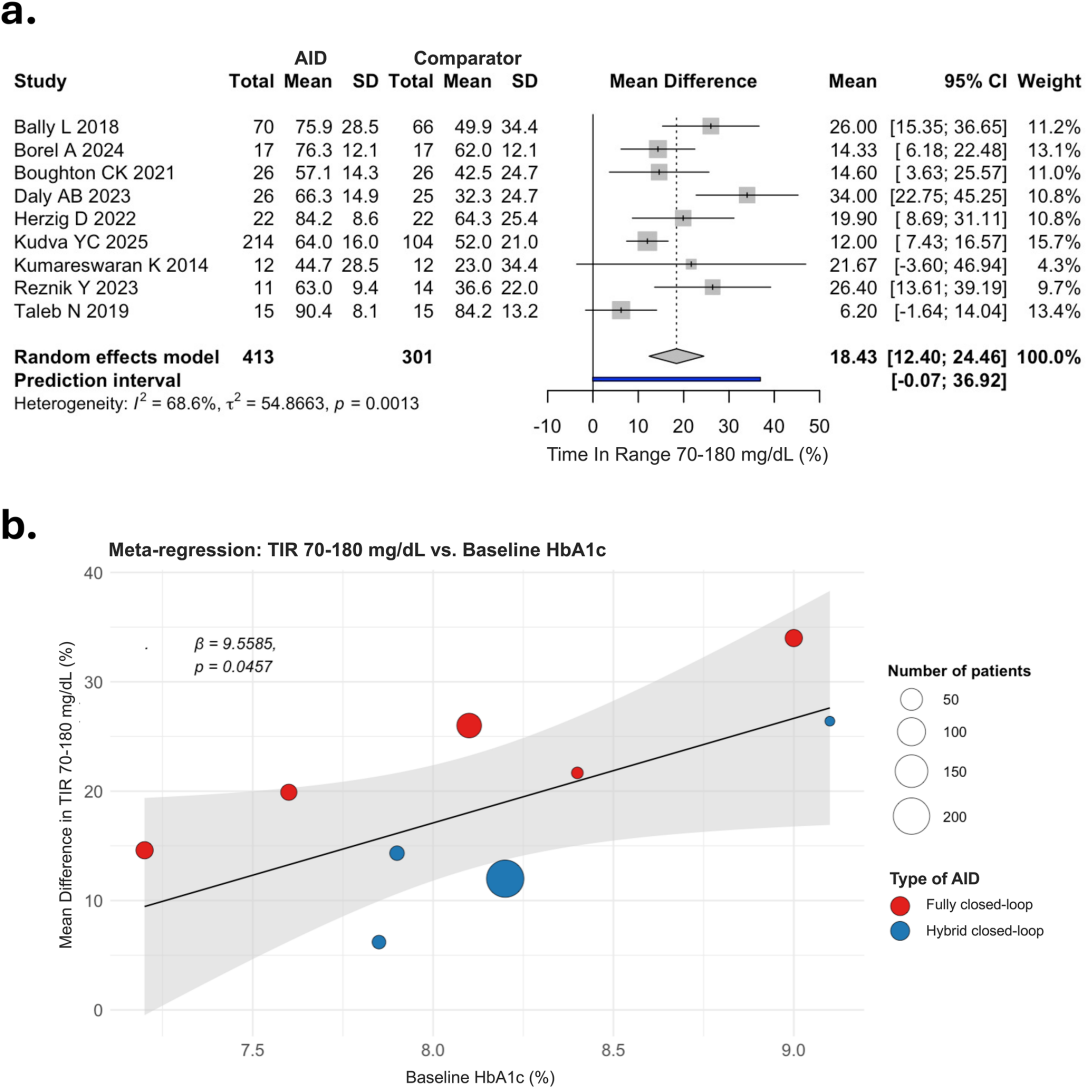
Forest plot of mean difference (MD) in Time In Range 70–180 mg/dL (TIR 70–180 mg/dL) between automated insulin delivery (AID) and comparator groups (a). Meta‐regression of MD in TIR 70–180 mg/dL vs. baseline HbA1c; bubble size is proportional to sample size (red, fully closed‐loop; blue, hybrid closed‐loop) (b). CI, confidence interval; SD, standard deviation.

Use of AID systems was associated with improved TIR 70–180 mg/dL (MD 18.43%, 95% CI: 12.40 to 24.46) as compared with controls (low certainty) (Figure 2a). At meta‐regression analyses, higher baseline HbA1c values were associated with a greater improvement in MD for TIR 70–180 mg/dL (*β* = 9.56, *p* = 0.046), as shown in Figure 2b. The credibility of the potential effect modification was deemed moderate, with uncertainty primarily arising from the use of study‐level data, the relatively low number of included studies, and a weakly supportive *p* value for interaction. No significant associations were observed for participants' age, BMI, diabetes duration, or duration of intervention.

**FIGURE 3:**
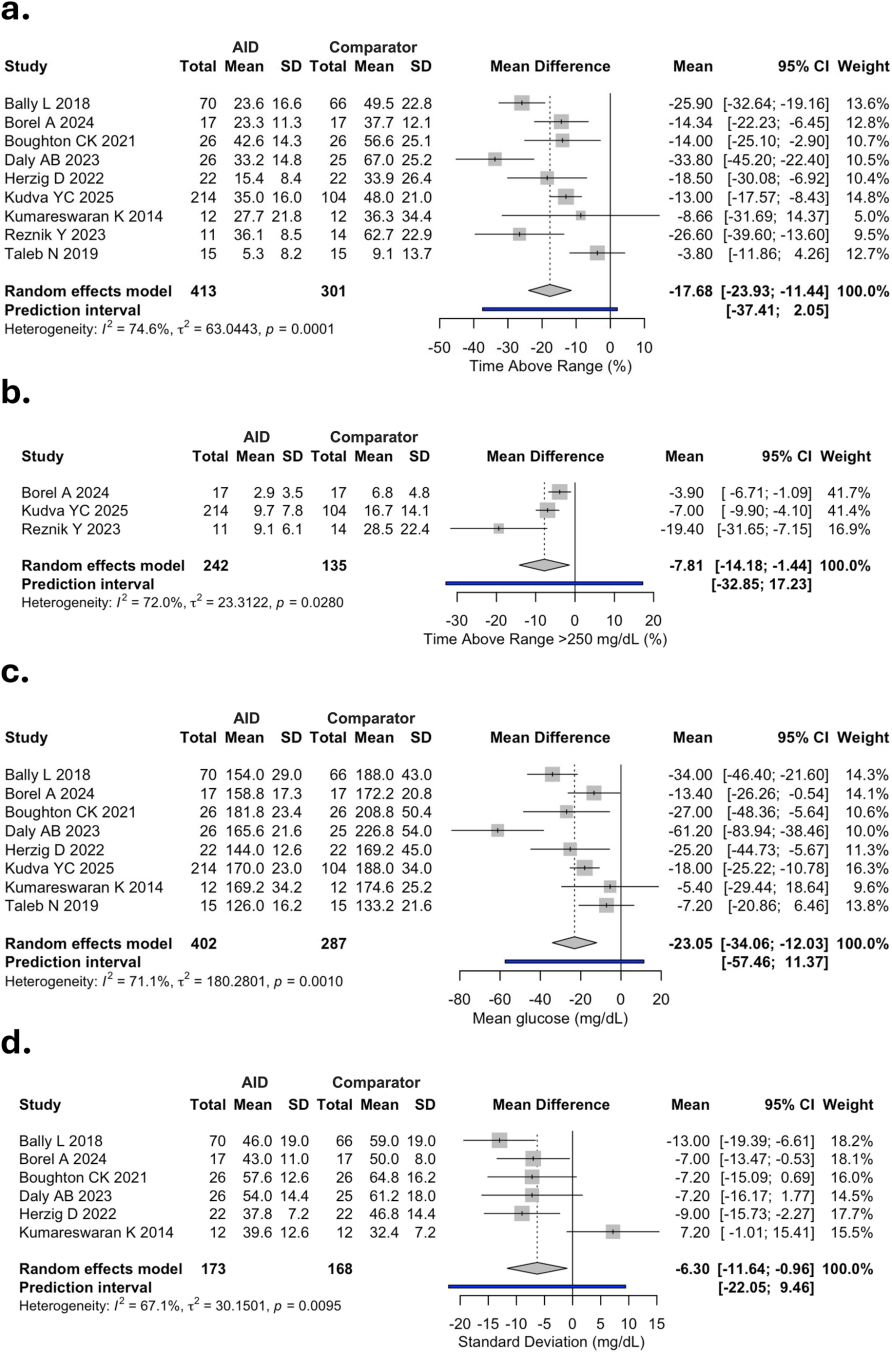
Forest plots of mean difference (MD) in Time Above Range > 180 mg/dL (a), Time Above Range > 250 mg/dL (b), mean glucose (c) and SD (d) between automated insulin delivery (AID) and comparator groups. CI, confidence interval; SD, standard deviation.

TITR was reported only in the study by Taleb et al. [13], showing a mean difference of 9.5% in favour of AID (*p* = 0.05); therefore, a meta‐analysis was not conducted. TAR was reported in nine studies, TAR > 250 mg/dL in three studies (*n* = 377), and mean glucose in eight (*n* = 689), with observed MDs of −17.68% (95% CI: −23.93 to −11.44; low certainty), −7.81% (95% CI: −14.18 to −1.44), and −23.05 mg/dL (95% CI: −34.08 to 12.03), respectively, comparing AID systems with the control group (Figure 3a–c, and Appendix S5).

SD (six studies, *n* = 341) was significantly lower with AID (MD −6.30 mg/dL, 95% CI: −11.64 to −0.96; moderate certainty), whereas CV (seven studies, *n* = 660) did not differ between groups (Figure 3d and Appendix S5). Nonsignificant differences between the two groups were also observed for TBR (nine studies, *n* = 714; moderate certainty), TBR < 54 mg/dL (seven studies. *n* = 660), TDD (eight studies, *n* = 681; low certainty).

**FIGURE 4:**
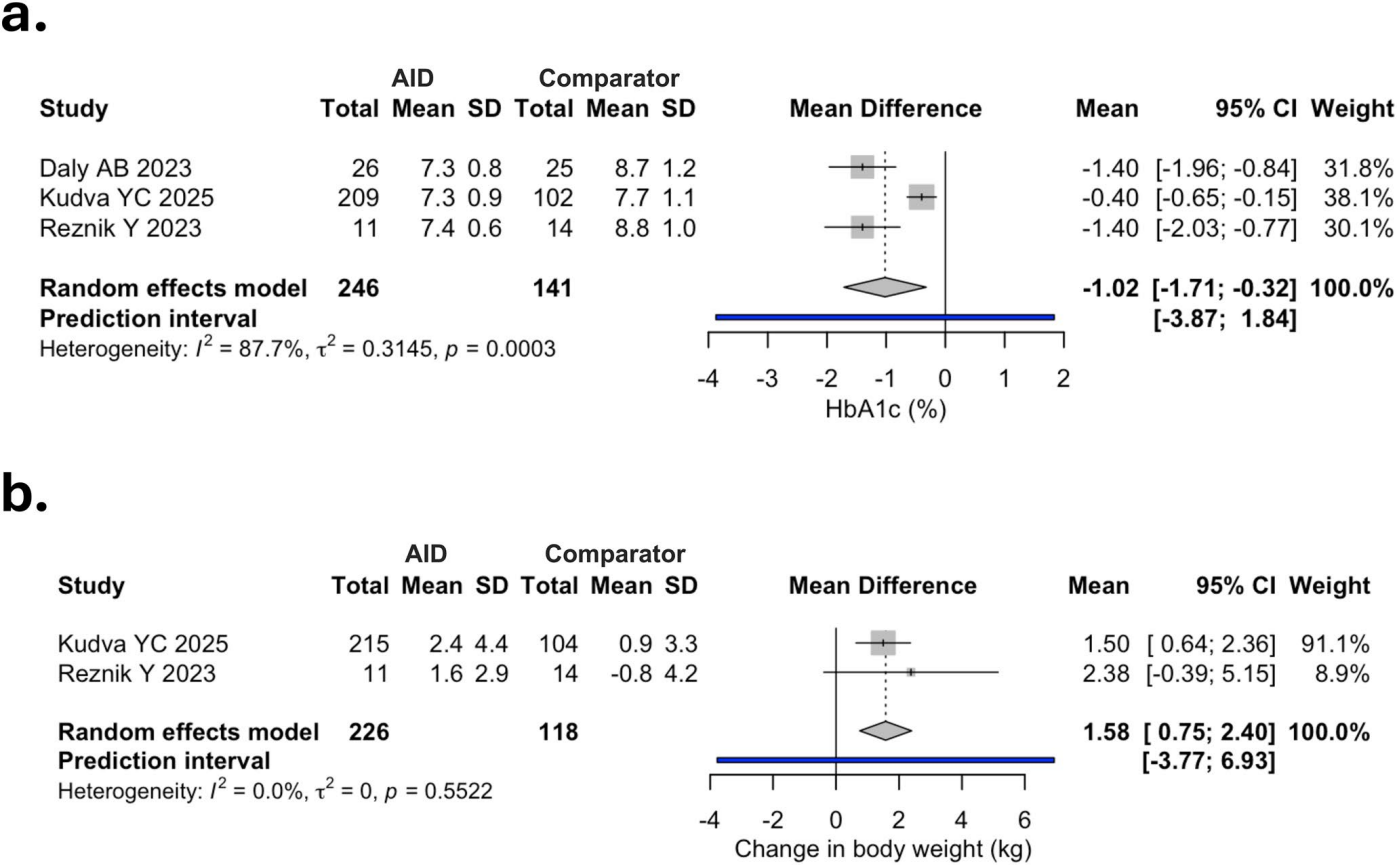
Forest plots of mean difference (MD) in HbA1c (a) and change in body weight (b) between automated insulin delivery (AID) and comparator groups. CI, confidence interval; SD, standard deviation.

A total of three studies (*n* = 387) reported HbA1c outcomes, showing 1.02% reduction (95% CI: −1.71 to −0.32; −12 mmol/mol, 95% CI: −19 to −4; moderate certainty) in favour of AID (Figure 4a). Finally, change in BW was reported in only 2 studies (*n* = 344), showing modest BW gain (MD 1.58 kg, 95% CI: 0.75 to 2.40; high certainty) with AID as compared with controls (Figure 4b).

Subgroup analyses by type of AID system (FCL vs. HCL) and care setting (inpatient vs. outpatient) revealed no significant differences except for TIR 70–180 mg/dL, TBR < 54 mg/dL, and CV, respectively (Appendies S7 and S8).

Indeed, the use of FCL systems was associated with a significantly greater improvement in TIR 70–180 mg/dL (MD 23.45%, 95% CI: 16.09 to 30.83) compared to HCL systems (MD 13.32%, 95% CI: 6.99 to 19.66). Moreover, unlike HCL systems, FCL systems led to a significant, although subtle, reduction in TBR < 54 mg/dL (MD −0.07%, 95% CI: −0.12 to −0.01).

Unlike studies conducted in the outpatient setting, those conducted in the inpatient setting showed that the use of AID systems was associated with a significant reduction in CV (MD −2.5%, 95% CI: −4.6 to −0.4; Appendix S8), although the credibility of this analysis was deemed low mostly due to the use of aggregate data and the small number of included studies.

Sensitivity analyses were largely consistent with the main analysis, although some exceptions warrant attention (Appendix S5.2–S5.5). Notably, greater reductions in mean glucose levels with AID compared to control interventions were observed in trials with an intervention duration of ≥ 2 weeks (MD −28 mg/dL, 95% CI: −40.1 to −15.9) and in those assessed as having a low risk of bias (MD −31.2 mg/dL, 95% CI: −49.2 to −13.1). Additionally, trials comparing AID to other forms of insulin therapy demonstrated a more pronounced reduction in glycemic variability, as measured by the SD of glucose (MD −8.99 mg/dL, 95% CI: −12.17 to −5.81). In contrast, no significant differences in HbA1c levels were observed between AID and comparator therapies in the two trials with low risk of bias. SH occurred in only two individuals across all studies included in the meta‐analysis, both of whom were treated with AID systems. No episodes of DKA were reported. The other severe adverse events are detailed in Appendix S10. In total, ten prespecified events of interest were reported, two of which occurred in the AID groups. Given the low number of events, no meta‐analysis was performed for these outcomes. Of note, two studies [12, 14] enrolled patients with end‐stage renal disease undergoing dialysis.

PROMs were assessed in 6 out of 9 included studies using different questionnaires (Appendix S11), therefore a meta‐analysis of these outcomes was not conducted. AID was generally associated with similar or improved treatment satisfaction, perceived diabetes burden, perceived hypoglycemia burden, and sleep quality compared to control groups, with the exception of a single study [16] reporting increased fear of hypoglycemia with AID. Across different studies [12, 14, 16, 17, 18], a high proportion of participants reported a positive experience with AID and indicated they would recommend this treatment to others with diabetes.

## 4 Discussion

Despite advancements in treatment options, many people with type 2 diabetes struggle to achieve glycemic goals. Our meta‐analysis demonstrates that, in individuals with type 2 diabetes, AID improves glycemic control without increasing hypoglycemia compared to other treatment modalities, particularly other types of insulin therapy.

Moreover, according to the meta‐regression analysis, individuals with higher baseline HbA1c levels derived greater benefit from AID, with an estimated ~10% greater difference in TIR 70–180 mg/dL for each 1% higher baseline HbA1c.

Beneficial effects on glycemic control have been reported in both inpatient (*n* = 180) and outpatient (*n* = 534) populations, with both HCL and FCL configurations. In line with our findings, in a meta‐analysis of seven RCTs on individuals with type 2 diabetes, Amer BE et al. found improved time in the target glucose range (5.6–10 mmol/L, 3.9–10 mmol/L, or 3.9–8 mmol/L, depending on the study) without increased time spent in hypoglycemia with FCL insulin delivery [23]. While we acknowledge that alternative glucose target ranges may be more appropriate in specific clinical contexts (e.g., inpatient settings, perioperative periods) [24, 25], we chose to include only studies reporting TIR 70–180 mg/dL, or where this could be derived from TAR and TBR, in order to ensure greater comparability across the included studies and with existing evidence in the literature. Therefore, TIR 70–180 mg/dL was consistently used for the meta‐analysis and presented in the forest plots.

Interestingly, improved glycemic control was achieved without significant differences in TDD, possibly reflecting a combination of reduced insulin requirements with CSII compared with multiple daily injections (MDI), and increased insulin delivery provided by AID systems in individuals with poor baseline glycemic control. Consistent with this interpretation, CSII has previously been associated with improved glycemic outcomes accompanied by substantial reductions in insulin requirements compared with MDI in type 2 diabetes [26]. Conversely, the only study in our meta‐analysis that compared AID with non‐automated CSII therapy reported a 23% increase in TDD together with a 14.3% increase in TIR with AID [18].

Despite a comparable TDD, a slight increase in BW (1.58 kg, 95% CI: 0.75 to 2.40, 95% PI: −3.77 to 6.93) was observed in participants using AID systems compared to the control group. This finding might be attributed to reduced glycosuria and increased caloric retention associated with improved glycemic control [27]. However, this observation is based on only two trials; given the importance of BW management in type 2 diabetes care, further studies are needed to clarify this aspect, as suggested by the wide PI value.

Severe adverse events, including SH and DKA, were very infrequent in both the AID and control arms, preventing a meta‐analysis with adequate power for these outcomes. Nevertheless, the consistently low event rates observed with AID systems across studies are reassuring and support the overall favorable safety profile of this technology, at least in the short term.

Across all included studies, similar or improved PROMs were reported with AID, with the exception of increased fear of hypoglycemia in one single study [16]. Participants generally reported positive experiences and would recommend AID to others with diabetes. This finding is especially relevant when considering that the above‐mentioned studies enrolled individuals with a mean age ranging from 59.0 to 69.5 years, potentially encompassing participants with reduced technological literacy or age‐related visual and motor impairments. Importantly, evidence from type 1 diabetes cohorts also suggests high usability of AID systems and strong adherence among older adults [28].

To the best of our knowledge, this is the most comprehensive meta‐analysis focused on type 2 diabetes that includes evidence from RCTs evaluating either FCL or HCL systems, including two commercial devices (i.e., Tandem t:slim X2 with Control‐IQ technology and Accu‐Chek Insight with DBLG1), and reporting TIR 70–180 mg/dL.

Another meta‐analysis with similar aims to ours, including six RCTs and nine single‐arm studies, was recently published [29]. However, it inappropriately included one RCT evaluating a standalone insulin pump (Paradigm VEO) against MDI therapy and, conversely, failed to include four relevant RCTs, two conducted in an outpatient setting [11, 13] and two in an inpatient setting [12, 15].

Importantly, among the two commercial AID systems included in our meta‐analysis, only one (i.e., Control‐IQ+) is currently authorized by regulatory agencies for use in people with type 2 diabetes. The two other systems approved for individuals with type 2 diabetes and commercially available have so far been evaluated only in non‐randomized studies. Indeed, in an 8‐week single‐arm study, 24 adults with type 2 diabetes previously treated with basal–bolus or basal‐only insulin regimens and switched to Omnipod 5 achieved a 21.9% increase in TIR 70–180 mg/dL, accompanied by reductions in TAR > 250 mg/dL and minimal TBR < 54 mg/dL [30]. Notably, a further reduction in TAR > 250 mg/dL was observed during the 6‐month extension phase of the same study [31]. More recently, 95 adults with insulin‐requiring type 2 diabetes (61.1% of whom on MDI therapy) initiated on the MiniMed 780G system reported significantly improved HbA1c (7.2% vs. 7.9%) and TIR 70–180 mg/dL (79.8% vs. 72.2%), with consistently low TBR and no episodes of severe hypoglycemia or hyperglycemia over a 3‐month observation period [32]. Taken as a whole, the results of these nonrandomized studies are consistent with our findings, further supporting the efficacy and safety of AID systems in the management of type 2 diabetes.

Our meta‐analysis has some limitations that need to be acknowledged. First, since the included RCTs used different brands of sensors, differences in CGM‐derived outcomes should be interpreted with caution [33]. However, in the three RCTs that collected laboratory‐measured HbA1c at the end of the study, the reduction in HbA1c was consistent and aligned with the observed increase in TIR 70–180 mg/dL, thereby supporting a genuine improvement in glycemic control achieved through AID. Second, substantial heterogeneity among the included studies was found, likely attributable to factors such as small sample sizes, the use of outdated pumps and CGM devices or prototype algorithms in certain studies, differences in study settings (inpatient vs. outpatient, in‐clinic vs. free‐living conditions), and populations (insulin‐treated vs. non‐insulin‐treated, with the inclusion of patients on maintenance dialysis in two studies), and the wide variation in intervention durations (ranging from 1 to 91 days). We adopted broad inclusion criteria to capture all available evidence on AID in type 2 diabetes. Despite the heterogeneity in study settings and populations, the underlying pathophysiology of type 2 diabetes and the shared therapeutic goal of improving glycemic status provide a reasonable clinical rationale for pooling the data from the included studies. To address these potential sources of variability, we performed subgroup and sensitivity analyses stratified by type of AID system, care setting, and study duration; the results were largely consistent with those of the primary analysis, thereby supporting the robustness of our findings across different scenarios. However, a critical appraisal of the results is guaranteed by accurate reporting of the heterogeneity among studies within the framework of evidence assessment according to the GRADE methodology (low‐to‐moderate certainty), the formal evaluation of the credibility of subgroup and metaregression analyses (low‐to‐moderate credibility), and the calculation of PIs for all the selected outcomes. Of note, the PIs encompassed the null effect for all outcomes, although minimally for the main outcome TIR 70–180 mg/dL (−0.07 to 36.92). Hence, further studies are warranted to confirm the findings of this meta‐analysis. Finally, it is important to emphasize that all included studies were of relatively short duration (up to 3 months), and the improvements observed may not necessarily translate into long‐term clinical benefits.

In recent years, the therapeutic armamentarium for type 2 diabetes has expanded with the introduction of innovative agents, including SGLT‐2 inhibitors, glucagon‐like peptide‐1 (GLP‐1) receptor agonists and dual GLP‐1/glucose‐dependent insulinotropic (GIP) receptor agonists, which provide meaningful benefits in terms of HbA1c reduction, weight loss, and prevention of cardiovascular and renal events [3]. However, given the progressive nature of the disease, many individuals eventually require initiation of insulin therapy and treatment intensification through increasingly complex regimens. In this scenario, we believe that AID may represent a valuable option for patients inadequately controlled on MDI therapy to improve glycemic outcomes and reduce hypoglycemia. Improved glycemic outcomes are robustly associated with a reduction in diabetes‐related complications, with potential benefits also in terms of healthcare resource utilization and socioeconomic burden [34, 35]. Our views align with the most recent recommendations of the American Diabetes Association [36], which endorse AID as the preferred method of insulin delivery for adults and children with type 2 diabetes, rather than MDI, CSII, or sensor‐augmented pump therapy. However, the limited Food and Drug Administration (FDA) approval and Conformité Européenne (CE) labelling for use in type 2 diabetes (currently restricted to certain devices), the need for high‐level training of patients and/or their caregivers, and the variability in funding and reimbursement for medical devices across countries are well‐established barriers to implementation in clinical practice [17, 37]. Use of AID (particularly FCL) systems in hospitalized individuals with diabetes is also promising, with the dual aim of optimizing glycemic outcomes and simplifying glucose monitoring and insulin therapy management for healthcare professionals. However, evidence on hospital length of stay and cost‐effectiveness is still limited [38].

## 5 Conclusion

In individuals with type 2 diabetes, AID is associated with short‐term improvements in glycemic control, although the certainty of evidence is low to moderate. Clinical benefits were observed with both HCL and FCL devices, regardless of whether patients were treated in outpatient or inpatient settings. Longer‐term data are needed to support more definitive clinical conclusions.

## Author Contributions

**S.D.M.:** conceptualization, data curation, writing – original draft. **L.D.G.:** conceptualization, methodology, formal analysis, writing – original draft. **I.C.:** methodology, formal analysis, writing – original draft. **M.C.:** data curation, writing – original draft. **S.P.:** writing – review and editing. **A.N.:** writing – review and editing. **L.L.:** writing – review and editing; **F.G.:** conceptualization, funding acquisition, supervision, writing – review and editing.

## Funding

This work was supported by NextGenerationEU.

## Conflicts of Interest

S.D.M.: Ascensia Diabetes Care, MOVI SpA, Roche Diabetes Care (honoraria); Ascensia Diabetes Care, MOVI SpA, Menarini Diagnostics, Roche Diabetes Care, Sanofi Aventis (participation on Advisory Boards). L.D.G.: Eli Lilly, Roche Diabetes Care, MOVI SpA, Sanofi, Theras (honoraria); Abbott, Eli Lilly, Lusofarmaco, Novo Nordisk, Sanofi (support for attending meetings or travels); Eli Lilly (participation on Advisory Boards). I.C.: Eli Lilly, Novo Nordisk, Guidotti SpA, AstraZeneca (honoraria); Eli Lilly, Novo Nordisk, Abbott (support for attending meetings or travels). M.C.: Eli Lilly, Lusofarmaco, Novo Nordisk, Sanofi (support for attending meetings or travels). S.P.: AstraZeneca, Eli Lilly, Novo Nordisk, Sanofi Aventis (honoraria). A.N.: AstraZeneca, Novo Nordisk, and Sanofi Aventis (honoraria). L.L.: Abbott, AstraZeneca, Boehringer‐Ingelheim, Eli Lilly, Merck Sharp & Dohme, Medtronic, Menarini, MOVI SpA, Mundipharma, Novo Nordisk, Roche Diabetes Care, Sanofi Aventis, Terumo (honoraria); Abbott, AstraZeneca, Boeringher‐Ingelheim, Eli Lilly Italia, Medtronic, MOVI SpA, Novo Nordisk, Roche Diabetes Care, Sanofi Aventis, Terumo (participation on Advisory Boards). F.G.: Eli Lilly, Roche Diabetes Care (grants); Eli Lilly, Novo Nordisk (consulting fees); AstraZeneca, Boehringer‐Ingelheim, Eli Lilly, Lifescan, Merck Sharp & Dohme, Medtronic, Novo Nordisk, Roche Diabetes Care, Sanofi Aventis (support for attending meetings or travels); AstraZeneca, Biomea, Boehringer‐Ingelheim, Eli Lilly, Lifescan, Merck Sharp & Dohme, Medtronic, Novo Nordisk, Roche Diabetes Care, Sanofi Aventis (participation on Advisory Boards); EASD/EFSD, Società Italiana di Endocrinologia (SIE), Fo.Ri.SIE (unpaid leadership); AstraZeneca, Eli Lilly, Novo Nordisk, Sanofi Aventis (support for medical writing and statistical analysis).

## Supporting information


**Appendix S1–S12:** dom70708‐sup‐0001‐Appendix.pdf.

## Data Availability

All data relevant to the study are included in the article or uploaded as Supporting Information. Statistical code and data set available on reasonable request from Prof. Francesco Giorgino (francesco.giorgino@uniba.it).
