# Supplementary material for: Efficacy and Safety of Automated Insulin Delivery in People With Type 2 Diabetes: A Systematic Review and Meta‐Analysis
Source: Diabetes Obes Metab. 2026 Mar 27;28(6):5091–100. doi: 10.1111/dom.70708 (PMC13146134; doi:10.1111/dom.70708)
Supplement: Supplementary file 1 — Appendix S1–S12: dom70708‐sup‐0001‐Appendix.pdf. [file DOM-28-5091-s001.pdf]

Supplementary Appendix

Efficacy and safety of closed loop insulin delivery in people with type 2 diabetes: a systematic review and meta-analysis

Summary

Supplement 1. Search strategy **3**
1.1 Excluded studies 5
Supplement 2. Characteristics of included studies and patients' baseline features **7**
Supplement 3. Risk of bias assessment of included trials for each outcome **9**
3.1 Time In Range (%) 9
3.2 Time Below Range (%) 10
3.3 Time Below Range <54 mg/dL (%) 11
3.4 Time Above Range (%) 12
3.5 Time Above Range >250 mg/dL (%) 13
3.6 Mean glucose (mg/dL) 14
3.7 Coefficient of Variation (%) 15
3.8 Standard Deviation (mg/dL) 16
3.9 HbA1c 17
3.10 Total daily insulin dose (UI) 18
3.11 Change in body weight (kg) 19
Supplement 4. Funnel plots **20**
Supplement 5. Forest plots **26**
5.1. Overall analysis 26
5.2. Sensitivity analysis restricted to studies with intervention duration ≥2 weeks 30
5.3. Sensitivity analysis restricted to studies with low risk of bias 34
5.4. Sensitivity analysis restricted to studies with no imputed standard deviations 37
5.5. Sensitivity analysis restricted to studies comparing closed-loop insulin delivery with other types of insulin therapy 37
Supplement 6. Summary of findings **40**
Supplement 7. Subgroup analysis, fully closed-loop vs. hybrid closed-loop **42**
Supplement 8. Subgroup analysis, inpatient vs. outpatient care setting **57**
Supplement 9. Meta-regression analysis **68**
Supplement 10. Severe adverse events **74**
Supplement 11. Patient-reported outcomes measures **75**
Supplement 12. Prisma 2020 Checklist **76**
References **79**

Supplement 1. Search strategy

Searches:
MEDLINE (via Ovid)
CENTRAL
Web of Science
Pubmed (hand searching)
From inception to 1st July 2025
No language restriction

MEDLINE (via Ovid):

1. "diabet*".mp.
2. Diabetes Mellitus/
3. exp Diabetes Mellitus Type 2/
4. ((diabetes or "diabetes mellitus" or diabetic*) and ("type 2" or "type II" or "type ii")).mp. [mp=ti, ab, tx, ct, ot, hw, cw, bt, nm, fx, kf, ox, px, rx, ui, sy, ux, mx]
5. "Minimed 780g".mp.
6. "780G".mp.
7. "Minimed 670g".mp.
8. "670G".mp.
9. "controlIQ".mp.
10. "control-IQ".mp.
11. "control IQ".mp.
12. "CamAPS Fx".mp.
13. "CamAPS Hx".mp.
14. "CamAPS".mp.
15. "DBLG1".mp.
16. "Diabeloop".mp.
17. "omnipod 5".mp.
18. "omnipod5".mp.
19. "omnipod".mp.
20. "iLet Bionic".mp.
21. "Bionic pancreas".mp.
22. "automated insulin delivery".mp.
23. "hybrid closed loop".mp.
24. "closed loop".mp.
25. "hybrid closed-loop".mp.
26. "closed-loop".mp.
27. "full closed-loop".mp.
28. "full closed loop".mp.
29. "fully closed-loop".mp.
30. "fully closed loop".mp.
31. randomized controlled trial.pt.
32. controlled clinical trial.pt.

33. pragmatic clinical trial.pt.
34. randomized.ab.
35. randomised.ab.
36. randomly.ab.
37. or/1-4
38. or/5-30
39. or/31-36
40. and/37-39
41. remove duplicates from 40

URL to search strategy:
https://ovidsp.ovid.com/ovidweb.cgi?T=JS&NEWS=N&PAGE=main&SHAREDSEARCHID=2I0ETO8RaIgLzcFdZ8ZlgZWD4VBnBr46CIRZpoHrGLvFqdErLhcIbbAounsa1zb8G

CENTRAL:
(diabet* OR "diabetes mellitus" OR "type 2 diabetes" OR "type 2 diabetes mellitus" OR "type II diabetes" OR "type II diabetes mellitus")
AND
("Minimed 780g" OR "780G" OR "Minimed 670g" OR "670G" OR "Control IQ" OR "Control-IQ" OR "CamAPS Fx" OR “CamAPS Hx” OR “CamAPS” OR "DBLG1" OR "Diabeloop" OR "Omnipod 5" OR "Omnipod5" OR "Omnipod" OR "iLet Bionic" OR "Bionic Pancreas" OR "automated insulin delivery" OR "hybrid closed loop" OR "closed loop" OR "hybrid closed-loop" OR "closed-loop" OR “full closed loop” OR “full closed-loop” OR “fully closed loop” OR “fully closed-loop”)
AND
(randomized OR randomised OR randomly OR trial OR "controlled trial" OR "clinical trial")
in all text

Web of Science:
(diabet* OR "diabetes mellitus" OR "type 2 diabetes" OR "type 2 diabetes mellitus" OR "type II diabetes" OR "type II diabetes mellitus")
AND
("Minimed 780g" OR "780G" OR "Minimed 670g" OR "670G" OR "Control IQ" OR "Control-IQ" OR "CamAPS Fx" OR “CamAPS Hx” OR “CamAPS” OR "DBLG1" OR "Diabeloop" OR "Omnipod 5" OR "Omnipod5" OR "Omnipod" OR "iLet Bionic" OR "Bionic Pancreas" OR "automated insulin delivery" OR "hybrid closed loop" OR "closed loop" OR "hybrid closed-loop" OR "closed-loop" OR “full closed loop” OR “full closed-loop” OR “fully closed loop” OR “fully closed-loop”)
AND
(randomized OR randomised OR randomly OR trial OR "controlled trial" OR "clinical trial")

Pubmed:
(diabet* OR "diabetes mellitus" OR "type 2 diabetes" OR "type 2 diabetes mellitus" OR "type II diabetes" OR "type II diabetes mellitus")

AND ("Minimed 780G" OR "780G" OR "Minimed 670G" OR "670G" OR "Control IQ" OR "Control-IQ" OR "CamAPS Fx" OR “CamAPS Hx” OR “CamAPS” OR "DBLG1" OR "Diabeloop" OR "Omnipod 5" OR "Omnipod5" OR "Omnipod" OR "iLet Bionic" OR "Bionic Pancreas" OR "automated insulin delivery" OR "hybrid closed loop" OR "closed loop" OR "hybrid closed-loop" OR "closed-loop" OR “full closed loop” OR “full closed-loop” OR “fully closed loop” OR “fully closed-loop”)
AND ("randomized controlled trial" OR "randomised controlled trial" OR "randomized trial" OR "randomised trial" OR RCT)

1.1 Excluded studies

<u>Not outcome of interest</u>

1. Kingman RS et al., 2017, Diabetes
2. Kumareswaran K et al., 2013, Diabetes
3. Thabit H et al., 2014, Journal of Clinical Endocrinology & Metabolism

<u>Not eligible intervention</u>

1. Hovorka R et al., 2007, J Clin Endocrinol Metab
2. Leelarathna L et al., 2013, Critical Care

<u>Not population of interest</u>

1. Amadou C et al., 2023, PLOS ONE
2. Biester T et al., 2017, Diabetes Technology & Therapeutics
3. Biester T et al., 2016, Pediatric Diabetes
4. Boughton CK et al., 2019, Diabetes Technology & Therapeutics
5. Crocket H et al., 2023, Diabetes Technology & Therapeutics
6. Forlenza GP et al., 2017, Diabetes Care
7. Krutyte G et al., 2025, Annals of Surgery

<u>Not study design of interest</u>

1. Bally L et al., 2019, Diabetologia
2. Bally L et al., 2019, Diabetes Obesity & Metabolism
3. Boughton CK et al., 2023, Diabetic Medicine
4. Garelli F et al., 2023, J Diabetes Sci Technol
5. Kadiyala N et al., 2024, Journal of Diabetes Science & Technology
6. Pasquel FJ et al., 2025, JAMA Network Open
7. Usoh CO et al., 2023, Endocrine Practice
8. Wilkinson T et al., 2025, Diabetes Technology & Therapeutics

<u>Not publication type of interest</u>

1. Karol AB et al., 2023, Endocrine Practice
2. Amer BE et al., 2024, Diabetic Medicine

Duplicates

1. Bally L et al., 2019, Diabetes
2. Bally L et al., 2018, Diabetologia
3. Boughton CK et al., 2019, The Lancet Diabetes & Endocrinology
4. Boughton CK et al., 2021, Nature Medicine
5. Boughton CK et al., 2022, Diabetologia
6. Boughton CK et al., 2021, Diabetes
7. Thabit H et al., 2016, Diabetes
8. Thabit H et al., 2017, The Lancet Diabetes & Endocrinology
9. Herzig D et al., 2022, Diabetes Care
10. Plank J et al., 2006, Diabetes Care
11. Reznik Y, 2024, Diabetes Technology & Therapeutics
12. Reznik Y et al., 2024, Diabetes Obesity & Metabolism

Supplement 2. Characteristics of included studies and patients' baseline features

Author	Year	Study design	HCL system	Type of closed loop	Comparator	Duration of intervention (days)	No. of participants	Type of participants	Female (%)	Age (years)	Diabetes duration (years)	Insulin treatment (%)	Prior CGM use (%)	HbA1c (%)
Kumareswaran K[1]	2014	crossover	FreeStyle Navigator+ Animas 2020+ Cambridge MPC algorithm versions 0.03.20–0.03.23	fully	oral anti-diabetes medications + CGM	1	12	outpatients	41.7	57.2±14.4	7.6±6.1	0	0	8.4±0.8
Bally L[2]	2018	parallel	Freestyle Navigator II + Dana Diabecare R+ Cambridge MPC algorithm version 0.3.70	fully	insulin injections	7.2±3.9	136	inpatients	31.6	67.4±11.5	16.3±11.2	100	0	8.1±1.9
Taleb N[3]	2019	crossover	Dexcom G4+Accu-Chek Combo+MPC algorithm	hybrid	MDI therapy + CGM	1	15	outpatients	26.7	63.6±6.7	NA	100	0	7.85±0.6%
Boughton CK[4]	2021	crossover	Dexcom G6+Dana Diabecare RS+Cambridge MPC algorithm version 0.3.71 (CamAPS HX)	fully	MDI therapy	20	27	outpatients	37	68.3±11.2	20.0±10.0	100	0	7.2±1.3

Author	Year	Study design	HCL system	Type of closed loop	Comparator	Duration of intervention (days)	No. of participants	Type of participants	Female (%)	Age (years)	Diabetes duration (years)	Insulin treatment (%)	Prior CGM use (%)	HbA1c (%)
Herzig D[5]	2022	parallel	Dexcom G6+Dana Diabecare RS+Cambridge MPC algorithm version 0.3.71 (CamAPS HX)	fully	standard insulin therapy	8.9±5.5	44	inpatients	34.1	68.5±12.3	10.2±8.4	54.5	0	7.6±1.9
Daly AB[6]	2023	crossover	Dexcom G6+Dana Diabecare RS+Cambridge MPC algorithm version 0.3.71 (CamAPS HX)	fully	MDI therapy	56	26	outpatients	27	59±11	17.5±8.2	100	12	9.0±1.4
Reznik Y[7]	2023	parallel	Dexcom G6+Tandem t:slim X2 w/ Control-IQ	hybrid	MDI therapy	90	29	outpatients	69	69.5±8.6	18.6±10.6	100	44.8	9.1±1.1
Borel A[8]	2024	crossover	Dexcom G6+Accu-Chek Insight w/ DBLG1	hybrid	CSII + CGM	42	17	outpatients	35.3	63±9	24±9	100	100	7.9±0.9
Kudva YC[9]	2025	parallel	Dexcom G6+Tandem t:slim X2 w/ Control-IQ	hybrid	MDI 96%, CSII 4% ± CGM	91	319	outpatients	48.3	58.3±12	18.1±10.6	100	70.3	8.2±1.3

CGM, continuous glucose monitoring; CSII, continuous subcutaneous insulin infusion; MDI, multiple daily insulin injections; MPC, model-predictive control.

Supplement 3. Risk of bias assessment of included trials for each outcome

3.1 Time In Range (%)

Study design: parallel arms

Study ID	Randomization process	Deviations from intended intervention	Missing outcome data	Measurement of outcome	Selection of reported result	Overall
Bally L 2021	Some concerns	Low risk	Low risk	Low risk	Low risk	Some concerns
Herzig D 2022	Low risk	Low risk	Low risk	Low risk	Low risk	Low risk
Kudva YC 2025	Low risk	Low risk	Low risk	Low risk	Low risk	Low risk
Reznic Y 2023	Low risk	Low risk	Some concerns	Low risk	Low risk	Some concerns

Study design: cross-over

Study ID	Randomization process	Period and carryover effects	Deviations from intended intervention	Missing outcome data	Measurement of outcome	Selection of the reported result	Overall
Borel AL 2024	Low risk	Low risk	Low risk	Low risk	Low risk	Low risk	Low risk
Boughton CK 2021	Low risk	Low risk	Low risk	Low risk	Low risk	Low risk	Low risk
Daly AB 2023	Low risk	Low risk	Low risk	Low risk	Low risk	Low risk	Low risk
Kumareswaran K 2014	Some concerns	Low risk	Low risk	Some concerns	Low risk	Low risk	Some concerns
Taleb N 2019	Some concerns	Low risk	Low risk	Low risk	Low risk	Low risk	Some concerns

3.2 Time Below Range (%)

Study design: parallel arms

**Study ID**	**Randomization process**	**Deviations from intended intervention**	**Missing outcome data**	**Measurement of outcome**	**Selection of reported result**	**Overall**
Bally L 2021	Some concerns	Low risk	Low risk	Low risk	Low risk	Some concerns
Herzig D 2022	Low risk	Low risk	Low risk	Low risk	Low risk	Low risk
Kudva YC 2025	Low risk	Low risk	Low risk	Low risk	Low risk	Low risk
Reznic Y 2023	Low risk	Low risk	Some concerns	Some concerns	Low risk	Some concerns

Study design: cross-over

**Study ID**	**Randomization process**	**Period and carryover effects**	**Deviations from intended intervention**	**Missing outcome data**	**Measurement of outcome**	**Selection of the reported result**	**Overall**
Borel AL 2024	Low risk	Low risk	Low risk	Low risk	Low risk	Low risk	Low risk
Boughton CK 2021	Low risk	Low risk	Low risk	Low risk	Low risk	Low risk	Low risk
Daly AB 2023	Low risk	Low risk	Low risk	Low risk	Low risk	Low risk	Low risk
Kumareswaran K 2014	Some concerns	Low risk	Low risk	Some concerns	Low risk	Low risk	Some concerns
Taleb N 2019	Some concerns	Low risk	Low risk	Low risk	Low risk	Low risk	Some concerns

3.3 Time Below Range <54 mg/dL (%)

Study design: parallel arms

**Study ID**	**Randomization process**	**Deviations from intended intervention**	**Missing outcome data**	**Measurement of outcome**	**Selection of reported result**	**Overall**
Bally L 2021	Some concerns	Low risk	Low risk	Low risk	Low risk	Some concerns
Herzig D 2022	Low risk	Low risk	Low risk	Low risk	Low risk	Low risk
Kudva YC 2025	Low risk	Low risk	Low risk	Low risk	Low risk	Low risk
Reznic Y 2023	Low risk	Low risk	Some concerns	Some concerns	Low risk	Some concerns

Study design: cross-over

**Study ID**	**Randomization process**	**Period and carryover effects**	**Deviations from intended intervention**	**Missing outcome data**	**Measurement of outcome**	**Selection of the reported result**	**Overall**
Borel AL 2024	Low risk	Low risk	Low risk	Low risk	Low risk	Some concerns	Some concerns
Boughton CK 2021	Low risk	Low risk	Low risk	Low risk	Low risk	Low risk	Low risk
Daly AB 2023	Low risk	Low risk	Low risk	Low risk	Low risk	Low risk	Low risk

3.4 Time Above Range (%)

Study design: parallel arms

**Study ID**	**Randomization process**	**Deviations from intended intervention**	**Missing outcome data**	**Measurement of outcome**	**Selection of reported result**	**Overall**
Bally L 2021	Some concerns	Low risk	Low risk	Low risk	Low risk	Some concerns
Herzig D 2022	Low risk	Low risk	Low risk	Low risk	Low risk	Low risk
Kudva YC 2025	Low risk	Low risk	Low risk	Low risk	Low risk	Low risk
Reznic Y 2023	Low risk	Low risk	Some concerns	Some concerns	Low risk	Some concerns

Study design: cross-over

**Study ID**	**Randomization process**	**Period and carryover effects**	**Deviations from intended intervention**	**Missing outcome data**	**Measurement of outcome**	**Selection of the reported result**	**Overall**
Borel AL 2024	Low risk	Low risk	Low risk	Low risk	Low risk	Low risk	Low risk
Boughton CK 2021	Low risk	Low risk	Low risk	Low risk	Low risk	Low risk	Low risk
Daly AB 2023	Low risk	Low risk	Low risk	Low risk	Low risk	Low risk	Low risk
Kumareswaran K 2014	Some concerns	Low risk	Low risk	Some concerns	Low risk	Low risk	Some concerns
Taleb N 2019	Some concerns	Low risk	Low risk	Low risk	Low risk	Low risk	Some concerns

3.5 Time Above Range >250 mg/dL (%)

Study design: parallel arms

**Study ID**	**Randomization process**	**Deviations from intended intervention**	**Missing outcome data**	**Measurement of outcome**	**Selection of reported result**	**Overall**
Kudva YC 2025	Low risk	Low risk	Low risk	Low risk	Low risk	Low risk
Reznic Y 2023	Low risk	Low risk	Some concerns	Low risk	Low risk	Some concerns

Study design: cross-over

**Study ID**	**Randomization process**	**Period and carryover effects**	**Deviations from intended intervention**	**Missing outcome data**	**Measurement of outcome**	**Selection of the reported result**	**Overall**
Borel AL 2024	Low risk	Low risk	Low risk	Low risk	Low risk	Some concerns	Low risk

3.6 Mean glucose (mg/dL)

**Study design: parallel arms**						
**Study ID**	**Randomization process**	**Deviations from intended intervention**	**Missing outcome data**	**Measurement of outcome**	**Selection of reported result**	**Overall**
Bally L 2021	Some concerns	Low risk	Low risk	Low risk	Low risk	Some concerns
Herzig D 2022	Low risk	Low risk	Low risk	Low risk	Low risk	Low risk
Kudva YC 2025	Low risk	Low risk	Low risk	Low risk	Low risk	Low risk

**Study design: cross-over**							
**Study ID**	**Randomization process**	**Period and carryover effects**	**Deviations from intended intervention**	**Missing outcome data**	**Measurement of outcome**	**Selection of the reported result**	**Overall**
Borel AL 2024	Low risk	Low risk	Low risk	Low risk	Low risk	Some concerns	Some concerns
Boughton CK 2021	Low risk	Low risk	Low risk	Low risk	Low risk	Low risk	Low risk
Daly AB 2023	Low risk	Low risk	Low risk	Low risk	Low risk	Low risk	Low risk
Kumareswaran K 2014	Some concerns	Low risk	Low risk	Some concerns	Low risk	Low risk	Some concerns
Taleb N 2019	Some concerns	Low risk	Low risk	Low risk	Low risk	Low risk	Some concerns

3.7 Coefficient of Variation (%)

**Study design: parallel arms**						
**Study ID**	**Randomization process**	**Deviations from intended intervention**	**Missing outcome data**	**Measurement of outcome**	**Selection of reported result**	**Overall**
Bally L 2021	Some concerns	Low risk	Low risk	Low risk	Low risk	Some concerns
Herzig D 2022	Low risk	Low risk	Low risk	Low risk	Low risk	Low risk
Kudva YC 2025	Low risk	Low risk	Low risk	Low risk	Low risk	Low risk
Reznic Y 2023	Low risk	Low risk	Some concerns	Some concerns	Low risk	Some concerns

**Study design: cross-over**							
**Study ID**	**Randomization process**	**Period and carryover effects**	**Deviations from intended intervention**	**Missing outcome data**	**Measurement of outcome**	**Selection of the reported result**	**Overall**
Borel AL 2024	Low risk	Low risk	Low risk	Low risk	Low risk	Low risk	Low risk
Boughton CK 2021	Low risk	Low risk	Low risk	Low risk	Low risk	Low risk	Low risk
Daly AB 2023	Low risk	Low risk	Low risk	Low risk	Low risk	Low risk	Low risk

3.8 Standard Deviation (mg/dL)

**Study design: parallel arms**							
**Study ID**	**Randomization process**	**Deviations from intended intervention**	**Missing outcome data**	**Measurement of outcome**	**Selection of reported result**	**Overall**	
Bally L 2021	Some concerns	Low risk	Low risk	Low risk	Low risk	Some concerns	
Herzig D 2022	Low risk	Low risk	Low risk	Low risk	Low risk	Low risk	
**Study design: cross-over**							
**Study ID**	**Randomization process**	**Period and carryover effects**	**Deviations from intended intervention**	**Missing outcome data**	**Measurement of outcome**	**Selection of the reported result**	**Overall**
Borel AL 2024	Low risk	Low risk	Low risk	Low risk	Low risk	Low risk	Low risk
Boughton CK 2021	Low risk	Low risk	Low risk	Low risk	Low risk	Low risk	Low risk
Daly AB 2023	Low risk	Low risk	Low risk	Low risk	Low risk	Low risk	Low risk
Kumareswaran K 2014	Some concerns	Low risk	Low risk	Some concerns	Low risk	Low risk	Some concerns

3.9 HbA1c

Study design: parallel arms

Study ID	Randomization process	Deviations from intended intervention	Missing outcome data	Measurement of outcome	Selection of reported result	Overall
Kudva YC 2025	Low risk	Low risk	Low risk	Low risk	Low risk	Low risk
Reznic Y 2023	Low risk	Low risk	Some concerns	Low risk	Low risk	Some concerns

Study design: cross-over

Study ID	Randomization process	Period and carryover effects	Deviations from intended intervention	Missing outcome data	Measurement of outcome	Selection of the reported result	Overall
Daly AB 2023	Low risk	Some concerns	Low risk	Low risk	Low risk	Low risk	Low risk

3.10 Total daily insulin dose (UI)

Study design: parallel arms						
**Study ID**	**Randomization process**	**Deviations from intended intervention**	**Missing outcome data**	**Measurement of outcome**	**Selection of reported result**	**Overall**
Bally L 2021	Some concerns	Some concerns	Low risk	Some concerns	Low risk	Some concerns
Herzig D 2022	Low risk	Low risk	Low risk	Some concerns	Low risk	Some concerns
Kudva YC 2025	Low risk	Low risk	Low risk	Some concerns	Low risk	Some concerns
Reznic Y 2023	Low risk	Low risk	Some concerns	Some concerns	Low risk	Some concerns

Study design: cross-over							
**Study ID**	**Randomization process**	**Period and carryover effects**	**Deviations from intended intervention**	**Missing outcome data**	**Measurement of outcome**	**Selection of the reported result**	**Overall**
Borel AL 2024	Low risk	Low risk	Low risk	Low risk	Low risk	Low risk	Low risk
Boughton CK 2021	Low risk	Low risk	Low risk	Low risk	Low risk	Low risk	Low risk
Daly AB 2023	Low risk	Low risk	Low risk	Low risk	Low risk	Low risk	Low risk
Taleb N 2019	Some concerns	Low risk	Low risk	Low risk	Some concerns	Low risk	Some concerns

3.11 Change in body weight (kg)

**Study design: parallel arms**						
**Study ID**	**Randomization process**	**Deviations from intended intervention**	**Missing outcome data**	**Measurement of outcome**	**Selection of reported result**	**Overall**
Kudva YC 2025	Low risk	Low risk	Low risk	Low risk	Low risk	Low risk
Reznic Y 2023	Low risk	Low risk	Low risk	Low risk	Low risk	Low risk

Supplement 4. Funnel plots

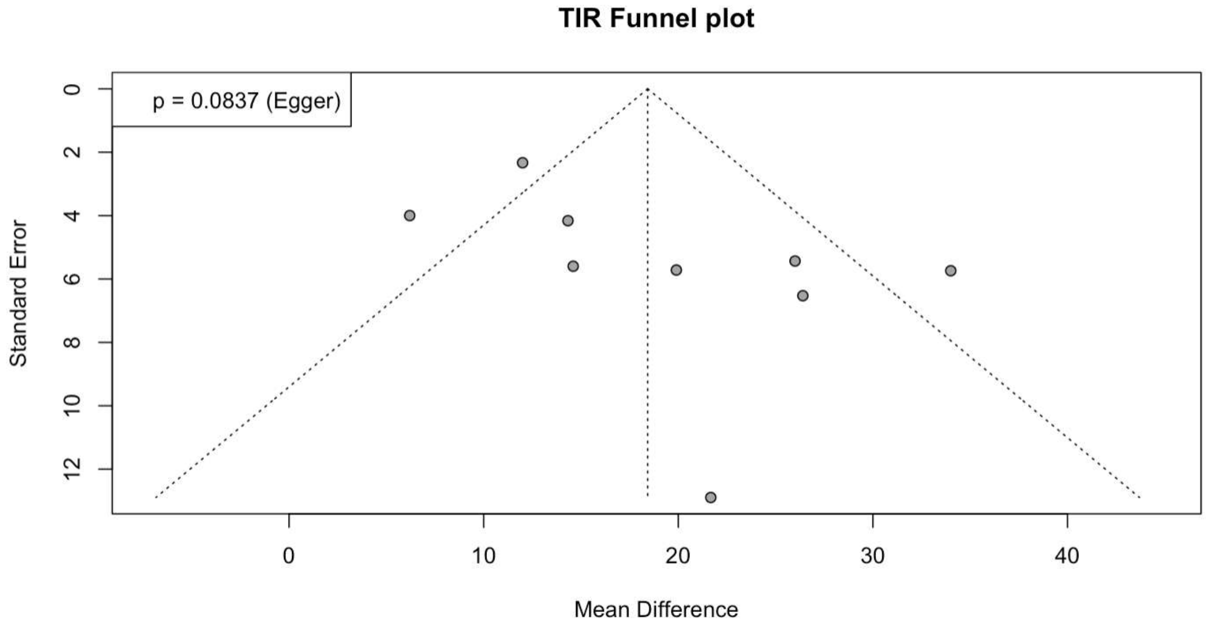

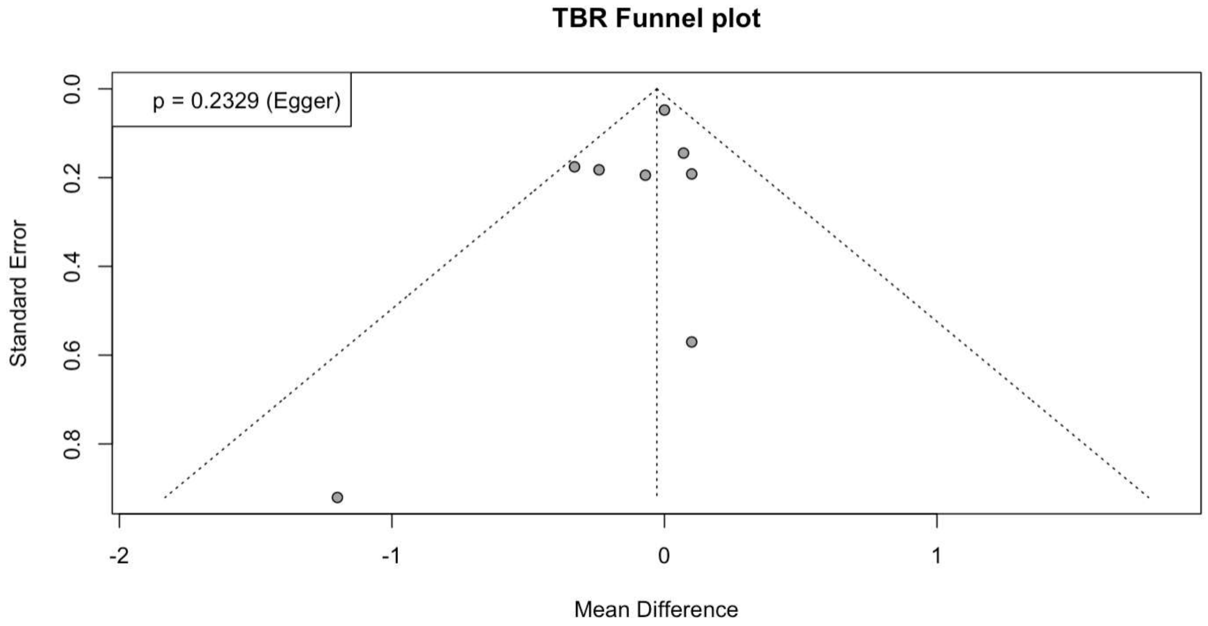

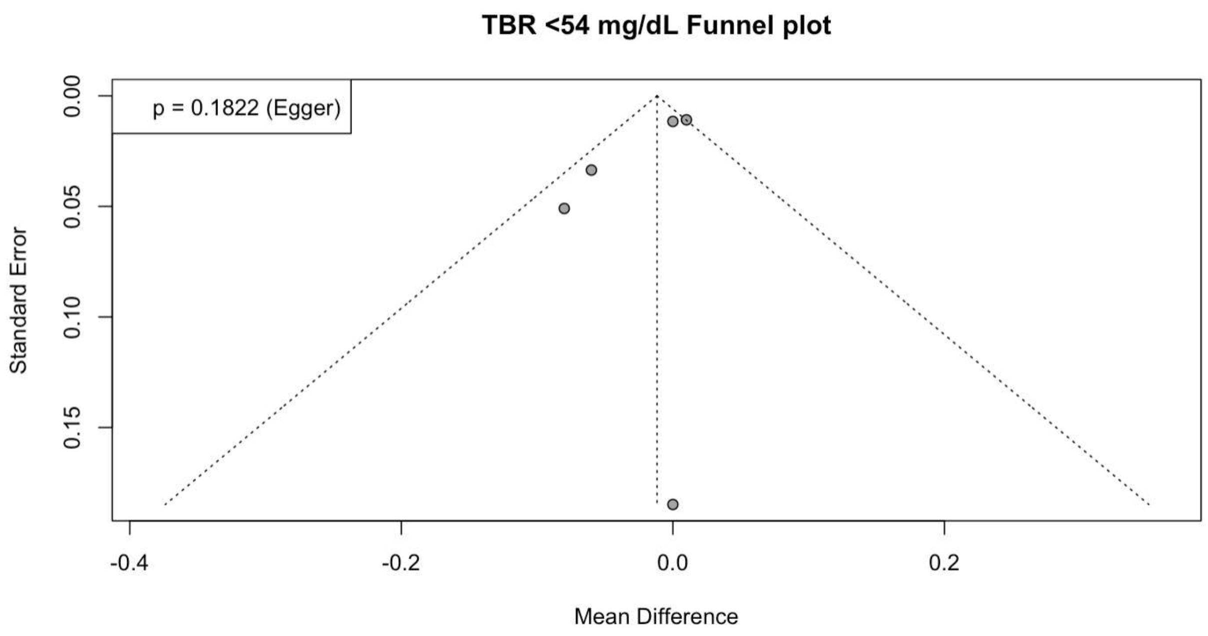
TBR <54 mg/dL Funnel plot
p = 0.1822 (Egger)
Standard Error
0.00
0.05
0.10
0.15
-0.4
-0.2
0.0
0.2
Mean Difference

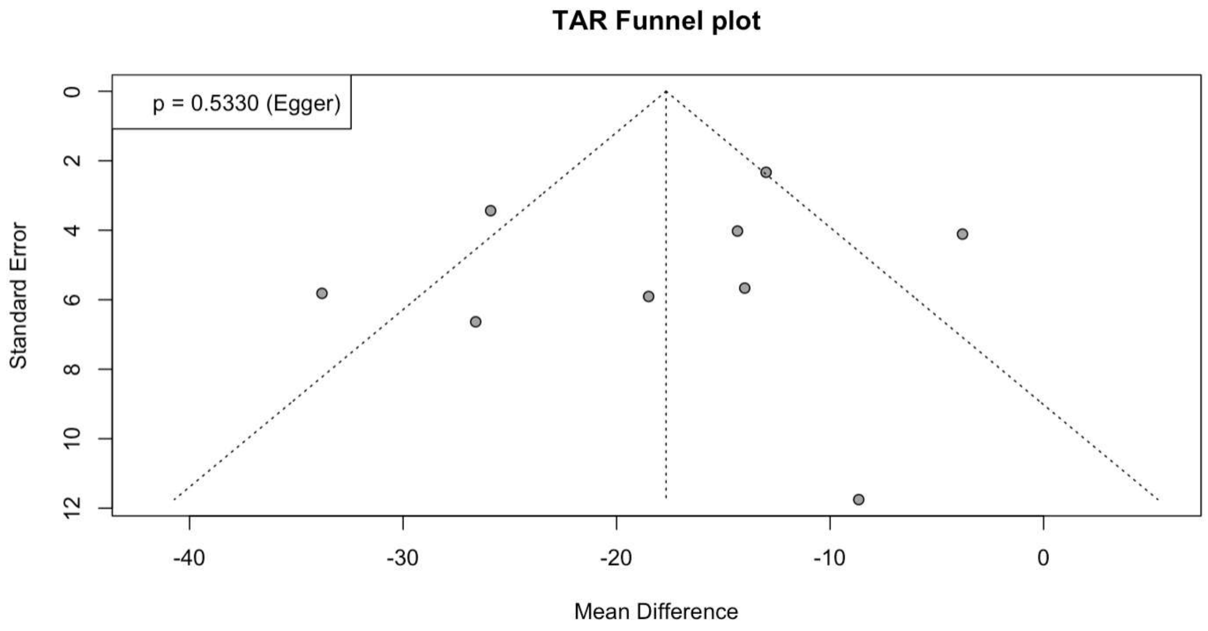
TAR Funnel plot
p = 0.5330 (Egger)
Standard Error
0
2
4
6
8
10
12
-40
-30
-20
-10
0
Mean Difference

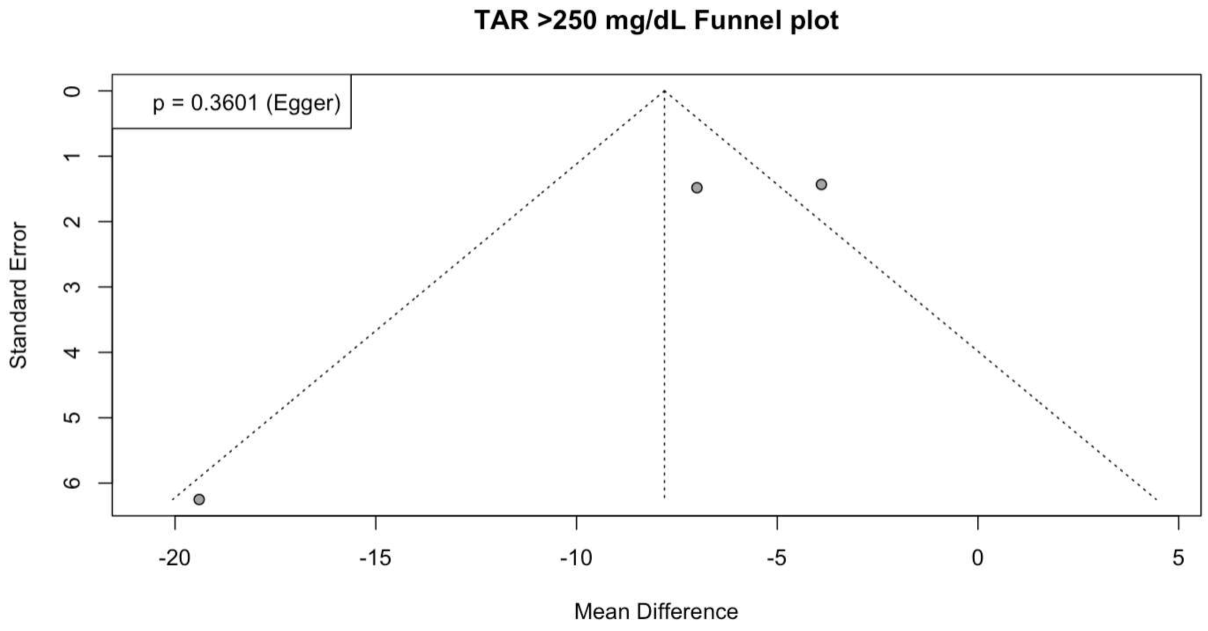
TAR >250 mg/dL Funnel plot
p = 0.3601 (Egger)
Standard Error
0
1
2
3
4
5
6
-20
-15
-10
-5
0
5
Mean Difference

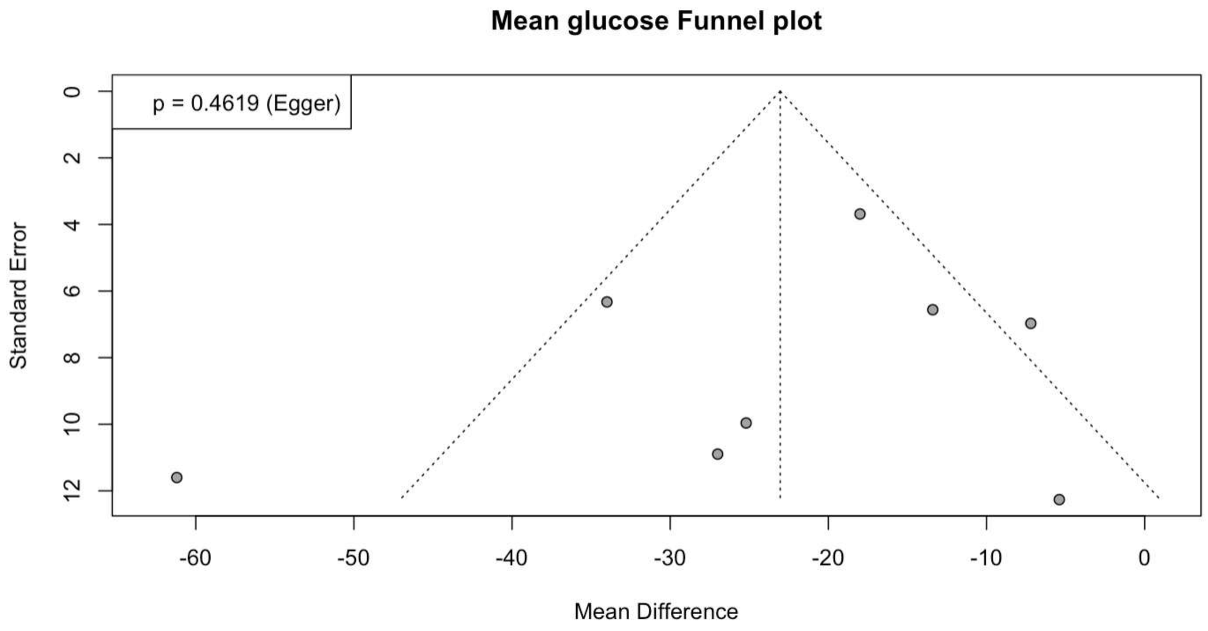
Mean glucose Funnel plot
p = 0.4619 (Egger)
Standard Error
0
2
4
6
8
10
12
-60
-50
-40
-30
-20
-10
0
Mean Difference

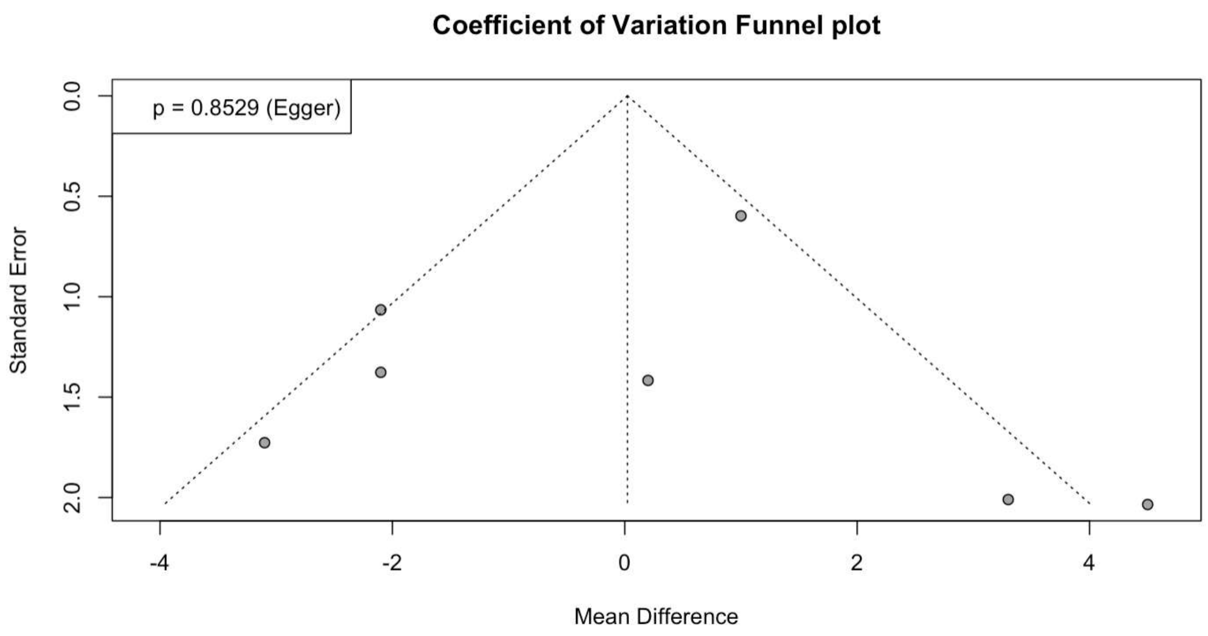
Coefficient of Variation Funnel plot
p = 0.8529 (Egger)
Standard Error
0.0
0.5
1.0
1.5
2.0
-4
-2
0
2
4
Mean Difference

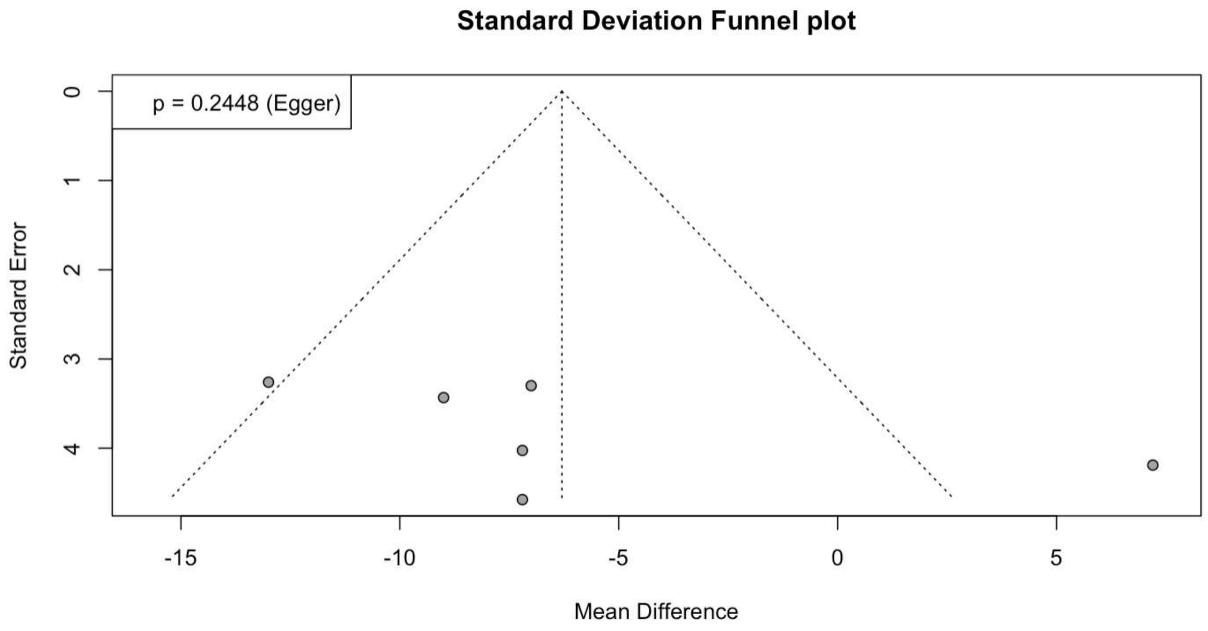
Standard Deviation Funnel plot
p = 0.2448 (Egger)
Standard Error
0
1
2
3
4
-15
-10
-5
0
5
Mean Difference

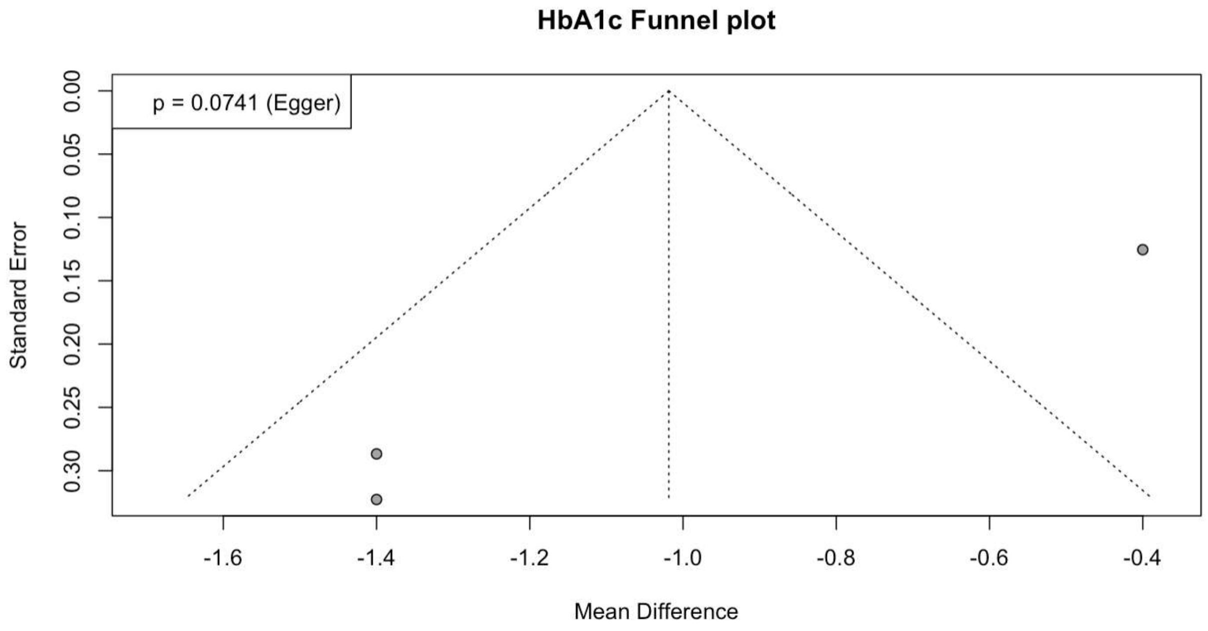
HbA1c Funnel plot
p = 0.0741 (Egger)
Standard Error
0.00
0.05
0.10
0.15
0.20
0.25
0.30
-1.6
-1.4
-1.2
-1.0
-0.8
-0.6
-0.4
Mean Difference

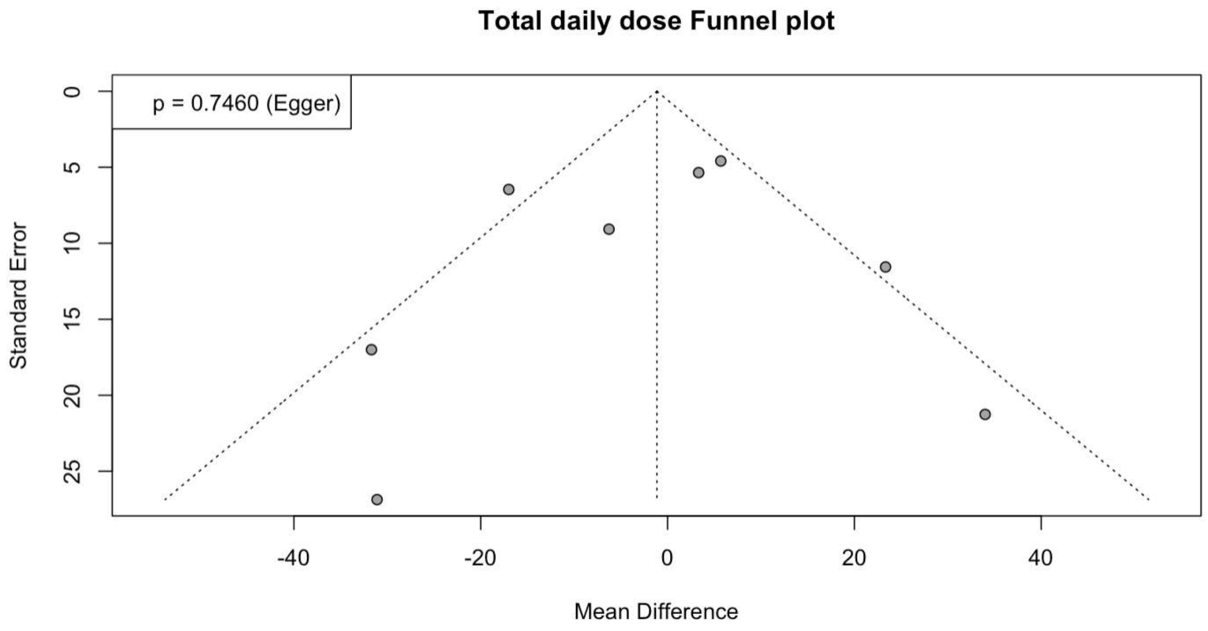
Total daily dose Funnel plot
p = 0.7460 (Egger)
Standard Error
0
5
10
15
20
25
-40
-20
0
20
40
Mean Difference

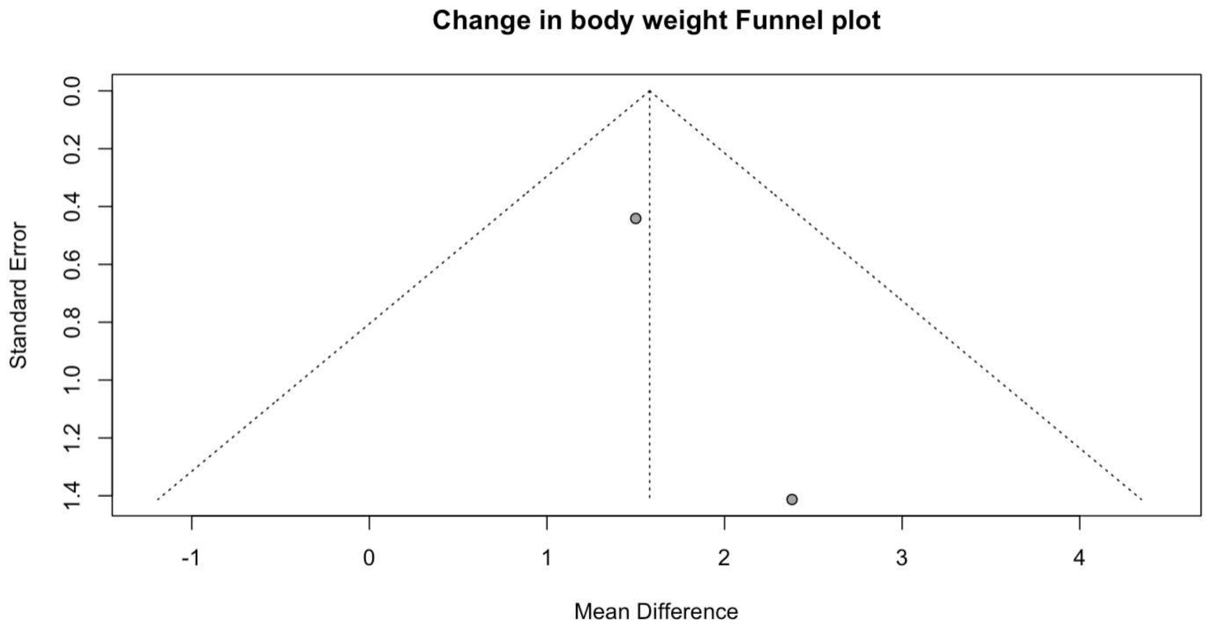
Change in body weight Funnel plot
Standard Error
0.0
0.2
0.4
0.6
0.8
1.0
1.2
1.4
-1
0
1
2
3
4
Mean Difference

Supplement 5. Forest plots

5.1. Overall analysis

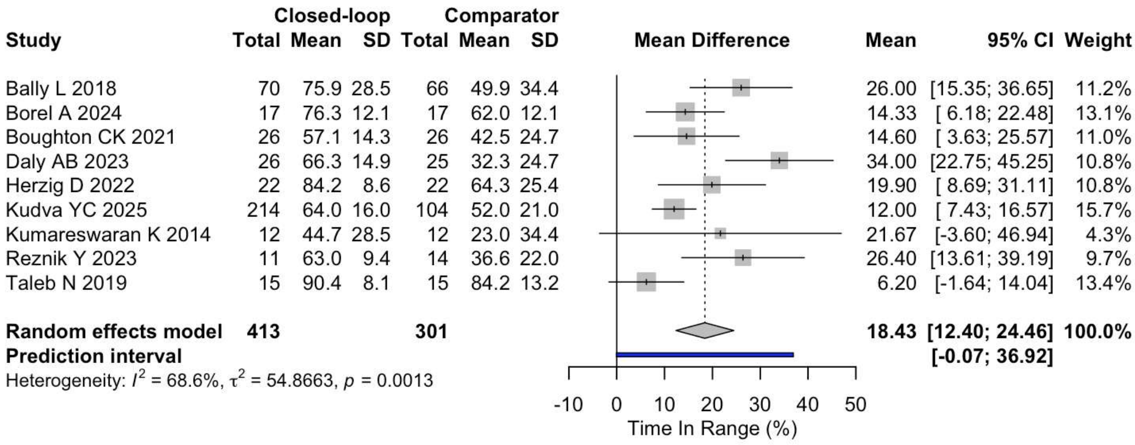

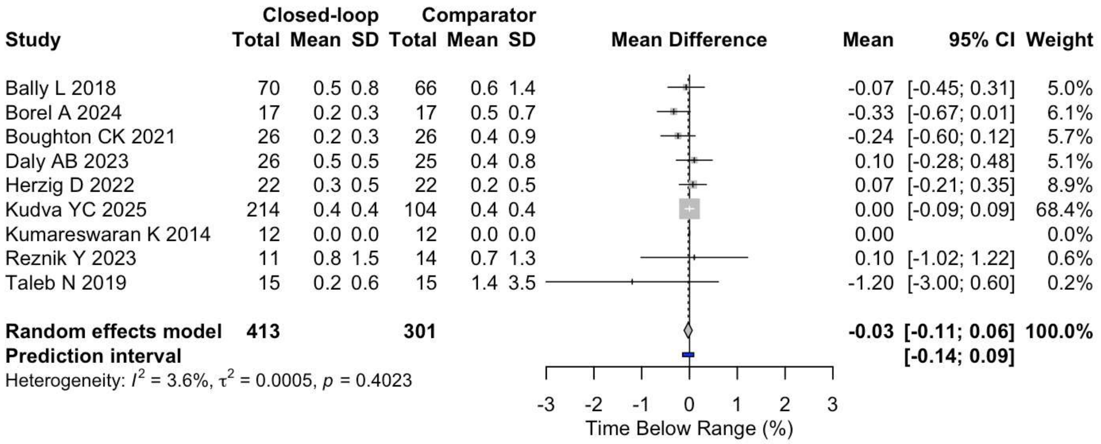

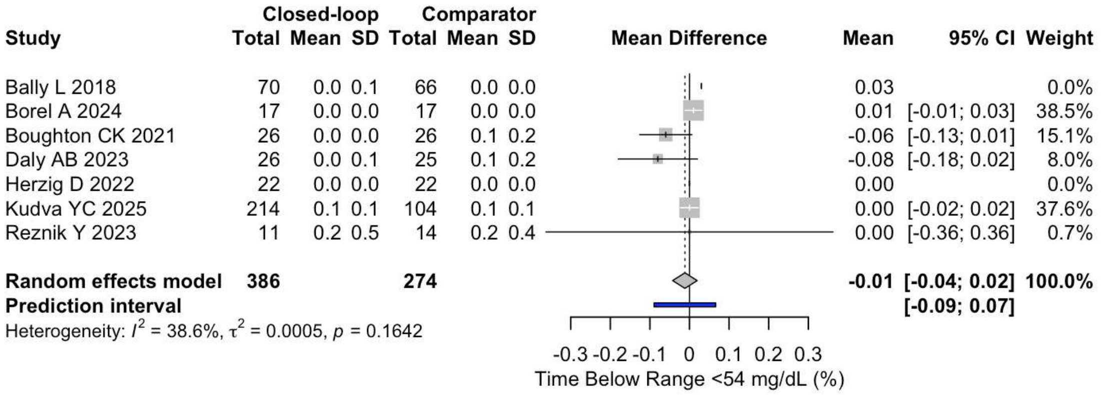

Study	Closed-loop Total	Closed-loop Mean	Closed-loop SD	Comparator Total	Comparator Mean	Comparator SD	Mean	95% CI	Weight
Bally L 2018	70	23.6	16.6	66	49.5	22.8	-25.90	[-32.64; -19.16]	13.6%
Borel A 2024	17	23.3	11.3	17	37.7	12.1	-14.34	[-22.23; -6.45]	12.8%
Boughton CK 2021	26	42.6	14.3	26	56.6	25.1	-14.00	[-25.10; -2.90]	10.7%
Daly AB 2023	26	33.2	14.8	25	67.0	25.2	-33.80	[-45.20; -22.40]	10.5%
Herzig D 2022	22	15.4	8.4	22	33.9	26.4	-18.50	[-30.08; -6.92]	10.4%
Kudva YC 2025	214	35.0	16.0	104	48.0	21.0	-13.00	[-17.57; -8.43]	14.8%
Kumareswaran K 2014	12	27.7	21.8	12	36.3	34.4	-8.66	[-31.69; 14.37]	5.0%
Reznik Y 2023	11	36.1	8.5	14	62.7	22.9	-26.60	[-39.60; -13.60]	9.5%
Taleb N 2019	15	5.3	8.2	15	9.1	13.7	-3.80	[-11.86; 4.26]	12.7%
**Random effects model**	**413**			**301**			**-17.68**	**[-23.93; -11.44]**	**100.0%**
**Prediction interval**								**[-37.41; 2.05]**	

Heterogeneity: $I^2 = 74.6\%$, $\tau^2 = 63.0443$, $p = 0.0001$

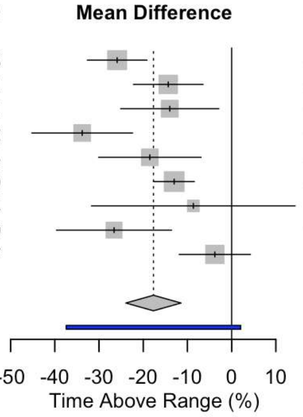

Study	Closed-loop Total	Closed-loop Mean	Closed-loop SD	Comparator Total	Comparator Mean	Comparator SD	Mean	95% CI	Weight
Borel A 2024	17	2.9	3.5	17	6.8	4.8	-3.90	[-6.71; -1.09]	41.7%
Kudva YC 2025	214	9.7	7.8	104	16.7	14.1	-7.00	[-9.90; -4.10]	41.4%
Reznik Y 2023	11	9.1	6.1	14	28.5	22.4	-19.40	[-31.65; -7.15]	16.9%
**Random effects model**	**242**			**135**			**-7.81**	**[-14.18; -1.44]**	**100.0%**
**Prediction interval**								**[-32.85; 17.23]**	

Heterogeneity: $I^2 = 72.0\%$, $\tau^2 = 23.3122$, $p = 0.0280$

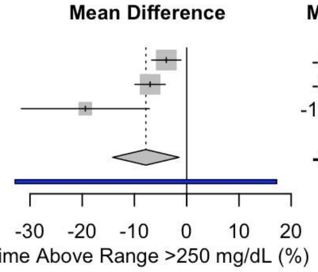

Study	Closed-loop Total	Closed-loop Mean	Closed-loop SD	Comparator Total	Comparator Mean	Comparator SD	Mean	95% CI	Weight
Bally L 2018	70	154.0	29.0	66	188.0	43.0	-34.00	[-46.40; -21.60]	14.3%
Borel A 2024	17	158.8	17.3	17	172.2	20.8	-13.40	[-26.26; -0.54]	14.1%
Boughton CK 2021	26	181.8	23.4	26	208.8	50.4	-27.00	[-48.36; -5.64]	10.6%
Daly AB 2023	26	165.6	21.6	25	226.8	54.0	-61.20	[-83.94; -38.46]	10.0%
Herzig D 2022	22	144.0	12.6	22	169.2	45.0	-25.20	[-44.73; -5.67]	11.3%
Kudva YC 2025	214	170.0	23.0	104	188.0	34.0	-18.00	[-25.22; -10.78]	16.3%
Kumareswaran K 2014	12	169.2	34.2	12	174.6	25.2	-5.40	[-29.44; 18.64]	9.6%
Taleb N 2019	15	126.0	16.2	15	133.2	21.6	-7.20	[-20.86; 6.46]	13.8%
**Random effects model**	**402**			**287**			**-23.05**	**[-34.06; -12.03]**	**100.0%**
**Prediction interval**								**[-57.46; 11.37]**	

Heterogeneity: $I^2 = 71.1\%$, $\tau^2 = 180.2801$, $p = 0.0010$

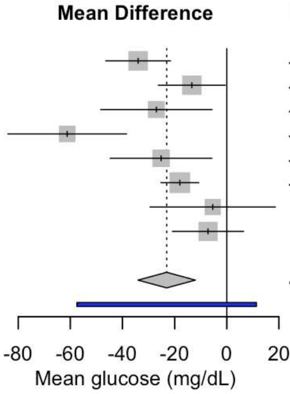

Study	Closed-loop Total	Closed-loop Mean	Closed-loop SD	Comparator Total	Comparator Mean	Comparator SD	Mean	95% CI	Weight
Bally L 2018	70	29.4	6.4	66	31.5	9.3	-2.10	[-4.80; 0.60]	14.7%
Borel A 2024	17	23.0	3.3	17	25.1	2.9	-2.10	[-4.19; -0.01]	16.8%
Boughton CK 2021	26	31.7	4.8	26	31.5	5.4	0.20	[-2.58; 2.98]	14.5%
Daly AB 2023	26	32.2	5.7	25	27.7	8.5	4.50	[0.51; 8.49]	10.8%
Herzig D 2022	22	25.4	3.9	22	28.5	7.1	-3.10	[-6.48; 0.28]	12.6%
Kudva YC 2025	214	30.0	5.0	104	29.0	5.0	1.00	[-0.17; 2.17]	19.6%
Reznik Y 2023	11	31.5	5.7	14	28.2	3.9	3.30	[-0.64; 7.24]	11.0%
**Random effects model**	**386**			**274**			**0.02**	**[-1.85; 1.90]**	**100.0%**
**Prediction interval**								**[-5.58; 5.63]**	

Heterogeneity: $I^2 = 69.6\%$, $\tau^2 = 4.3268$, $p = 0.0031$

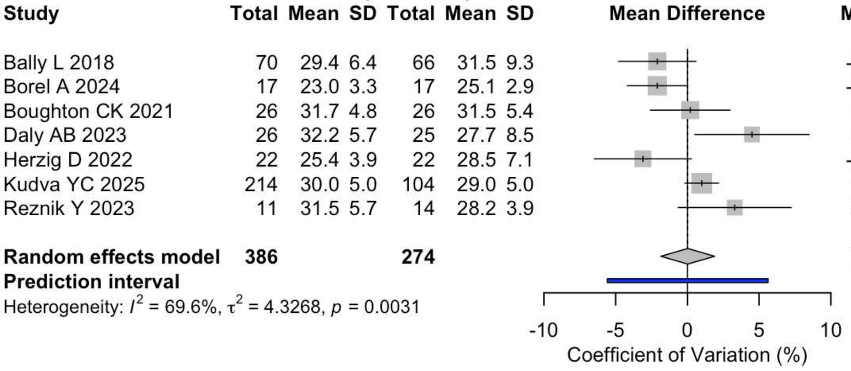

Study	Closed-loop Total	Closed-loop Mean	Closed-loop SD	Comparator Total	Comparator Mean	Comparator SD	Mean	95% CI	Weight
Bally L 2018	70	46.0	19.0	66	59.0	19.0	-13.00	[-19.39; -6.61]	18.2%
Borel A 2024	17	43.0	11.0	17	50.0	8.0	-7.00	[-13.47; -0.53]	18.1%
Boughton CK 2021	26	57.6	12.6	26	64.8	16.2	-7.20	[-15.09; 0.69]	16.0%
Daly AB 2023	26	54.0	14.4	25	61.2	18.0	-7.20	[-16.17; 1.77]	14.5%
Herzig D 2022	22	37.8	7.2	22	46.8	14.4	-9.00	[-15.73; -2.27]	17.7%
Kumareswaran K 2014	12	39.6	12.6	12	32.4	7.2	7.20	[-1.01; 15.41]	15.5%
**Random effects model**	**173**			**168**			**-6.30**	**[-11.64; -0.96]**	**100.0%**
**Prediction interval**								**[-22.05; 9.46]**	

Heterogeneity: $I^2 = 67.1\%$, $\tau^2 = 30.1501$, $p = 0.0095$

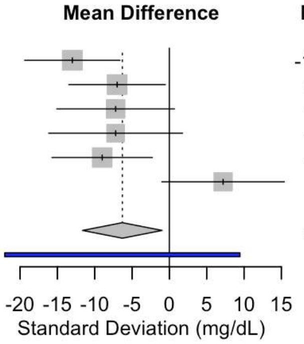

Study	Closed-loop Total	Closed-loop Mean	Closed-loop SD	Comparator Total	Comparator Mean	Comparator SD	Mean	95% CI	Weight
Daly AB 2023	26	7.3	0.8	25	8.7	1.2	-1.40	[-1.96; -0.84]	31.8%
Kudva YC 2025	209	7.3	0.9	102	7.7	1.1	-0.40	[-0.65; -0.15]	38.1%
Reznik Y 2023	11	7.4	0.6	14	8.8	1.0	-1.40	[-2.03; -0.77]	30.1%
**Random effects model**	**246**			**141**			**-1.02**	**[-1.71; -0.32]**	**100.0%**
**Prediction interval**								**[-3.87; 1.84]**	

Heterogeneity: $I^2 = 87.7\%$, $\tau^2 = 0.3145$, $p = 0.0003$

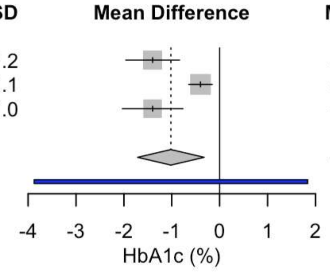

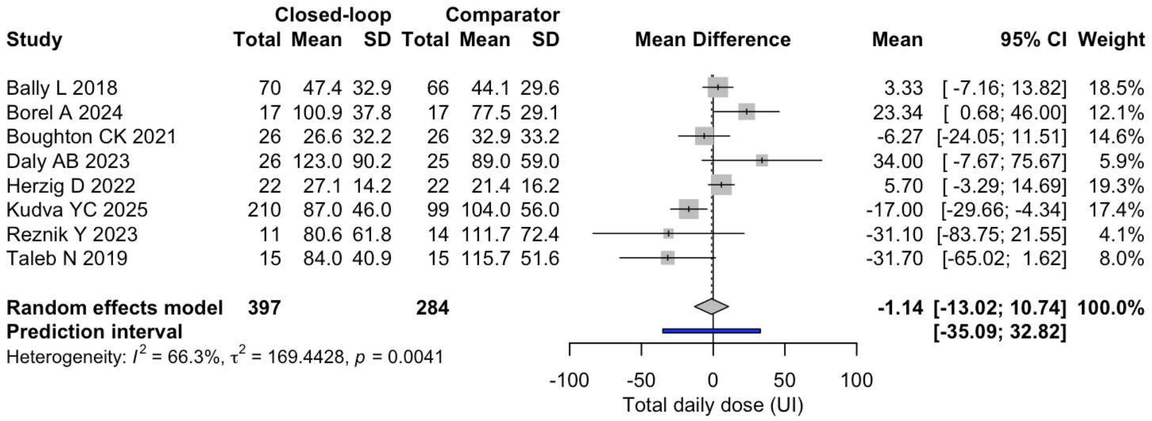
Closed-loop
Comparator
Study Total Mean SD Total Mean SD
Mean Difference
Mean 95% CI Weight
Bally L 2018 70 47.4 32.9 66 44.1 29.6 3.33 [-7.16; 13.82] 18.5%
Borel A 2024 17 100.9 37.8 17 77.5 29.1 23.34 [0.68; 46.00] 12.1%
Boughton CK 2021 26 26.6 32.2 26 32.9 33.2 -6.27 [-24.05; 11.51] 14.6%
Daly AB 2023 26 123.0 90.2 25 89.0 59.0 34.00 [-7.67; 75.67] 5.9%
Herzig D 2022 22 27.1 14.2 22 21.4 16.2 5.70 [-3.29; 14.69] 19.3%
Kudva YC 2025 210 87.0 46.0 99 104.0 56.0 -17.00 [-29.66; -4.34] 17.4%
Reznik Y 2023 11 80.6 61.8 14 111.7 72.4 -31.10 [-83.75; 21.55] 4.1%
Taleb N 2019 15 84.0 40.9 15 115.7 51.6 -31.70 [-65.02; 1.62] 8.0%
Random effects model 397 284 -1.14 [-13.02; 10.74] 100.0%
Prediction interval [-35.09; 32.82]
Heterogeneity: I2 = 66.3%, τ2 = 169.4428, p = 0.0041
-100 -50 0 50 100
Total daily dose (UI)

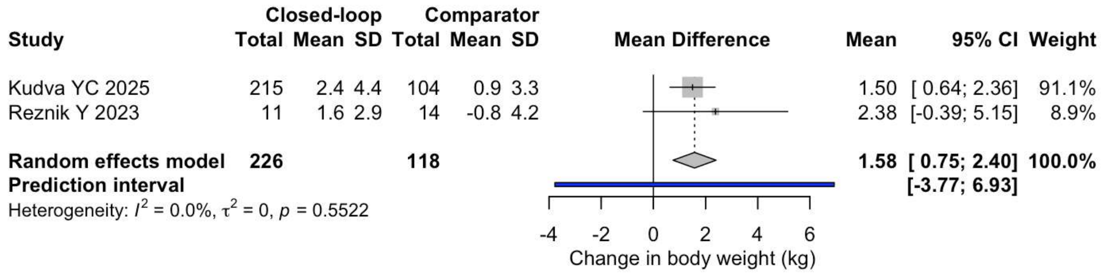
Closed-loop
Comparator
Study Total Mean SD Total Mean SD
Mean Difference
Mean 95% CI Weight
Kudva YC 2025 215 2.4 4.4 104 0.9 3.3 1.50 [0.64; 2.36] 91.1%
Reznik Y 2023 11 1.6 2.9 14 -0.8 4.2 2.38 [-0.39; 5.15] 8.9%
Random effects model 226 118 1.58 [0.75; 2.40] 100.0%
Prediction interval [-3.77; 6.93]
Heterogeneity: I2 = 0.0%, τ2 = 0, p = 0.5522
-4 -2 0 2 4 6
Change in body weight (kg)

5.2. Sensitivity analysis restricted to studies with intervention duration ≥2 weeks

Study	Closed-loop Total	Closed-loop Mean	Closed-loop SD	Comparator Total	Comparator Mean	Comparator SD	Mean	95% CI	Weight
Bally L 2018	70	75.9	28.5	66	49.9	34.4	26.00	[15.35; 36.65]	13.5%
Borel A 2024	17	76.3	12.1	17	62.0	12.1	14.33	[6.18; 22.48]	16.2%
Boughton CK 2021	26	57.1	14.3	26	42.5	24.7	14.60	[3.63; 25.57]	13.1%
Daly AB 2023	26	66.3	14.9	25	32.3	24.7	34.00	[22.75; 45.25]	12.9%
Herzig D 2022	22	84.2	8.6	22	64.3	25.4	19.90	[8.69; 31.11]	12.9%
Kudva YC 2025	214	64.0	16.0	104	52.0	21.0	12.00	[7.43; 16.57]	20.1%
Reznik Y 2023	11	63.0	9.4	14	36.6	22.0	26.40	[13.61; 39.19]	11.4%
**Random effects model**	**386**			**274**			**20.09**	**[13.95; 26.24]**	**100.0%**
**Prediction interval**								**[2.22; 37.97]**	

Heterogeneity: $I^2 = 68.5\%$, $\tau^2 = 43.5239$, $p = 0.0041$

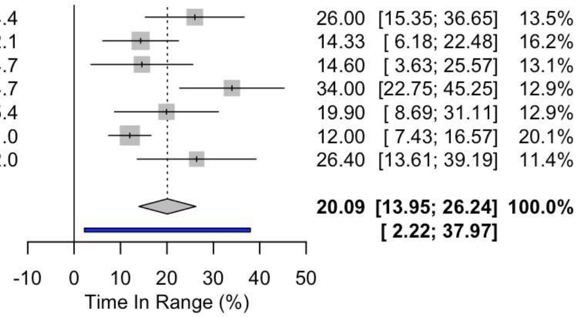

Study	Closed-loop Total	Closed-loop Mean	Closed-loop SD	Comparator Total	Comparator Mean	Comparator SD	Mean	95% CI	Weight
Bally L 2018	70	0.5	0.8	66	0.6	1.4	-0.07	[-0.45; 0.31]	4.4%
Borel A 2024	17	0.2	0.3	17	0.5	0.7	-0.33	[-0.67; 0.01]	5.4%
Boughton CK 2021	26	0.2	0.3	26	0.4	0.9	-0.24	[-0.60; 0.12]	5.0%
Daly AB 2023	26	0.5	0.5	25	0.4	0.8	0.10	[-0.28; 0.48]	4.5%
Herzig D 2022	22	0.3	0.5	22	0.2	0.5	0.07	[-0.21; 0.35]	7.9%
Kudva YC 2025	214	0.4	0.4	104	0.4	0.4	0.00	[-0.09; 0.09]	72.4%
Reznik Y 2023	11	0.8	1.5	14	0.7	1.3	0.10	[-1.02; 1.22]	0.5%
**Random effects model**	**386**			**274**			**-0.02**	**[-0.10; 0.06]**	**100.0%**
**Prediction interval**								**[-0.12; 0.08]**	

Heterogeneity: $I^2 = 0.0\%$, $\tau^2 = 0$, $p = 0.4663$

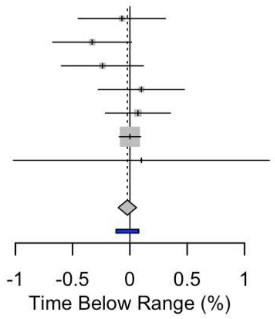

Study	Closed-loop Total	Closed-loop Mean	Closed-loop SD	Comparator Total	Comparator Mean	Comparator SD	Mean	95% CI	Weight
Bally L 2018	70	0.0	0.1	66	0.0	0.0	0.03		0.0%
Borel A 2024	17	0.0	0.0	17	0.0	0.0	0.01	[-0.01; 0.03]	38.5%
Boughton CK 2021	26	0.0	0.0	26	0.1	0.2	-0.06	[-0.13; 0.01]	15.1%
Daly AB 2023	26	0.0	0.1	25	0.1	0.2	-0.08	[-0.18; 0.02]	8.0%
Herzig D 2022	22	0.0	0.0	22	0.0	0.0	0.00		0.0%
Kudva YC 2025	214	0.1	0.1	104	0.1	0.1	0.00	[-0.02; 0.02]	37.6%
Reznik Y 2023	11	0.2	0.5	14	0.2	0.4	0.00	[-0.36; 0.36]	0.7%
**Random effects model**	**386**			**274**			**-0.01**	**[-0.04; 0.02]**	**100.0%**
**Prediction interval**								**[-0.09; 0.07]**	

Heterogeneity: $I^2 = 38.6\%$, $\tau^2 = 0.0005$, $p = 0.1642$

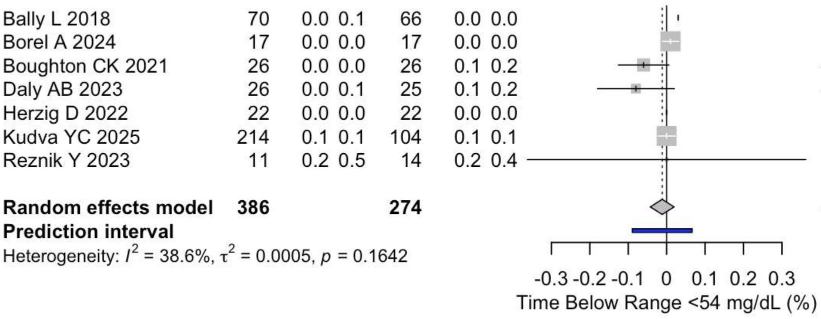

Study	Closed-loop Total	Closed-loop Mean	Closed-loop SD	Comparator Total	Comparator Mean	Comparator SD	Mean	95% CI	Weight
Bally L 2018	70	23.6	16.6	66	49.5	22.8	-25.90	[-32.64; -19.16]	17.2%
Borel A 2024	17	23.3	11.3	17	37.7	12.1	-14.34	[-22.23; -6.45]	15.9%
Boughton CK 2021	26	42.6	14.3	26	56.6	25.1	-14.00	[-25.10; -2.90]	12.4%
Daly AB 2023	26	33.2	14.8	25	67.0	25.2	-33.80	[-45.20; -22.40]	12.1%
Herzig D 2022	22	15.4	8.4	22	33.9	26.4	-18.50	[-30.08; -6.92]	12.0%
Kudva YC 2025	214	35.0	16.0	104	48.0	21.0	-13.00	[-17.57; -8.43]	19.6%
Reznik Y 2023	11	36.1	8.5	14	62.7	22.9	-26.60	[-39.60; -13.60]	10.7%
**Random effects model**	**386**			**274**			**-20.20**	**[-26.08; -14.31]**	**100.0%**
**Prediction interval**								**[-37.40; -2.99]**	

Heterogeneity: I^2 = 70.4%, τ^2 = 40.4086, p = 0.0025

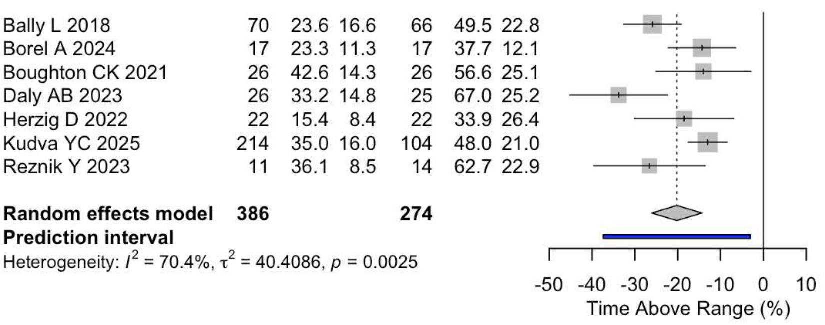

Study	Closed-loop Total	Closed-loop Mean	Closed-loop SD	Comparator Total	Comparator Mean	Comparator SD	Mean	95% CI	Weight
Borel A 2024	17	2.9	3.5	17	6.8	4.8	-3.90	[-6.71; -1.09]	41.7%
Kudva YC 2025	214	9.7	7.8	104	16.7	14.1	-7.00	[-9.90; -4.10]	41.4%
Reznik Y 2023	11	9.1	6.1	14	28.5	22.4	-19.40	[-31.65; -7.15]	16.9%
**Random effects model**	**242**			**135**			**-7.81**	**[-14.18; -1.44]**	**100.0%**
**Prediction interval**								**[-32.85; 17.23]**	

Heterogeneity: I^2 = 72.0%, τ^2 = 23.3122, p = 0.0280

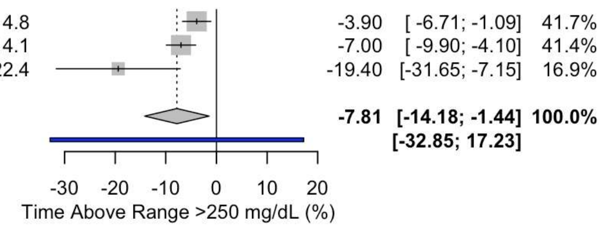

Study	Closed-loop Total	Closed-loop Mean	Closed-loop SD	Comparator Total	Comparator Mean	Comparator SD	Mean	95% CI	Weight
Bally L 2018	70	154.0	29.0	66	188.0	43.0	-34.00	[-46.40; -21.60]	18.8%
Borel A 2024	17	158.8	17.3	17	172.2	20.8	-13.40	[-26.26; -0.54]	18.5%
Boughton CK 2021	26	181.8	23.4	26	208.8	50.4	-27.00	[-48.36; -5.64]	13.6%
Daly AB 2023	26	165.6	21.6	25	226.8	54.0	-61.20	[-83.94; -38.46]	12.8%
Herzig D 2022	22	144.0	12.6	22	169.2	45.0	-25.20	[-44.73; -5.67]	14.6%
Kudva YC 2025	214	170.0	23.0	104	188.0	34.0	-18.00	[-25.22; -10.78]	21.6%
**Random effects model**	**375**			**260**			**-27.98**	**[-40.10; -15.85]**	**100.0%**
**Prediction interval**								**[-64.47; 8.52]**	

Heterogeneity: I^2 = 72.3%, τ^2 = 163.2922, p = 0.0029

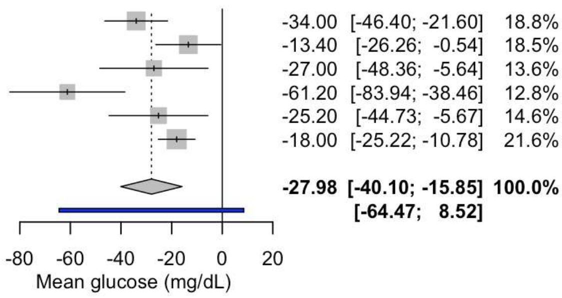

Study	Closed-loop Total	Closed-loop Mean	Closed-loop SD	Comparator Total	Comparator Mean	Comparator SD	Mean Difference	95% CI	Weight
Bally L 2018	70	29.4	6.4	66	31.5	9.3	-2.10	[-4.80; 0.60]	14.7%
Borel A 2024	17	23.0	3.3	17	25.1	2.9	-2.10	[-4.19; -0.01]	16.8%
Boughton CK 2021	26	31.7	4.8	26	31.5	5.4	0.20	[-2.58; 2.98]	14.5%
Daly AB 2023	26	32.2	5.7	25	27.7	8.5	4.50	[0.51; 8.49]	10.8%
Herzig D 2022	22	25.4	3.9	22	28.5	7.1	-3.10	[-6.48; 0.28]	12.6%
Kudva YC 2025	214	30.0	5.0	104	29.0	5.0	1.00	[-0.17; 2.17]	19.6%
Reznik Y 2023	11	31.5	5.7	14	28.2	3.9	3.30	[-0.64; 7.24]	11.0%
**Random effects model**	**386**			**274**			**0.02**	**[-1.85; 1.90]**	**100.0%**
**Prediction interval**								**[-5.58; 5.63]**	

Heterogeneity: $I^2 = 69.6\%$, $\tau^2 = 4.3268$, $p = 0.0031$

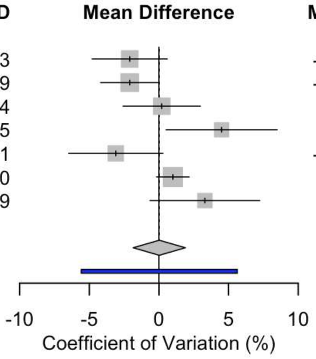

Study	Closed-loop Total	Closed-loop Mean	Closed-loop SD	Comparator Total	Comparator Mean	Comparator SD	Mean Difference	95% CI	Weight
Bally L 2018	70	46.0	19.0	66	59.0	19.0	-13.00	[-19.39; -6.61]	24.7%
Borel A 2024	17	43.0	11.0	17	50.0	8.0	-7.00	[-13.47; -0.53]	24.2%
Boughton CK 2021	26	57.6	12.6	26	64.8	16.2	-7.20	[-15.09; 0.69]	16.2%
Daly AB 2023	26	54.0	14.4	25	61.2	18.0	-7.20	[-16.17; 1.77]	12.6%
Herzig D 2022	22	37.8	7.2	22	46.8	14.4	-9.00	[-15.73; -2.27]	22.3%
**Random effects model**	**161**			**156**			**-8.99**	**[-12.17; -5.81]**	**100.0%**
**Prediction interval**								**[-13.49; -4.49]**	

Heterogeneity: $I^2 = 0.0\%$, $\tau^2 = 0$, $p = 0.6939$

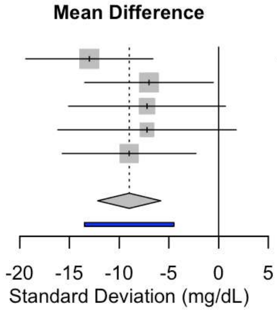

Study	Closed-loop Total	Closed-loop Mean	Closed-loop SD	Comparator Total	Comparator Mean	Comparator SD	Mean Difference	95% CI	Weight
Daly AB 2023	26	7.3	0.8	25	8.7	1.2	-1.40	[-1.96; -0.84]	31.8%
Kudva YC 2025	209	7.3	0.9	102	7.7	1.1	-0.40	[-0.65; -0.15]	38.1%
Reznik Y 2023	11	7.4	0.6	14	8.8	1.0	-1.40	[-2.03; -0.77]	30.1%
**Random effects model**	**246**			**141**			**-1.02**	**[-1.71; -0.32]**	**100.0%**
**Prediction interval**								**[-3.87; 1.84]**	

Heterogeneity: $I^2 = 87.7\%$, $\tau^2 = 0.3145$, $p = 0.0003$

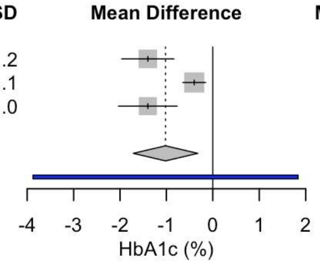

Study	Closed-loop Total	Closed-loop Mean	Closed-loop SD	Comparator Total	Comparator Mean	Comparator SD	Mean	95% CI	Weight
Bally L 2018	70	47.4	32.9	66	44.1	29.6	3.33	[-7.16; 13.82]	20.8%
Borel A 2024	17	100.9	37.8	17	77.5	29.1	23.34	[0.68; 46.00]	12.7%
Boughton CK 2021	26	26.6	32.2	26	32.9	33.2	-6.27	[-24.05; 11.51]	15.7%
Daly AB 2023	26	123.0	90.2	25	89.0	59.0	34.00	[-7.67; 75.67]	5.8%
Herzig D 2022	22	27.1	14.2	22	21.4	16.2	5.70	[-3.29; 14.69]	21.9%
Kudva YC 2025	210	87.0	46.0	99	104.0	56.0	-17.00	[-29.66; -4.34]	19.3%
Reznik Y 2023	11	80.6	61.8	14	111.7	72.4	-31.10	[-83.75; 21.55]	4.0%
**Random effects model**	**382**			**269**			**1.37**	**[-10.03; 12.77]**	**100.0%**
**Prediction interval**								**[-30.31; 33.05]**	

Heterogeneity: $I^2 = 65.1\%$, $\tau^2 = 133.8017$, $p = 0.0085$

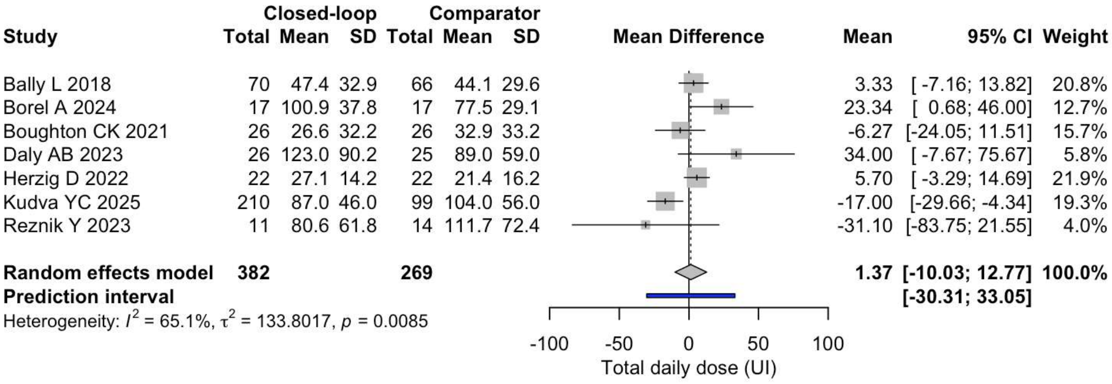

Study	Closed-loop Total	Closed-loop Mean	Closed-loop SD	Comparator Total	Comparator Mean	Comparator SD	Mean	95% CI	Weight
Kudva YC 2025	215	2.4	4.4	104	0.9	3.3	1.50	[0.64; 2.36]	91.1%
Reznik Y 2023	11	1.6	2.9	14	-0.8	4.2	2.38	[-0.39; 5.15]	8.9%
**Random effects model**	**226**			**118**			**1.58**	**[0.75; 2.40]**	**100.0%**
**Prediction interval**								**[-3.77; 6.93]**	

Heterogeneity: $I^2 = 0.0\%$, $\tau^2 = 0$, $p = 0.5522$

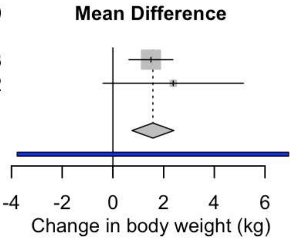

5.3. Sensitivity analysis restricted to studies with low risk of bias

Study	Closed-loop Total	Closed-loop Mean	Closed-loop SD	Comparator Total	Comparator Mean	Comparator SD	Mean	95% CI	Weight
Borel A 2024	17	76.3	12.1	17	62.0	12.1	14.33	[6.18; 22.48]	21.4%
Boughton CK 2021	26	57.1	14.3	26	42.5	24.7	14.60	[3.63; 25.57]	17.7%
Daly AB 2023	26	66.3	14.9	25	32.3	24.7	34.00	[22.75; 45.25]	17.4%
Herzig D 2022	22	84.2	8.6	22	64.3	25.4	19.90	[8.69; 31.11]	17.4%
Kudva YC 2025	214	64.0	16.0	104	52.0	21.0	12.00	[7.43; 16.57]	26.1%
**Random effects model**	**305**			**194**			**18.15**	**[10.76; 25.55]**	**100.0%**
**Prediction interval**								**[-3.94; 40.24]**	

Heterogeneity: $I^2 = 70.0\%$, $\tau^2 = 49.0642$, $p = 0.0098$

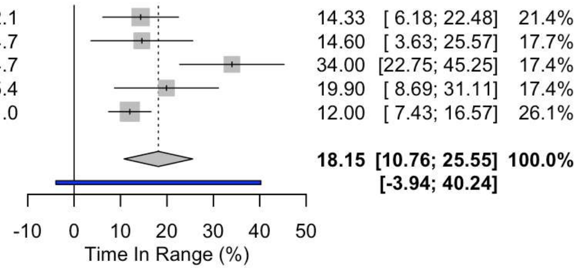

Study	Closed-loop Total	Closed-loop Mean	Closed-loop SD	Comparator Total	Comparator Mean	Comparator SD	Mean	95% CI	Weight
Borel A 2024	17	0.2	0.3	17	0.5	0.7	-0.33	[-0.67; 0.01]	8.7%
Boughton CK 2021	26	0.2	0.3	26	0.4	0.9	-0.24	[-0.60; 0.12]	8.2%
Daly AB 2023	26	0.5	0.5	25	0.4	0.8	0.10	[-0.28; 0.48]	7.4%
Herzig D 2022	22	0.3	0.5	22	0.2	0.5	0.07	[-0.21; 0.35]	12.5%
Kudva YC 2025	214	0.4	0.4	104	0.4	0.4	0.00	[-0.09; 0.09]	63.2%
**Random effects model**	**305**			**194**			**-0.03**	**[-0.14; 0.07]**	**100.0%**
**Prediction interval**								**[-0.23; 0.17]**	

Heterogeneity: $I^2 = 27.5\%$, $\tau^2 = 0.0023$, $p = 0.2381$

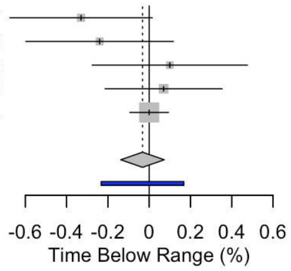

Study	Closed-loop Total	Closed-loop Mean	Closed-loop SD	Comparator Total	Comparator Mean	Comparator SD	Mean	95% CI	Weight
Boughton CK 2021	26	0.0	0.0	26	0.1	0.2	-0.06	[-0.13; 0.01]	29.9%
Daly AB 2023	26	0.0	0.1	25	0.1	0.2	-0.08	[-0.18; 0.02]	18.4%
Herzig D 2022	22	0.0	0.0	22	0.0	0.0	0.00		0.0%
Kudva YC 2025	214	0.1	0.1	104	0.1	0.1	0.00	[-0.02; 0.02]	51.7%
**Random effects model**	**288**			**177**			**-0.03**	**[-0.08; 0.02]**	**100.0%**
**Prediction interval**								**[-0.22; 0.16]**	

Heterogeneity: $I^2 = 58.8\%$, $\tau^2 = 0.0012$, $p = 0.0884$

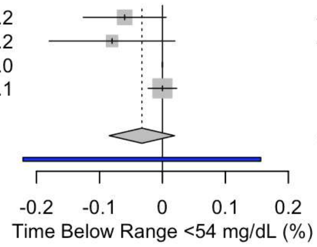

Study	Closed-loop Total	Closed-loop Mean	Closed-loop SD	Comparator Total	Comparator Mean	Comparator SD	Mean	95% CI	Weight
Borel A 2024	17	23.3	11.3	17	37.7	12.1	-14.34	[-22.23; -6.45]	22.1%
Boughton CK 2021	26	42.6	14.3	26	56.6	25.1	-14.00	[-25.10; -2.90]	17.3%
Daly AB 2023	26	33.2	14.8	25	67.0	25.2	-33.80	[-45.20; -22.40]	16.9%
Herzig D 2022	22	15.4	8.4	22	33.9	26.4	-18.50	[-30.08; -6.92]	16.7%
Kudva YC 2025	214	35.0	16.0	104	48.0	21.0	-13.00	[-17.57; -8.43]	27.1%
**Random effects model**	**305**			**194**			**-17.90**	**[-24.88; -10.91]**	**100.0%**
**Prediction interval**								**[-38.32; 2.52]**	

Heterogeneity: $I^2 = 65.1\%$, $\tau^2 = 41.3856$, $p = 0.0219$

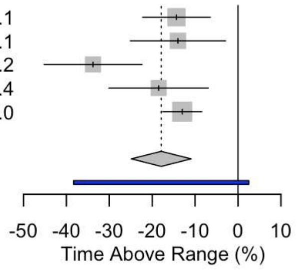

Study	Closed-loop Total	Closed-loop Mean	Closed-loop SD	Comparator Total	Comparator Mean	Comparator SD	Mean	95% CI	Weight
Borel A 2024	17	2.9	3.5	17	6.8	4.8	-3.90	[-6.71; -1.09]	50.8%
Kudva YC 2025	214	9.7	7.8	104	16.7	14.1	-7.00	[-9.90; -4.10]	49.2%
**Random effects model**	**231**			**121**			**-5.43**	**[-8.46; -2.39]**	**100.0%**
**Prediction interval**								**[-34.08; 23.22]**	

Heterogeneity: $I^2 = 55.8\%$, $\tau^2 = 2.6816$, $p = 0.1325$

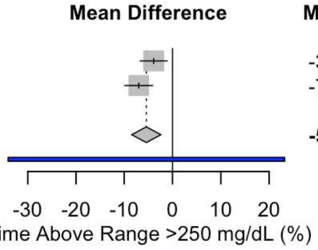

Study	Closed-loop Total	Closed-loop Mean	Closed-loop SD	Comparator Total	Comparator Mean	Comparator SD	Mean	95% CI	Weight
Boughton CK 2021	26	181.8	23.4	26	208.8	50.4	-27.00	[-48.36; -5.64]	22.7%
Daly AB 2023	26	165.6	21.6	25	226.8	54.0	-61.20	[-83.94; -38.46]	21.8%
Herzig D 2022	22	144.0	12.6	22	169.2	45.0	-25.20	[-44.73; -5.67]	23.9%
Kudva YC 2025	214	170.0	23.0	104	188.0	34.0	-18.00	[-25.22; -10.78]	31.6%
**Random effects model**	**288**			**177**			**-31.17**	**[-49.20; -13.14]**	**100.0%**
**Prediction interval**								**[-89.77; 27.43]**	

Heterogeneity: $I^2 = 76.7\%$, $\tau^2 = 254.3607$, $p = 0.0050$

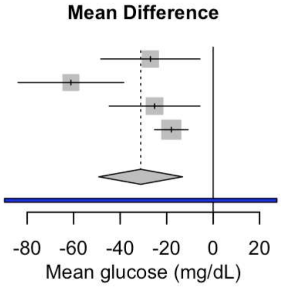

Study	Closed-loop Total	Closed-loop Mean	Closed-loop SD	Comparator Total	Comparator Mean	Comparator SD	Mean	95% CI	Weight
Borel A 2024	17	23.0	3.3	17	25.1	2.9	-2.10	[-4.19; -0.01]	22.6%
Boughton CK 2021	26	31.7	4.8	26	31.5	5.4	0.20	[-2.58; 2.98]	19.5%
Daly AB 2023	26	32.2	5.7	25	27.7	8.5	4.50	[0.51; 8.49]	14.7%
Herzig D 2022	22	25.4	3.9	22	28.5	7.1	-3.10	[-6.48; 0.28]	17.0%
Kudva YC 2025	214	30.0	5.0	104	29.0	5.0	1.00	[-0.17; 2.17]	26.2%
**Random effects model**	**305**			**194**			**-0.04**	**[-2.25; 2.18]**	**100.0%**
**Prediction interval**								**[-6.72; 6.65]**	

Heterogeneity: $I^2 = 72.5\%$, $\tau^2 = 4.5212$, $p = 0.0057$

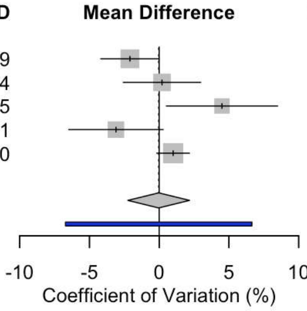

Study	Closed-loop Total	Closed-loop Mean	Closed-loop SD	Comparator Total	Comparator Mean	Comparator SD	Mean	95% CI	Weight
Borel A 2024	17	43.0	11.0	17	50.0	8.0	-7.00	[-13.47; -0.53]	32.1%
Boughton CK 2021	26	57.6	12.6	26	64.8	16.2	-7.20	[-15.09; 0.69]	21.6%
Daly AB 2023	26	54.0	14.4	25	61.2	18.0	-7.20	[-16.17; 1.77]	16.7%
Herzig D 2022	22	37.8	7.2	22	46.8	14.4	-9.00	[-15.73; -2.27]	29.6%
**Random effects model**	**91**			**90**			**-7.67**	**[-11.33; -4.01]**	**100.0%**
**Prediction interval**								**[-13.62; -1.72]**	

Heterogeneity: $I^2 = 0.0\%$, $\tau^2 = 0$, $p = 0.9750$

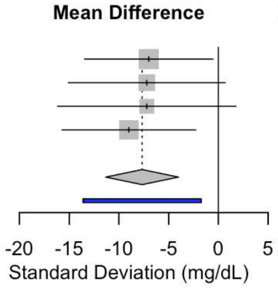

Study	Closed-loop Total	Closed-loop Mean	Closed-loop SD	Comparator Total	Comparator Mean	Comparator SD	Mean	95% CI	Weight
Daly AB 2023	26	7.3	0.8	25	8.7	1.2	-1.40	[-1.96; -0.84]	46.7%
Kudva YC 2025	209	7.3	0.9	102	7.7	1.1	-0.40	[-0.65; -0.15]	53.3%
**Random effects model**	**235**			**127**			**-0.87**	**[-1.84; 0.11]**	**100.0%**
**Prediction interval**								**[-11.50; 9.76]**	

Heterogeneity: $I^2 = 90.2\%$, $\tau^2 = 0.4510$, $p = 0.0014$

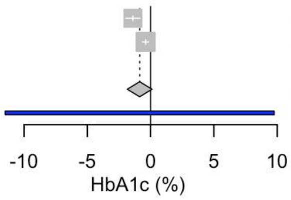

Study	Closed-loop Total	Closed-loop Mean	Closed-loop SD	Comparator Total	Comparator Mean	Comparator SD	Mean	95% CI	Weight
Borel A 2024	17	100.9	37.8	17	77.5	29.1	23.34	[0.68; 46.00]	36.9%
Boughton CK 2021	26	26.6	32.2	26	32.9	33.2	-6.27	[-24.05; 11.51]	42.0%
Daly AB 2023	26	123.0	90.2	25	89.0	59.0	34.00	[-7.67; 75.67]	21.0%
**Random effects model**	**69**			**68**			**13.13**	**[-11.32; 37.58]**	**100.0%**
**Prediction interval**								**[-77.47; 103.74]**	

Heterogeneity: $I^2 = 65.1\%$, $\tau^2 = 287.8445$, $p = 0.0570$

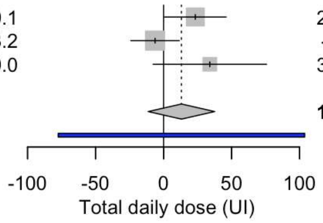

Study	Closed-loop Total	Closed-loop Mean	Closed-loop SD	Comparator Total	Comparator Mean	Comparator SD	Mean	95% CI	Weight
Kudva YC 2025	215	2.4	4.4	104	0.9	3.3	1.50	[0.64; 2.36]	91.1%
Reznik Y 2023	11	1.6	2.9	14	-0.8	4.2	2.38	[-0.39; 5.15]	8.9%
**Random effects model**	**226**			**118**			**1.58**	**[0.75; 2.40]**	**100.0%**
**Prediction interval**								**[-3.77; 6.93]**	

Heterogeneity: $I^2 = 0.0\%$, $\tau^2 = 0$, $p = 0.5522$

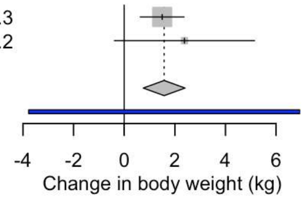

5.4. Sensitivity analysis restricted to studies with no imputed standard deviations

Study	Closed-loop Total	Closed-loop Mean	Closed-loop SD	Comparator Total	Comparator Mean	Comparator SD	Mean	95% CI	Weight
Borel A 2024	17	76.3	12.1	17	62.0	12.1	14.33	[6.18; 22.48]	14.8%
Boughton CK 2021	26	57.1	14.3	26	42.5	24.7	14.60	[3.63; 25.57]	12.4%
Daly AB 2023	26	66.3	14.9	25	32.3	24.7	34.00	[22.75; 45.25]	12.2%
Herzig D 2022	22	84.2	8.6	22	64.3	25.4	19.90	[8.69; 31.11]	12.2%
Kudva YC 2025	214	64.0	16.0	104	52.0	21.0	12.00	[7.43; 16.57]	17.7%
Kumareswaran K 2014	12	44.7	28.5	12	23.0	34.4	21.67	[-3.60; 46.94]	4.8%
Reznik Y 2023	11	63.0	9.4	14	36.6	22.0	26.40	[13.61; 39.19]	11.0%
Taleb N 2019	15	90.4	8.1	15	84.2	13.2	6.20	[-1.64; 14.04]	15.0%
**Random effects model**	**343**			**235**			**17.48**	**[11.06; 23.89]**	**100.0%**
**Prediction interval**								**[-1.73; 36.68]**	

Heterogeneity: $I^2 = 67.5\%$, $\tau^2 = 55.2670$, $p = 0.0031$

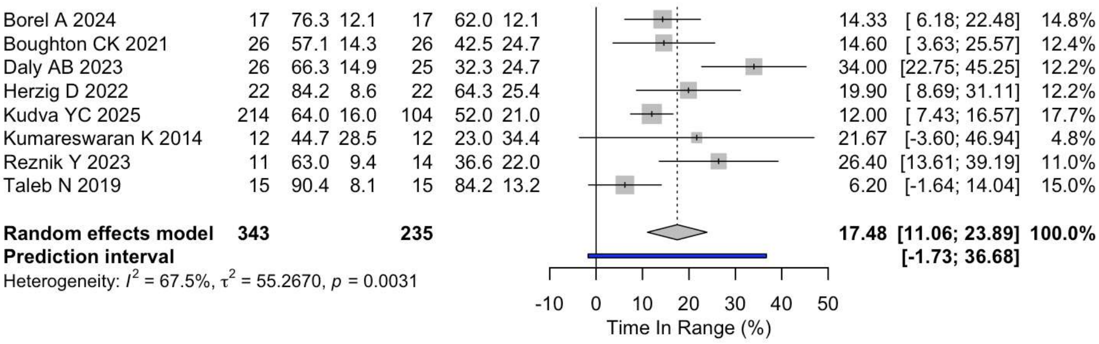

5.5. Sensitivity analysis restricted to studies comparing closed-loop insulin delivery with other types of insulin therapy

Study	Closed-loop Total	Closed-loop Mean	Closed-loop SD	Comparator Total	Comparator Mean	Comparator SD	Mean	95% CI	Weight
Bally L 2018	70	75.9	28.5	66	49.9	34.4	26.00	[15.35; 36.65]	11.8%
Borel A 2024	17	76.3	12.1	17	62.0	12.1	14.33	[6.18; 22.48]	13.7%
Boughton CK 2021	26	57.1	14.3	26	42.5	24.7	14.60	[3.63; 25.57]	11.5%
Daly AB 2023	26	66.3	14.9	25	32.3	24.7	34.00	[22.75; 45.25]	11.3%
Herzig D 2022	22	84.2	8.6	22	64.3	25.4	19.90	[8.69; 31.11]	11.4%
Kudva YC 2025	214	64.0	16.0	104	52.0	21.0	12.00	[7.43; 16.57]	16.2%
Reznik Y 2023	11	63.0	9.4	14	36.6	22.0	26.40	[13.61; 39.19]	10.3%
Taleb N 2019	15	90.4	8.1	15	84.2	13.2	6.20	[-1.64; 14.04]	13.9%
**Random effects model**	**401**			**289**			**18.33**	**[12.01; 24.64]**	**100.0%**
**Prediction interval**								**[-1.33; 37.98]**	

Heterogeneity: $I^2 = 72.3\%$, $\tau^2 = 58.7159$, $p = 0.0007$

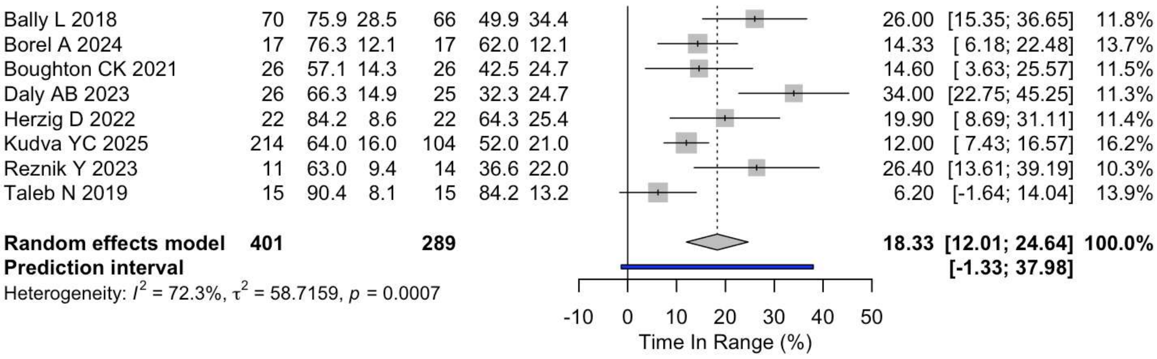

Study	Closed-loop Total	Closed-loop Mean	Closed-loop SD	Comparator Total	Comparator Mean	Comparator SD	Mean	95% CI	Weight
Bally L 2018	70	0.5	0.8	66	0.6	1.4	-0.07	[-0.45; 0.31]	5.0%
Borel A 2024	17	0.2	0.3	17	0.5	0.7	-0.33	[-0.67; 0.01]	6.1%
Boughton CK 2021	26	0.2	0.3	26	0.4	0.9	-0.24	[-0.60; 0.12]	5.7%
Daly AB 2023	26	0.5	0.5	25	0.4	0.8	0.10	[-0.28; 0.48]	5.1%
Herzig D 2022	22	0.3	0.5	22	0.2	0.5	0.07	[-0.21; 0.35]	8.9%
Kudva YC 2025	214	0.4	0.4	104	0.4	0.4	0.00	[-0.09; 0.09]	68.4%
Reznik Y 2023	11	0.8	1.5	14	0.7	1.3	0.10	[-1.02; 1.22]	0.6%
Taleb N 2019	15	0.2	0.6	15	1.4	3.5	-1.20	[-3.00; 0.60]	0.2%
**Random effects model**	**401**			**289**			**-0.03**	**[-0.11; 0.06]**	**100.0%**
**Prediction interval**								**[-0.14; 0.09]**	

Heterogeneity: $I^2 = 3.6\%$, $\tau^2 = 0.0005$, $p = 0.4023$

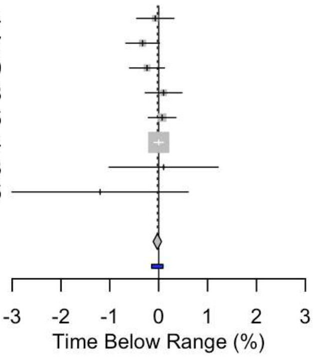

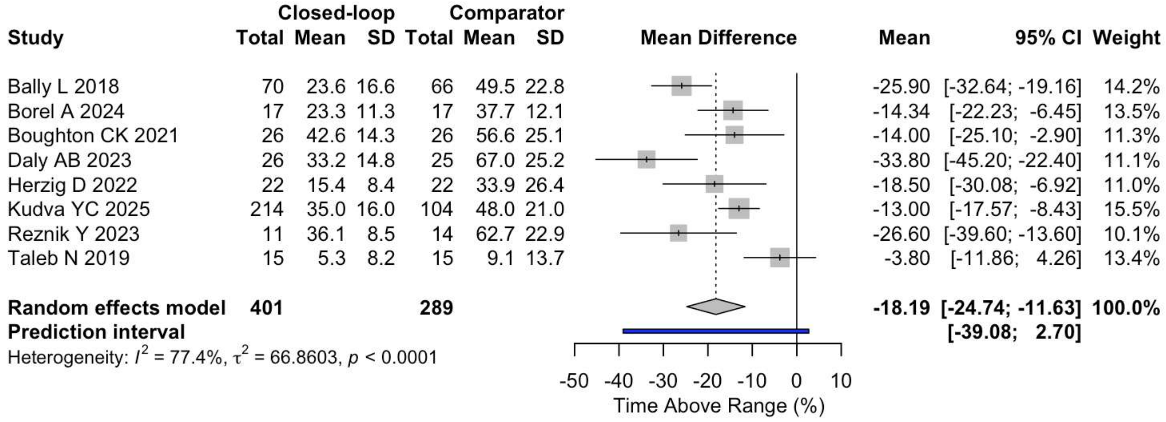

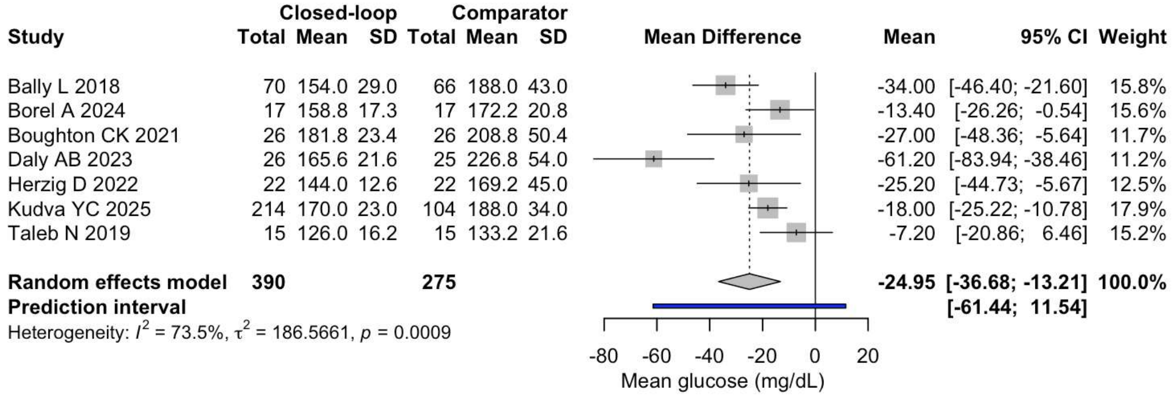

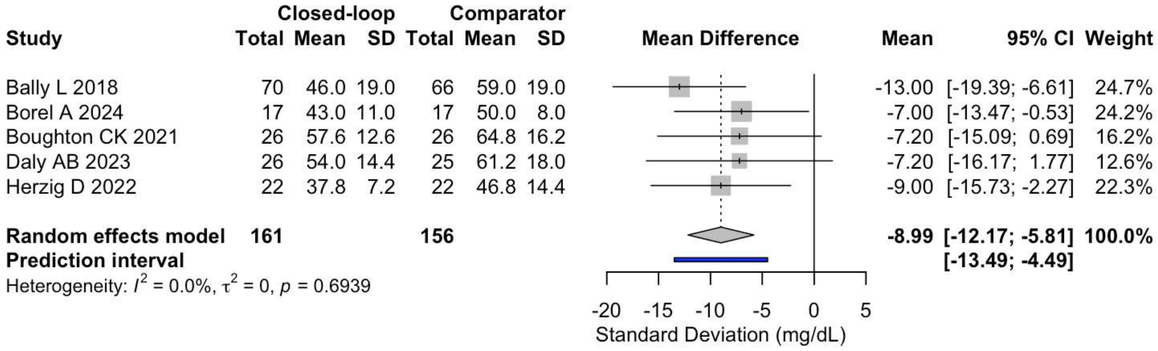

Each forest plot displays the pooled mean difference with corresponding 95% confidence intervals for each study, based on random-effects meta-analyses. The overall pooled estimate and its prediction interval are reported at the bottom of each plot. Measures of heterogeneity are also provided when applicable. SD, standard deviation, CI, confidence interval.

The sensitivity analysis restricted to studies with no imputed standard deviations was conducted only for the Time In Range (TIR) outcome. For all other endpoints, no standard deviations had to be imputed; therefore, the results of the sensitivity analysis coincided with the overall analysis and are not reported separately.

The sensitivity analysis restricted to studies comparing closed-loop insulin delivery (i.e., excluding the study by Kumareswaran K et al.) with other types of insulin therapy was performed only for the Time in Range (TIR), Time Below Range (TBR), Time Above Range (TAR), mean glucose, and standard deviation (SD) outcomes. For all other endpoints, data were not reported in the study by Kumareswaran K et al.; therefore, the sensitivity analysis coincided with the overall analysis and is not reported separately.

Supplement 6. Summary of findings

**Automated insulin delivery (AID) in people with type 2 diabetes**				
**Patients or population:**people with type 2 diabetes treated with insulin injections<br>**Intervention:** closed loop insulin delivery<br>**Comparison:** any antidiabetes medication<br>**Outcomes:** time spent in the 70-180 mg/dL glucose range (TIR), time spent below range (TBR), time spent above range (TAR), measures of glucose variability, hemoglobin A1c (HbA1c), body weight and body mass index (BMI), total daily insulin dose, patients' satisfaction and quality of life, occurrence of confirmed and severe hypoglycemia, occurrence of diabetic ketoacidosis (DKA), cardiovascular outcomes<br>**Setting:** patients with type 2 diabetes enrolled in randomized controlled trial				
**Outcome**	**No of studies/ patients**	**Mean Difference [95% CI]**	**Certainty of evidence (GRADE)**	**Comment**
**TIR (%)**	9/714	18.43 [12.4 to 24.46]	⊕⊕○○ **Low**[a,b] due to indirectness and heterogeneity	AID may increase TIR more than any other antidiabetes medication
**TBR (%)**	9/714	-3 [-0.11 to 0.06]	⊕⊕⊕○ **Moderate**[b] due to indirectness	AID and any other antidiabetes medication probably have a similar efficacy on TBR reduction
**TAR (%)**	9/714	-17.68 [-23.9 to -11.44]	⊕⊕○○ **Low**[b,c] due to indirectness and inconsistency	AID may reduce TAR more than any other antidiabetes medication
**SD (mg/dL)**	6/341	-6.3 [-11.6 to -0.69]	⊕⊕⊕○ **Moderate**[d] due to inconsistency	AID probably reduce SD more than any other antidiabetes medication
**HbA1c (%)**	3/387	-1.02 [-1.71 to -0.32]	⊕⊕⊕○ **Moderate**[e] due to inconsistency	AID probably reduce HbA1c more than any other antidiabetes medication
**Total daily insulin dose (TDD)**	8/681	-1.14 [-13.02 to 10.74]	⊕⊕○○ **Low**[b,f] due to indirectness and inconsistency	AID and any other antidiabetes medication may have a similar effect on insulin TDD
**Body weight (kg)**	2/344	1.58 [0.75 to 2.4]	⊕⊕⊕⊕ **High**	AID increases body weight more than any other antidiabetes medication
[a]Between-study heterogeneity is substantial ($I^2 = 68.6\%$, $\tau^2 = 54.9$). The 95% prediction interval crosses the null (−0.07 to 36.92), indicating that a future similar study could find no effect. Heterogeneity is				

plausibly due to clinical/methodological differences (inpatient vs outpatient, dialysis vs general T2D, follow-up from 24 h to 8–13 weeks, hybrid vs fully closed-loop, and comparator MDI vs CSII). Direction of effect is consistently in favour of closed-loop and the largest study shows benefit, hence one level downgrade was applied.

[b]Indirectness: Approximately half of the pooled evidence comes from inpatient, dialysis, or 24-hour studies, which differ from our target population (adults with T2D in ambulatory care over several weeks). Although the direction of effect is consistent, these setting/duration differences may modify the magnitude of benefit. Therefore, one level downgrade was applied.

[c]Inconsistency: downgraded one level (I^2 = 74.6%, τ^2 high; prediction interval −37.41 to 2.05 crosses no effect), likely due to differences in setting (inpatient/dialysis vs ambulatory), duration (24 h to 13 weeks), and system type.

[d]Inconsistency: downgraded one level (I^2 = 87.7%; prediction interval −3.87% to +1.84%); heterogeneity likely due to trial duration vs HbA1c kinetics (8 vs 12–13 weeks), crossover design (possible carryover), baseline HbA1c and comparator differences.

[e]Inconsistency: downgraded one level (I^2 = 87.7%; prediction interval −3.87% to +1.84%); heterogeneity likely due to trial duration vs HbA1c kinetics (8 vs 12–13 weeks), crossover design (possible carryover), baseline HbA1c and comparator differences.

[f]Inconsistency: downgraded one level (I^2 = 66%, prediction interval −35 to +33 U/day) with effects in both directions across settings/systems.

Supplement 7. Subgroup analysis, fully closed-loop vs. hybrid closed-loop

Study	Closed-loop Total	Closed-loop Mean	Closed-loop SD	Comparator Total	Comparator Mean	Comparator SD	Mean	95% CI	Weight
**Type of Closed-Loop: Fully**									
Bally L 2018	70	75.9	28.5	66	49.9	34.4	26.00	[15.35; 36.65]	11.2%
Boughton CK 2021	26	57.1	14.3	26	42.5	24.7	14.60	[3.63; 25.57]	11.0%
Daly AB 2023	26	66.3	14.9	25	32.3	24.7	34.00	[22.75; 45.25]	10.8%
Herzig D 2022	22	84.2	8.6	22	64.3	25.4	19.90	[8.69; 31.11]	10.8%
Kumareswaran K 2014	12	44.7	28.5	12	23.0	34.4	21.67	[-3.60; 46.94]	4.3%
**Random effects model**	**156**			**151**			**23.46**	**[16.09; 30.83]**	**48.1%**
Heterogeneity: I^2 = 38.5%, τ^2 = 29.3888, p = 0.1646									
**Type of Closed-Loop: Hybrid**									
Borel A 2024	17	76.3	12.1	17	62.0	12.1	14.33	[6.18; 22.48]	13.1%
Kudva YC 2025	214	64.0	16.0	104	52.0	21.0	12.00	[7.43; 16.57]	15.7%
Reznik Y 2023	11	63.0	9.4	14	36.6	22.0	26.40	[13.61; 39.19]	9.7%
Taleb N 2019	15	90.4	8.1	15	84.2	13.2	6.20	[-1.64; 14.04]	13.4%
**Random effects model**	**257**			**150**			**13.32**	**[6.99; 19.66]**	**51.9%**
Heterogeneity: I^2 = 58.6%, τ^2 = 25.0219, p = 0.0644									
**Random effects model**	**413**			**301**			**18.43**	**[12.40; 24.46]**	**100.0%**
**Prediction interval**								**[-0.07; 36.92]**	

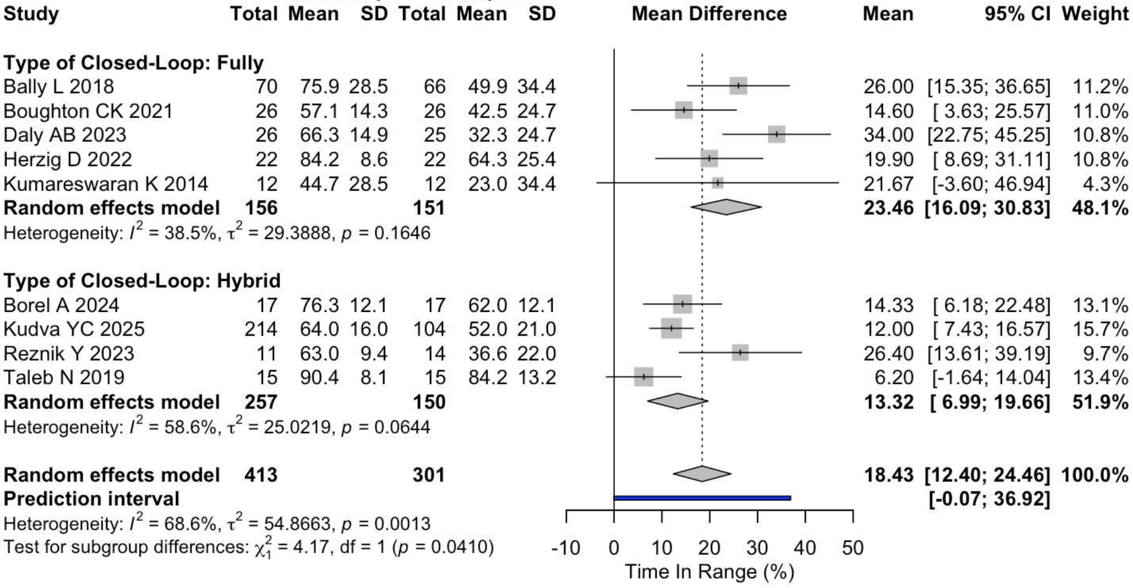

Heterogeneity: I^2 = 68.6%, τ^2 = 54.8663, p = 0.0013

Test for subgroup differences: χ^2_1 = 4.17, df = 1 (p = 0.0410)

CREDIBILITY ASSESSMENT - ICEMAN

Essential preliminary considerations to define the possible effect modification of interest

State a single candidate effect modifier (e.g., age or comorbidity): fully closed-loop vs. hybrid closed-loop

Was the effect modifier measured before or at randomization? [x] yes, continue [ ] no, stop here and refer to manual for further instructions

State a single outcome and time-point (e.g., mortality at 1 year follow-up): TIR (%)

State a single effect measure (e.g., relative risk or risk difference): mean difference

1: Is the analysis of effect modification based on comparison within rather than between trials?

[x] Completely between	[ ] Mostly between or unclear	[ ] Mostly within	[ ] Completely within
Subgroup analysis or meta-regression comparing overall effects of each individual trial. This is typical for aggregate data meta-analysis.	*Subgroup analysis or meta-regression with most information coming from overall effects, but some trials providing within-trial subgroup information*	*Most trials providing within-trial subgroup information; or individual participant data analysis that combines within and between trial information*	*All trials providing within-trial subgroup information or individual participant data; and the analysis separates within from between trial information, e.g., meta-analysis of interactions*

Comment:

2: For within-trial comparisons, is the effect modification similar from trial to trial? [ ] Not applicable: no or one within-RCT comparison

[ ] Definitely not similar	[ ] Probably not similar or unclear	[ ] Mostly similar	[ ] Definitely similar
Effect modification reported for two or more trials and clearly different directions	*Effect modification not reported for individual trials or too imprecise to tell*	*Effect modification reported for two or more trials, mostly similar in direction, but considerable differences in magnitude*	*Effect modification reported for two or more trials, similar in direction, only some differences in magnitude*

Comment:

3: For between-trial comparisons, is the number of trials large? [ ] Not applicable: no between RCT comparison

[ ] Very small	[x] Rather small or unclear	[ ] Rather large	[ ] Large
1 or 2 or in smallest subgroup; 5 or less in continuous meta-regression	*3-4 in smallest subgroup; 6-10 in continuous meta-regression*	*5-9 in smallest subgroup; 11 to 15 in continuous meta-regression*	*10 or more in smallest subgroup; more than 15 in continuous meta-regression*

Comment:

4: Was the direction of effect modification correctly hypothesized a priori?

[ ] Definitely no	[x] Probably no or unclear	[ ] Probably yes	[ ] Definitely yes
Clearly post-hoc or results inconsistent with hypothesized direction or biologically very implausible	*Vague hypothesis or hypothesized direction unclear*	*No prior protocol available but unequivocal statement of a priori hypothesis with correct direction of effect modification*	*Prior protocol available and includes correct specification of direction of effect modification, e.g., based on a biologic rationale*

Comment:

5: Does a test for interaction suggest that chance is an unlikely explanation of the apparent effect modification? (consider irrespective of number of effect modifiers)

[ ] Chance a very likely explanation	[x] Chance a likely explanation or unclear	[ ] Chance may not explain	[ ] Chance an unlikely explanation
Interaction or meta-regression p-value >0.05	*Interaction or meta-regression p-value ≤0.05 and >0.01, or no test of interaction reported and not computable*	*Interaction or meta-regression p-value ≤0.01 and >0.005*	*Interaction or meta-regression p-value ≤0.005*

Comment: p 0.04

6: Did the authors test only a small number of effect modifiers or consider the number in their statistical analysis?

[ ] Definitely no	[x] Probably no or unclear	[ ] Probably yes	[ ] Definitely yes
Explicitly exploratory analysis or large number of effect modifiers tested (e.g., greater than 10) and multiplicity not considered in analysis	*No mention of number or 4-10 effect modifiers tested and number not considered in analysis*	*No protocol available but unequivocal statement of 3 or fewer effect modifiers tested*	*Protocol available and 3 or fewer effect modifiers tested or number considered in analysis*

Comment:

7: Did the authors use a random effects model?

[ ] Definitely no	[ ] Probably no or unclear	[ ] Probably yes	[x] Definitely yes
Fixed (or common) effect or fixed effects model explicitly stated	*Probably fixed effect(s) model*	*Probably random (or mixed) effects*	*Random (or mixed) effects explicitly stated*

Comment:

8: If the effect modifier is a continuous variable, were arbitrary cut points avoided? [x] not applicable: not continuous

[ ] Definitely no	[ ] Probably no or unclear	[ ] Probably yes	[ ] Definitely yes
Analysis based on exploratory cut point(s), e.g., picking cut point associated with highest interaction p-value	*Analysis based on cut point(s) of unclear origin*	*Analysis based on pre-specified cut point(s), e.g., suggested by prior RCT*	*Analysis based on the full continuum, e.g., assuming a linear or logarithmic relationship*

Comment:

9 Optional: Are there any additional considerations that may increase or decrease credibility? (manual section 3.9) [x] not applicable

[ ] Yes, probably decrease [] Yes, probably increase

Comment:

10: How would you rate the overall credibility of the proposed effect modification?
The overall rating should be driven by the items that decrease credibility. The following provides a sensible strategy:

- All responses definitely or probably decrease credibility or unclear → very low
- Two or more responses definitely decrease credibility → maximum usually low even if all other responses satisfy credibility criteria
- One response definitely decreases credibility → maximum usually moderate even if all other responses satisfy credibility criteria
- Two responses probably decrease credibility → maximum usually moderate even if all other responses satisfy credibility criteria
- No response options definitely or probably decrease credibility → high very likely

Place a mark on the continuous line (or type “x” in editable version)

X

Very low credibility	Low credibility	Moderate credibility	High credibility
Minimal to no support for effect modification; Use overall effect for each subgroup	Some but insufficient support for effect modification; Use overall effect for each subgroup but note remaining uncertainty	Likely effect modification; Use separate effects for each subgroup but note remaining uncertainty	Very likely effect modification; Use separate effects for each subgroup

Comment: aggregate meta-analysis, no a priori identification of the direction of the subgroup analysis, no convincing external evidence

Study	Closed-loop Total	Closed-loop Mean	Closed-loop SD	Comparator Total	Comparator Mean	Comparator SD	Mean	95% CI	Weight
**Type of Closed-Loop: Fully**									
Bally L 2018	70	0.5	0.8	66	0.6	1.4	-0.07	[-0.45; 0.31]	5.0%
Boughton CK 2021	26	0.2	0.3	26	0.4	0.9	-0.24	[-0.60; 0.12]	5.7%
Daly AB 2023	26	0.5	0.5	25	0.4	0.8	0.10	[-0.28; 0.48]	5.1%
Herzig D 2022	22	0.3	0.5	22	0.2	0.5	0.07	[-0.21; 0.35]	8.9%
Kumareswaran K 2014	12	0.0	0.0	12	0.0	0.0	0.00		0.0%
**Random effects model**	**156**			**151**			**-0.02**	**[-0.19; 0.15]**	**24.7%**
Heterogeneity: I^2 = 0%, τ^2 = 0, p = 0.5132									
**Type of Closed-Loop: Hybrid**									
Borel A 2024	17	0.2	0.3	17	0.5	0.7	-0.33	[-0.67; 0.01]	6.1%
Kudva YC 2025	214	0.4	0.4	104	0.4	0.4	0.00	[-0.09; 0.09]	68.4%
Reznik Y 2023	11	0.8	1.5	14	0.7	1.3	0.10	[-1.02; 1.22]	0.6%
Taleb N 2019	15	0.2	0.6	15	1.4	3.5	-1.20	[-3.00; 0.60]	0.2%
**Random effects model**	**257**			**150**			**-0.13**	**[-0.41; 0.15]**	**75.3%**
Heterogeneity: I^2 = 39.6%, τ^2 = 0.0320, p = 0.1745									
**Random effects model**	**413**			**301**			**-0.03**	**[-0.11; 0.06]**	**100.0%**
**Prediction interval**								**[-0.14; 0.09]**	

Heterogeneity: I^2 = 3.6%, τ^2 = 0.0005, p = 0.4023
Test for subgroup differences: χ^2_1 = 0.40, df = 1 (p = 0.5250)

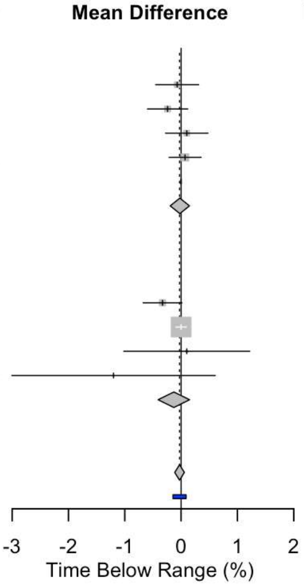

Study	Closed-loop Total	Closed-loop Mean	Closed-loop SD	Comparator Total	Comparator Mean	Comparator SD	Mean	95% CI	Weight
**Type of Closed-Loop: Fully**									
Bally L 2018	70	0.0	0.1	66	0.0	0.0	0.03		0.0%
Boughton CK 2021	26	0.0	0.0	26	0.1	0.2	-0.06	[-0.13; 0.01]	15.1%
Daly AB 2023	26	0.0	0.1	25	0.1	0.2	-0.08	[-0.18; 0.02]	8.0%
Herzig D 2022	22	0.0	0.0	22	0.0	0.0	0.00		0.0%
**Random effects model**	**144**			**139**			**-0.07**	**[-0.12; -0.01]**	**23.2%**
Heterogeneity: I^2 = 0%, τ^2 = 0, p = 0.7431									
**Type of Closed-Loop: Hybrid**									
Borel A 2024	17	0.0	0.0	17	0.0	0.0	0.01	[-0.01; 0.03]	38.5%
Kudva YC 2025	214	0.1	0.1	104	0.1	0.1	0.00	[-0.02; 0.02]	37.6%
Reznik Y 2023	11	0.2	0.5	14	0.2	0.4	0.00	[-0.36; 0.36]	0.7%
**Random effects model**	**242**			**135**			**0.01**	**[-0.01; 0.02]**	**76.8%**
Heterogeneity: I^2 = 0%, τ^2 = 0, p = 0.8195									
**Random effects model**	**386**			**274**			**-0.01**	**[-0.04; 0.02]**	**100.0%**
**Prediction interval**								**[-0.09; 0.07]**	

Heterogeneity: I^2 = 38.6%, τ^2 = 0.0005, p = 0.1642
Test for subgroup differences: χ^2_1 = 6.00, df = 1 (p = 0.0143)

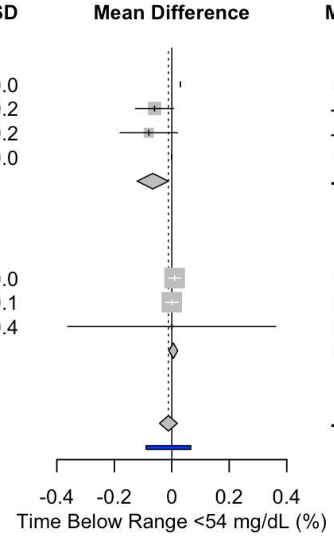

CREDIBILITY ASSESSMENT - ICEMAN

Essential preliminary considerations to define the possible effect modification of interest

State a single candidate effect modifier (e.g., age or comorbidity): fully closed-loop vs. hybrid closed-loop

Was the effect modifier measured before or at randomization? [x] yes, continue [ ] no, stop here and refer to manual for further instructions

State a single outcome and time-point (e.g., mortality at 1 year follow-up): TBR <54 mg/dl (%)

State a single effect measure (e.g., relative risk or risk difference): mean difference

1: Is the analysis of effect modification based on comparison within rather than between trials?

[x] Completely between	[ ] Mostly between or unclear	[ ] Mostly within	[ ] Completely within
Subgroup analysis or meta-regression comparing overall effects of each individual trial. This is typical for aggregate data meta-analysis.	*Subgroup analysis or meta-regression with most information coming from overall effects, but some trials providing within-trial subgroup information*	*Most trials providing within-trial subgroup information; or individual participant data analysis that combines within and between trial information*	*All trials providing within-trial subgroup information or individual participant data; and the analysis separates within from between trial information, e.g., meta-analysis of interactions*

Comment:

2: For within-trial comparisons, is the effect modification similar from trial to trial? [ ] Not applicable: no or one within-RCT comparison

[ ] Definitely not similar	[ ] Probably not similar or unclear	[ ] Mostly similar	[ ] Definitely similar
Effect modification reported for two or more trials and clearly different directions	*Effect modification not reported for individual trials or too imprecise to tell*	*Effect modification reported for two or more trials, mostly similar in direction, but considerable differences in magnitude*	*Effect modification reported for two or more trials, similar in direction, only some differences in magnitude*

Comment:

3: For between-trial comparisons, is the number of trials large? [ ] Not applicable: no between RCT comparison

[ ] Very small	[x] Rather small or unclear	[ ] Rather large	[ ] Large
1 or 2 or in smallest subgroup; 5 or less in continuous meta-regression	*3-4 in smallest subgroup; 6-10 in continuous meta-regression*	*5-9 in smallest subgroup; 11 to 15 in continuous meta-regression*	*10 or more in smallest subgroup; more than 15 in continuous meta-regression*

Comment:

4: Was the direction of effect modification correctly hypothesized a priori?

[ ] Definitely no	[x] Probably no or unclear	[ ] Probably yes	[ ] Definitely yes
Clearly post-hoc or results inconsistent with hypothesized direction or biologically very implausible	*Vague hypothesis or hypothesized direction unclear*	*No prior protocol available but unequivocal statement of a priori hypothesis with correct direction of effect modification*	*Prior protocol available and includes correct specification of direction of effect modification, e.g., based on a biologic rationale*

Comment:

5: Does a test for interaction suggest that chance is an unlikely explanation of the apparent effect modification? (consider irrespective of number of effect modifiers)

[ ] Chance a very likely explanation	[x] Chance a likely explanation or unclear	[ ] Chance may not explain	[ ] Chance an unlikely explanation
Interaction or meta-regression p-value >0.05	*Interaction or meta-regression p-value ≤0.05 and >0.01, or no test of interaction reported and not computable*	*Interaction or meta-regression p-value ≤0.01 and >0.005*	*Interaction or meta-regression p-value ≤0.005*

Comment: p 0.014

6: Did the authors test only a small number of effect modifiers or consider the number in their statistical analysis?

[ ] Definitely no	[x] Probably no or unclear	[ ] Probably yes	[ ] Definitely yes
Explicitly exploratory analysis or large number of effect modifiers tested (e.g., greater than 10) and multiplicity not considered in analysis	*No mention of number or 4-10 effect modifiers tested and number not considered in analysis*	*No protocol available but unequivocal statement of 3 or fewer effect modifiers tested*	*Protocol available and 3 or fewer effect modifiers tested or number considered in analysis*

Comment:

7: Did the authors use a random effects model?

[ ] Definitely no	[ ] Probably no or unclear	[ ] Probably yes	[x] Definitely yes
Fixed (or common) effect or fixed effects model explicitly stated	*Probably fixed effect(s) model*	*Probably random (or mixed) effects*	*Random (or mixed) effects explicitly stated*

Comment:

8: If the effect modifier is a continuous variable, were arbitrary cut points avoided? [x] not applicable: not continuous

[ ] Definitely no	[ ] Probably no or unclear	[ ] Probably yes	[ ] Definitely yes
Analysis based on exploratory cut point(s), e.g., picking cut point associated with highest interaction p-value	*Analysis based on cut point(s) of unclear origin*	*Analysis based on pre-specified cut point(s), e.g., suggested by prior RCT*	*Analysis based on the full continuum, e.g., assuming a linear or logarithmic relationship*

Comment:

9 Optional: Are there any additional considerations that may increase or decrease credibility? (manual section 3.9) [x] not applicable

[ ] Yes, probably decrease [] Yes, probably increase

Comment:

10: How would you rate the overall credibility of the proposed effect modification?
The overall rating should be driven by the items that decrease credibility. The following provides a sensible strategy:

- All responses definitely or probably decrease credibility or unclear → very low
- Two or more responses definitely decrease credibility → maximum usually low even if all other responses satisfy credibility criteria
- One response definitely decreases credibility → maximum usually moderate even if all other responses satisfy credibility criteria
- Two responses probably decrease credibility → maximum usually moderate even if all other responses satisfy credibility criteria
- No response options definitely or probably decrease credibility → high very likely

Place a mark on the continuous line (or type "x" in editable version)

X

**Very low credibility**	**Low credibility**	**Moderate credibility**	**High credibility**
Minimal to no support for effect modification; Use overall effect for each subgroup	Some but insufficient support for effect modification; Use overall effect for each subgroup but note remaining uncertainty	Likely effect modification; Use separate effects for each subgroup but note remaining uncertainty	Very likely effect modification; Use separate effects for each subgroup

Comment: aggregate meta-analysis, no a priori identification of the direction of the subgroup analysis, no convincing external evidence

Study	Closed-loop Total	Closed-loop Mean	Closed-loop SD	Comparator Total	Comparator Mean	Comparator SD	Mean	95% CI	Weight
**Type of Closed-Loop: Fully**									
Bally L 2018	70	23.6	16.6	66	49.5	22.8	-25.90	[-32.64; -19.16]	13.6%
Boughton CK 2021	26	42.6	14.3	26	56.6	25.1	-14.00	[-25.10; -2.90]	10.7%
Daly AB 2023	26	33.2	14.8	25	67.0	25.2	-33.80	[-45.20; -22.40]	10.5%
Herzig D 2022	22	15.4	8.4	22	33.9	26.4	-18.50	[-30.08; -6.92]	10.4%
Kumareswaran K 2014	12	27.7	21.8	12	36.3	34.4	-8.66	[-31.69; 14.37]	5.0%
**Random effects model**	**156**			**151**			**-22.03**	**[-29.70; -14.37]**	**50.2%**
Heterogeneity: $I^2 = 54.2\%$, $\tau^2 = 40.2371$, $p = 0.0680$									
**Type of Closed-Loop: Hybrid**									
Borel A 2024	17	23.3	11.3	17	37.7	12.1	-14.34	[-22.23; -6.45]	12.8%
Kudva YC 2025	214	35.0	16.0	104	48.0	21.0	-13.00	[-17.57; -8.43]	14.8%
Reznik Y 2023	11	36.1	8.5	14	62.7	22.9	-26.60	[-39.60; -13.60]	9.5%
Taleb N 2019	15	5.3	8.2	15	9.1	13.7	-3.80	[-11.86; 4.26]	12.7%
**Random effects model**	**257**			**150**			**-13.39**	**[-21.08; -5.71]**	**49.8%**
Heterogeneity: $I^2 = 67.6\%$, $\tau^2 = 43.6132$, $p = 0.0262$									
**Random effects model**	**413**			**301**			**-17.68**	**[-23.93; -11.44]**	**100.0%**
**Prediction interval**								**[-37.41; 2.05]**	

Heterogeneity: $I^2 = 74.6\%$, $\tau^2 = 63.0443$, $p = 0.0001$

Test for subgroup differences: $\chi^2_1 = 2.44$, df = 1 ($p = 0.1186$)

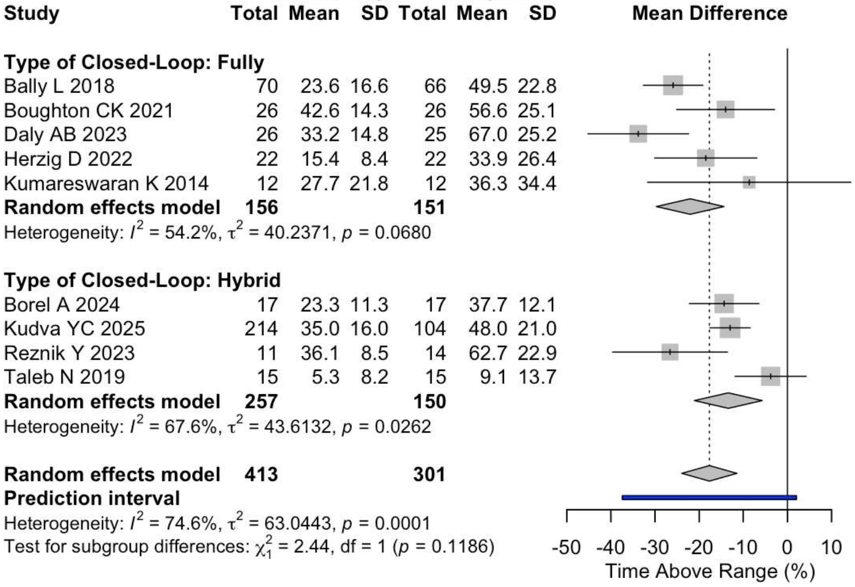

Study	Closed-loop Total	Closed-loop Mean	Closed-loop SD	Comparator Total	Comparator Mean	Comparator SD	Mean	95% CI	Weight
**Type of Closed-Loop: Hybrid**									
Borel A 2024	17	2.9	3.5	17	6.8	4.8	-3.90	[-6.71; -1.09]	41.7%
Kudva YC 2025	214	9.7	7.8	104	16.7	14.1	-7.00	[-9.90; -4.10]	41.4%
Reznik Y 2023	11	9.1	6.1	14	28.5	22.4	-19.40	[-31.65; -7.15]	16.9%
**Random effects model**	**242**			**135**			**-7.81**	**[-14.18; -1.44]**	**100.0%**
Heterogeneity: $I^2 = 72\%$, $\tau^2 = 23.3122$, $p = 0.0280$									
**Random effects model**	**242**			**135**			**-7.81**	**[-14.18; -1.44]**	**100.0%**
**Prediction interval**								**[-32.85; 17.23]**	

Heterogeneity: $I^2 = 72.0\%$, $\tau^2 = 23.3122$, $p = 0.0280$

Test for subgroup differences: $\chi^2_0 = 0.00$, df = 0 (p = NA)

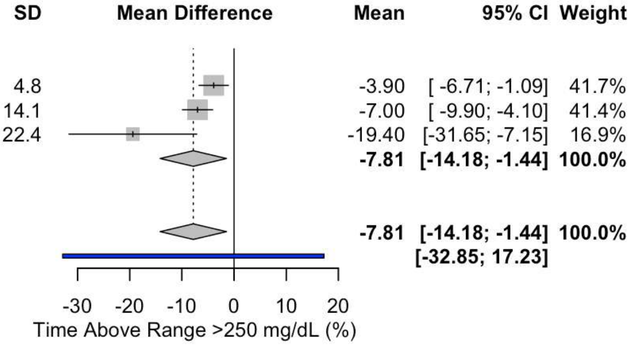

Study	Closed-loop Total	Closed-loop Mean	Closed-loop SD	Comparator Total	Comparator Mean	Comparator SD	Mean	95% CI	Weight
**Type of Closed-Loop: Fully**									
Bally L 2018	70	154.0	29.0	66	188.0	43.0	-34.00	[-46.40; -21.60]	14.3%
Boughton CK 2021	26	181.8	23.4	26	208.8	50.4	-27.00	[-48.36; -5.64]	10.6%
Daly AB 2023	26	165.6	21.6	25	226.8	54.0	-61.20	[-83.94; -38.46]	10.0%
Herzig D 2022	22	144.0	12.6	22	169.2	45.0	-25.20	[-44.73; -5.67]	11.3%
Kumareswaran K 2014	12	169.2	34.2	12	174.6	25.2	-5.40	[-29.44; 18.64]	9.6%
**Random effects model**	**156**			**151**			**-30.84**	**[-46.87; -14.80]**	**55.8%**
Heterogeneity: $I^2 = 66.1\%$, $\tau^2 = 230.8005$, $p = 0.0188$									
**Type of Closed-Loop: Hybrid**									
Borel A 2024	17	158.8	17.3	17	172.2	20.8	-13.40	[-26.26; -0.54]	14.1%
Kudva YC 2025	214	170.0	23.0	104	188.0	34.0	-18.00	[-25.22; -10.78]	16.3%
Taleb N 2019	15	126.0	16.2	15	133.2	21.6	-7.20	[-20.86; 6.46]	13.8%
**Random effects model**	**246**			**136**			**-14.94**	**[-21.12; -8.76]**	**44.2%**
Heterogeneity: $I^2 = 0\%$, $\tau^2 = 3.1524$, $p = 0.3736$									
**Random effects model**	**402**			**287**			**-23.05**	**[-34.06; -12.03]**	**100.0%**
**Prediction interval**								**[-57.46; 11.37]**	

Heterogeneity: $I^2 = 71.1\%$, $\tau^2 = 180.2801$, $p = 0.0010$
Test for subgroup differences: $\chi^2_1 = 3.29$, df = 1 ($p = 0.0698$)

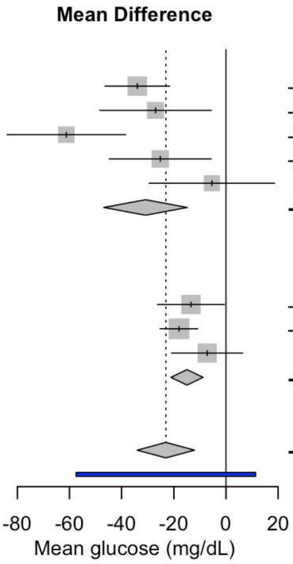

CREDIBILITY ASSESSMENT - ICEMAN

Essential preliminary considerations to define the possible effect modification of interest

State a single candidate effect modifier (e.g., age or comorbidity): fully closed-loop vs. hybrid closed-loop

Was the effect modifier measured before or at randomization? [x] yes, continue [ ] no, stop here and refer to manual for further instructions

State a single outcome and time-point (e.g., mortality at 1 year follow-up): mean glucose (mg/dl)

State a single effect measure (e.g., relative risk or risk difference): mean difference

1: Is the analysis of effect modification based on comparison within rather than between trials?

[x] Completely between	[ ] Mostly between or unclear	[ ] Mostly within	[ ] Completely within
Subgroup analysis or meta-regression comparing overall effects of each individual trial. This is typical for aggregate data meta-analysis.	*Subgroup analysis or meta-regression with most information coming from overall effects, but some trials providing within-trial subgroup information*	*Most trials providing within-trial subgroup information; or individual participant data analysis that combines within and between trial information*	*All trials providing within-trial subgroup information or individual participant data; and the analysis separates within from between trial information, e.g., meta-analysis of interactions*

Comment:

2: For within-trial comparisons, is the effect modification similar from trial to trial? [ ] Not applicable: no or one within-RCT comparison

[ ] Definitely not similar	[ ] Probably not similar or unclear	[ ] Mostly similar	[ ] Definitely similar
Effect modification reported for two or more trials and clearly different directions	*Effect modification not reported for individual trials or too imprecise to tell*	*Effect modification reported for two or more trials, mostly similar in direction, but considerable differences in magnitude*	*Effect modification reported for two or more trials, similar in direction, only some differences in magnitude*

Comment:

3: For between-trial comparisons, is the number of trials large? [ ] Not applicable: no between RCT comparison

[ ] Very small	[x] Rather small or unclear	[ ] Rather large	[ ] Large
1 or 2 or in smallest subgroup; 5 or less in continuous meta-regression	*3-4 in smallest subgroup; 6-10 in continuous meta-regression*	*5-9 in smallest subgroup; 11 to 15 in continuous meta-regression*	*10 or more in smallest subgroup; more than 15 in continuous meta-regression*

Comment:

4: Was the direction of effect modification correctly hypothesized a priori?

[ ] Definitely no	[x] Probably no or unclear	[ ] Probably yes	[ ] Definitely yes
Clearly post-hoc or results inconsistent with hypothesized direction or biologically very implausible	*Vague hypothesis or hypothesized direction unclear*	*No prior protocol available but unequivocal statement of a priori hypothesis with correct direction of effect modification*	*Prior protocol available and includes correct specification of direction of effect modification, e.g., based on a biologic rationale*

Comment:

5: Does a test for interaction suggest that chance is an unlikely explanation of the apparent effect modification? (consider irrespective of number of effect modifiers)

[x] Chance a very likely explanation	[ ] Chance a likely explanation or unclear	[ ] Chance may not explain	[ ] Chance an unlikely explanation
Interaction or meta-regression p-value >0.05	*Interaction or meta-regression p-value ≤0.05 and >0.01, or no test of interaction reported and not computable*	*Interaction or meta-regression p-value ≤0.01 and >0.005*	*Interaction or meta-regression p-value ≤0.005*

Comment: p 0.069

6: Did the authors test only a small number of effect modifiers or consider the number in their statistical analysis?

[ ] Definitely no	[x] Probably no or unclear	[ ] Probably yes	[ ] Definitely yes
Explicitly exploratory analysis or large number of effect modifiers tested (e.g., greater than 10) and multiplicity not considered in analysis	*No mention of number or 4-10 effect modifiers tested and number not considered in analysis*	*No protocol available but unequivocal statement of 3 or fewer effect modifiers tested*	*Protocol available and 3 or fewer effect modifiers tested or number considered in analysis*

Comment:

7: Did the authors use a random effects model?

[ ] Definitely no	[ ] Probably no or unclear	[ ] Probably yes	[x] Definitely yes
Fixed (or common) effect or fixed effects model explicitly stated	*Probably fixed effect(s) model*	*Probably random (or mixed) effects*	*Random (or mixed) effects explicitly stated*

Comment:

8: If the effect modifier is a continuous variable, were arbitrary cut points avoided? [x] not applicable: not continuous

[ ] Definitely no	[ ] Probably no or unclear	[ ] Probably yes	[ ] Definitely yes
Analysis based on exploratory cut point(s), e.g., picking cut point associated with highest interaction p-value	*Analysis based on cut point(s) of unclear origin*	*Analysis based on pre-specified cut point(s), e.g., suggested by prior RCT*	*Analysis based on the full continuum, e.g., assuming a linear or logarithmic relationship*

Comment:

9 Optional: Are there any additional considerations that may increase or decrease credibility? (manual section 3.9) [x] not applicable

[ ] Yes, probably decrease [] Yes, probably increase

Comment:

10: How would you rate the overall credibility of the proposed effect modification?

The overall rating should be driven by the items that decrease credibility. The following provides a sensible strategy:

- All responses definitely or probably decrease credibility or unclear → very low
- Two or more responses definitely decrease credibility → maximum usually low even if all other responses satisfy credibility criteria
- One response definitely decreases credibility → maximum usually moderate even if all other responses satisfy credibility criteria
- Two responses probably decrease credibility → maximum usually moderate even if all other responses satisfy credibility criteria
- No response options definitely or probably decrease credibility → high very likely

Place a mark on the continuous line (or type "x" in editable version)

x

Very low credibility	Low credibility	Moderate credibility	High credibility
Minimal to no support for effect modification; Use overall effect for each subgroup	Some but insufficient support for effect modification; Use overall effect for each subgroup but note remaining uncertainty	Likely effect modification; Use separate effects for each subgroup but note remaining uncertainty	Very likely effect modification; Use separate effects for each subgroup

Comment: aggregate meta-analysis, no a priori identification of the direction of the subgroup analysis, no convincing external evidence

Study	Closed-loop Total	Closed-loop Mean	Closed-loop SD	Comparator Total	Comparator Mean	Comparator SD	Mean	95% CI	Weight
**Type of Closed-Loop: Fully**									
Bally L 2018	70	29.4	6.4	66	31.5	9.3	-2.10	[-4.80; 0.60]	14.7%
Boughton CK 2021	26	31.7	4.8	26	31.5	5.4	0.20	[-2.58; 2.98]	14.5%
Daly AB 2023	26	32.2	5.7	25	27.7	8.5	4.50	[0.51; 8.49]	10.8%
Herzig D 2022	22	25.4	3.9	22	28.5	7.1	-3.10	[-6.48; 0.28]	12.6%
**Random effects model**	**144**			**139**			**-0.29**	**[-3.34; 2.75]**	**52.6%**
Heterogeneity: I^2 = 69.6%, τ^2 = 6.9723, p = 0.0196									
**Type of Closed-Loop: Hybrid**									
Borel A 2024	17	23.0	3.3	17	25.1	2.9	-2.10	[-4.19; -0.01]	16.8%
Kudva YC 2025	214	30.0	5.0	104	29.0	5.0	1.00	[-0.17; 2.17]	19.6%
Reznik Y 2023	11	31.5	5.7	14	28.2	3.9	3.30	[-0.64; 7.24]	11.0%
**Random effects model**	**242**			**135**			**0.44**	**[-2.35; 3.23]**	**47.4%**
Heterogeneity: I^2 = 76.7%, τ^2 = 4.5672, p = 0.0137									
**Random effects model**	**386**			**274**			**0.02**	**[-1.85; 1.90]**	**100.0%**
**Prediction interval**								**[-5.58; 5.63]**	
Heterogeneity: I^2 = 69.6%, τ^2 = 4.3268, p = 0.0031									
Test for subgroup differences: χ^2_1 = 0.12, df = 1 (p = 0.7278)									

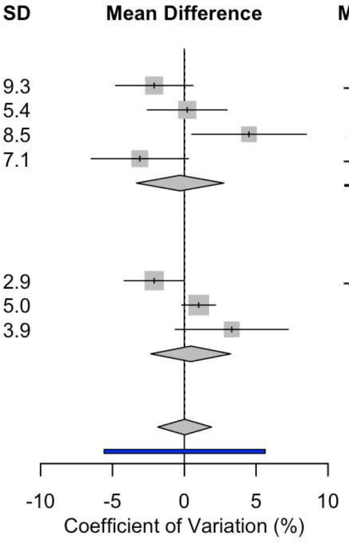

Study	Closed-loop Total	Closed-loop Mean	Closed-loop SD	Comparator Total	Comparator Mean	Comparator SD	Mean	95% CI	Weight
**Type of Closed-Loop: Fully**									
Bally L 2018	70	46.0	19.0	66	59.0	19.0	-13.00	[-19.39; -6.61]	18.2%
Boughton CK 2021	26	57.6	12.6	26	64.8	16.2	-7.20	[-15.09; 0.69]	16.0%
Daly AB 2023	26	54.0	14.4	25	61.2	18.0	-7.20	[-16.17; 1.77]	14.5%
Herzig D 2022	22	37.8	7.2	22	46.8	14.4	-9.00	[-15.73; -2.27]	17.7%
Kumareswaran K 2014	12	39.6	12.6	12	32.4	7.2	7.20	[-1.01; 15.41]	15.5%
**Random effects model**	**156**			**151**			**-6.08**	**[-12.74; 0.59]**	**81.9%**
Heterogeneity: I^2 = 73.7%, τ^2 = 42.6363, p = 0.0043									
**Type of Closed-Loop: Hybrid**									
Borel A 2024	17	43.0	11.0	17	50.0	8.0	-7.00	[-13.47; -0.53]	18.1%
**Random effects model**	**173**			**168**			**-6.30**	**[-11.64; -0.96]**	**100.0%**
**Prediction interval**								**[-22.05; 9.46]**	
Heterogeneity: I^2 = 67.1%, τ^2 = 30.1501, p = 0.0095									
Test for subgroup differences: χ^2_1 = 0.04, df = 1 (p = 0.8452)									

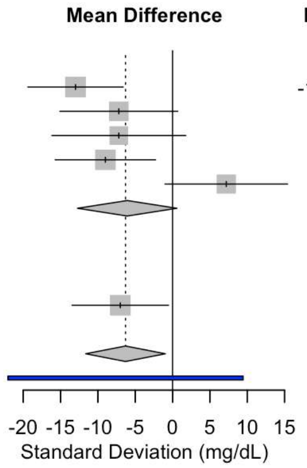

Study	Closed-loop Total	Closed-loop Mean	Closed-loop SD	Comparator Total	Comparator Mean	Comparator SD	Mean	95% CI	Weight
**Type of Closed-Loop: Fully**									
Daly AB 2023	26	7.3	0.8	25	8.7	1.2	-1.40	[-1.96; -0.84]	31.8%
**Type of Closed-Loop: Hybrid**									
Kudva YC 2025	209	7.3	0.9	102	7.7	1.1	-0.40	[-0.65; -0.15]	38.1%
Reznik Y 2023	11	7.4	0.6	14	8.8	1.0	-1.40	[-2.03; -0.77]	30.1%
**Random effects model**	**220**			**116**			**-0.86**	**[-1.83; 0.12]**	**68.2%**
Heterogeneity: I^2 = 88%, τ^2 = 0.4401, p = 0.0039									
**Random effects model**	**246**			**141**			**-1.02**	**[-1.71; -0.32]**	**100.0%**
**Prediction interval**								**[-3.87; 1.84]**	
Heterogeneity: I^2 = 87.7%, τ^2 = 0.3145, p = 0.0003									
Test for subgroup differences: χ^2_1 = 0.90, df = 1 (p = 0.3437)									

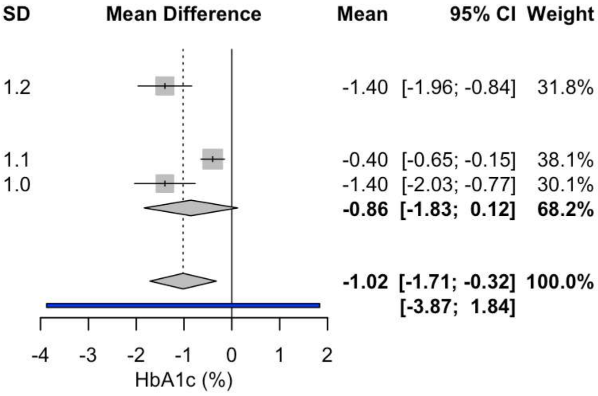

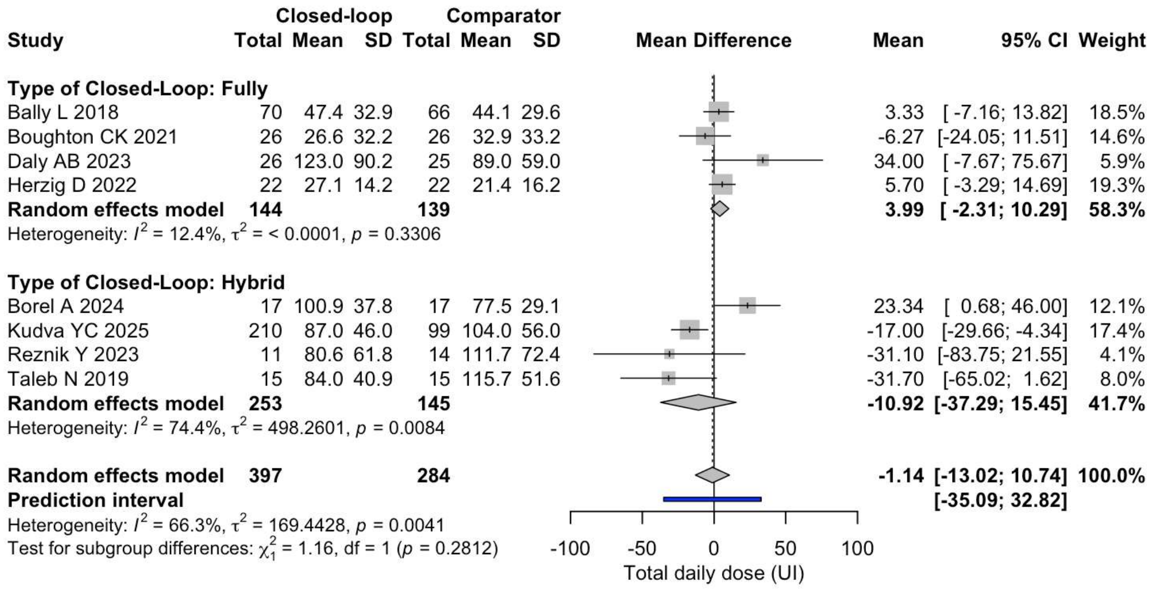

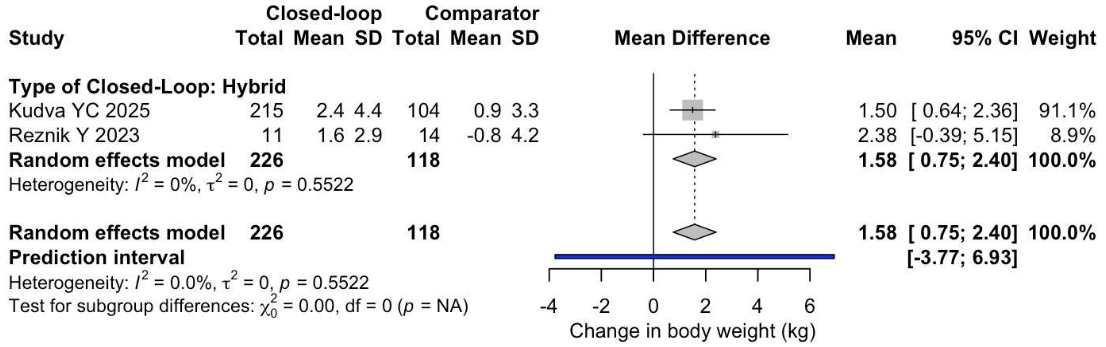

Each forest plot displays the pooled mean difference with corresponding 95% confidence intervals for each study, based on random-effects meta-analyses. The overall pooled estimate and its prediction interval are reported at the bottom of each plot. Measures of heterogeneity and subgroup differences are also provided when applicable. SD, standard deviation, CI, confidence interval.

Supplement 8. Subgroup analysis, inpatient vs. outpatient care setting

Study	Closed-loop Total	Closed-loop Mean	Closed-loop SD	Comparator Total	Comparator Mean	Comparator SD	Mean	95% CI	Weight
**Care setting: Inpatient**									
Bally L 2018	70	75.9	28.5	66	49.9	34.4	26.00	[15.35; 36.65]	11.2%
Herzig D 2022	22	84.2	8.6	22	64.3	25.4	19.90	[8.69; 31.11]	10.8%
**Random effects model**	**92**			**88**			**23.11**	**[15.39; 30.82]**	**22.0%**
Heterogeneity: $I^2 = 0\%$, $\tau^2 = 0$, $p = 0.4393$									
**Care setting: Outpatient**									
Borel A 2024	17	76.3	12.1	17	62.0	12.1	14.33	[6.18; 22.48]	13.1%
Boughton CK 2021	26	57.1	14.3	26	42.5	24.7	14.60	[3.63; 25.57]	11.0%
Daly AB 2023	26	66.3	14.9	25	32.3	24.7	34.00	[22.75; 45.25]	10.8%
Kudva YC 2025	214	64.0	16.0	104	52.0	21.0	12.00	[7.43; 16.57]	15.7%
Kumareswaran K 2014	12	44.7	28.5	12	23.0	34.4	21.67	[-3.60; 46.94]	4.3%
Reznik Y 2023	11	63.0	9.4	14	36.6	22.0	26.40	[13.61; 39.19]	9.7%
Taleb N 2019	15	90.4	8.1	15	84.2	13.2	6.20	[-1.64; 14.04]	13.4%
**Random effects model**	**321**			**213**			**17.29**	**[9.95; 24.63]**	**78.0%**
Heterogeneity: $I^2 = 70.9\%$, $\tau^2 = 67.0716$, $p = 0.0021$									
**Random effects model**	**413**			**301**			**18.43**	**[12.40; 24.46]**	**100.0%**
**Prediction interval**								**[-0.07; 36.92]**	
Heterogeneity: $I^2 = 68.6\%$, $\tau^2 = 54.8663$, $p = 0.0013$									
Test for subgroup differences: $\chi^2_1 = 1.14$, df = 1 ($p = 0.2847$)									

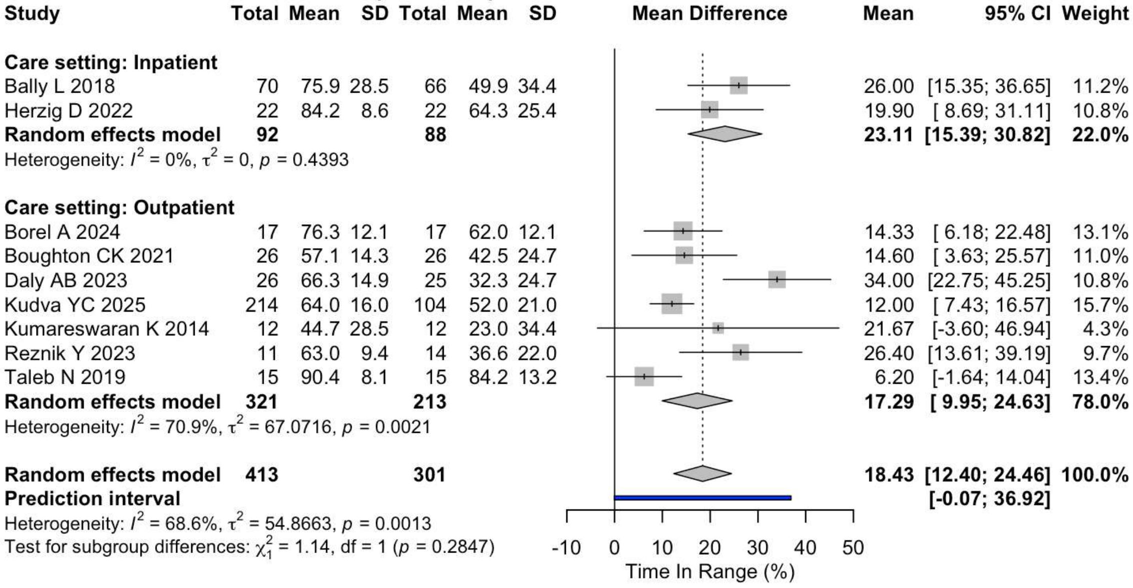

Study	Closed-loop Total	Closed-loop Mean	Closed-loop SD	Comparator Total	Comparator Mean	Comparator SD	Mean	95% CI	Weight
**Care setting: Inpatient**									
Bally L 2018	70	0.5	0.8	66	0.6	1.4	-0.07	[-0.45; 0.31]	5.0%
Herzig D 2022	22	0.3	0.5	22	0.2	0.5	0.07	[-0.21; 0.35]	8.9%
**Random effects model**	**92**			**88**			**0.02**	**[-0.21; 0.25]**	**13.9%**
Heterogeneity: $I^2 = 0\%$, $\tau^2 = 0$, $p = 0.5637$									
**Care setting: Outpatient**									
Borel A 2024	17	0.2	0.3	17	0.5	0.7	-0.33	[-0.67; 0.01]	6.1%
Boughton CK 2021	26	0.2	0.3	26	0.4	0.9	-0.24	[-0.60; 0.12]	5.7%
Daly AB 2023	26	0.5	0.5	25	0.4	0.8	0.10	[-0.28; 0.48]	5.1%
Kudva YC 2025	214	0.4	0.4	104	0.4	0.4	0.00	[-0.09; 0.09]	68.4%
Kumareswaran K 2014	12	0.0	0.0	12	0.0	0.0	0.00		0.0%
Reznik Y 2023	11	0.8	1.5	14	0.7	1.3	0.10	[-1.02; 1.22]	0.6%
Taleb N 2019	15	0.2	0.6	15	1.4	3.5	-1.20	[-3.00; 0.60]	0.2%
**Random effects model**	**321**			**213**			**-0.09**	**[-0.26; 0.08]**	**86.1%**
Heterogeneity: $I^2 = 26\%$, $\tau^2 = 0.0127$, $p = 0.2392$									
**Random effects model**	**413**			**301**			**-0.03**	**[-0.11; 0.06]**	**100.0%**
**Prediction interval**								**[-0.14; 0.09]**	
Heterogeneity: $I^2 = 3.6\%$, $\tau^2 = 0.0005$, $p = 0.4023$									
Test for subgroup differences: $\chi^2_1 = 0.55$, df = 1 ($p = 0.4565$)									

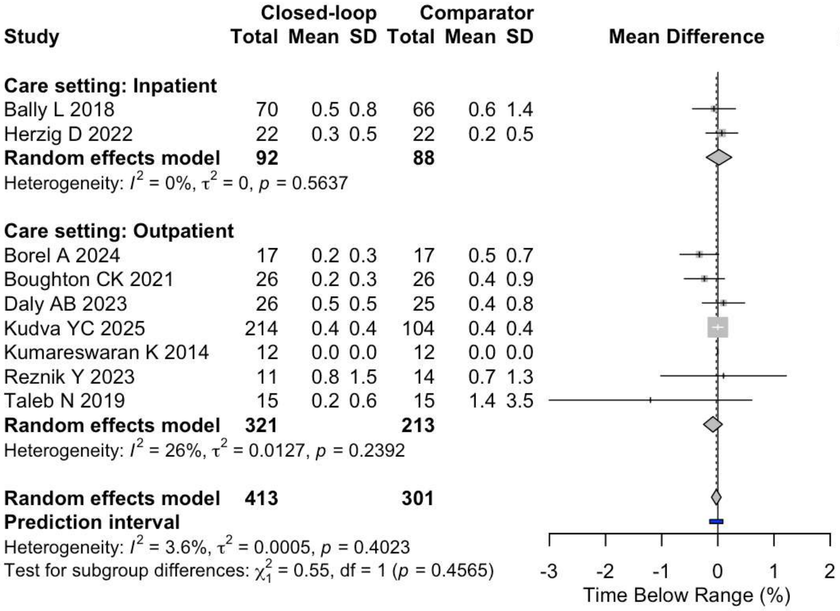

Study	Closed-loop Total	Closed-loop Mean	Closed-loop SD	Comparator Total	Comparator Mean	Comparator SD	Mean	95% CI	Weight
**Care setting: Inpatient**									
Bally L 2018	70	0.0	0.1	66	0.0	0.0	0.03		0.0%
Herzig D 2022	22	0.0	0.0	22	0.0	0.0	0.00		0.0%
**Care setting: Outpatient**									
Borel A 2024	17	0.0	0.0	17	0.0	0.0	0.01	[-0.01; 0.03]	38.5%
Boughton CK 2021	26	0.0	0.0	26	0.1	0.2	-0.06	[-0.13; 0.01]	15.1%
Daly AB 2023	26	0.0	0.1	25	0.1	0.2	-0.08	[-0.18; 0.02]	8.0%
Kudva YC 2025	214	0.1	0.1	104	0.1	0.1	0.00	[-0.02; 0.02]	37.6%
Reznik Y 2023	11	0.2	0.5	14	0.2	0.4	0.00	[-0.36; 0.36]	0.7%
**Random effects model**	**294**			**186**			**-0.01**	**[-0.04; 0.02]**	**100.0%**
Heterogeneity: $I^2 = 38.6\%$, $\tau^2 = 0.0005$, $p = 0.1642$									
**Random effects model**	**386**			**274**			**-0.01**	**[-0.04; 0.02]**	**100.0%**
**Prediction interval**								**[-0.09; 0.07]**	

Heterogeneity: $I^2 = 38.6\%$, $\tau^2 = 0.0005$, $p = 0.1642$
Test for subgroup differences: $\chi^2_0 = 0.00$, df = 0 (p = NA)

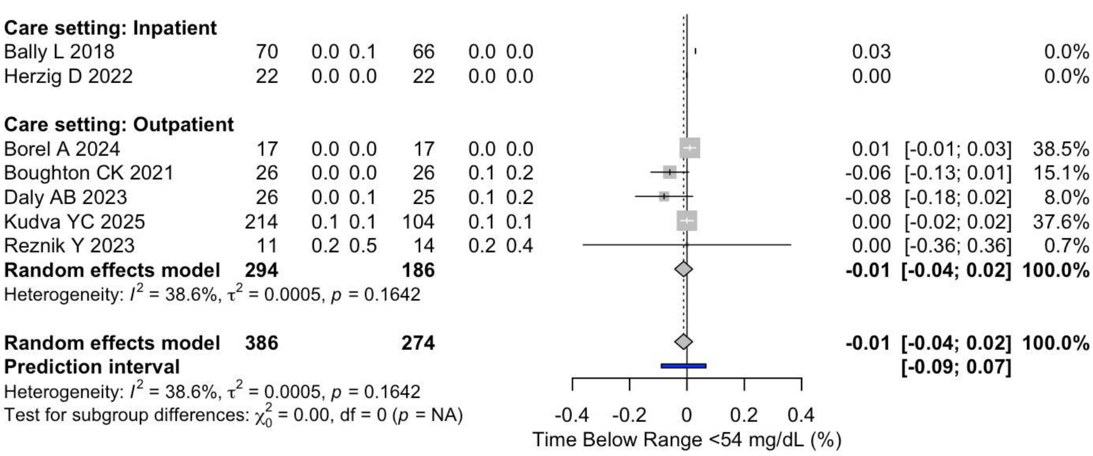

Study	Closed-loop Total	Closed-loop Mean	Closed-loop SD	Comparator Total	Comparator Mean	Comparator SD	Mean	95% CI	Weight
**Care setting: Inpatient**									
Bally L 2018	70	23.6	16.6	66	49.5	22.8	-25.90	[-32.64; -19.16]	13.6%
Herzig D 2022	22	15.4	8.4	22	33.9	26.4	-18.50	[-30.08; -6.92]	10.4%
**Random effects model**	**92**			**88**			**-23.76**	**[-30.34; -17.18]**	**23.9%**
Heterogeneity: $I^2 = 14.7\%$, $\tau^2 = 4.0299$, $p = 0.2789$									
**Care setting: Outpatient**									
Borel A 2024	17	23.3	11.3	17	37.7	12.1	-14.34	[-22.23; -6.45]	12.8%
Boughton CK 2021	26	42.6	14.3	26	56.6	25.1	-14.00	[-25.10; -2.90]	10.7%
Daly AB 2023	26	33.2	14.8	25	67.0	25.2	-33.80	[-45.20; -22.40]	10.5%
Kudva YC 2025	214	35.0	16.0	104	48.0	21.0	-13.00	[-17.57; -8.43]	14.8%
Kumareswaran K 2014	12	27.7	21.8	12	36.3	34.4	-8.66	[-31.69; 14.37]	5.0%
Reznik Y 2023	11	36.1	8.5	14	62.7	22.9	-26.60	[-39.60; -13.60]	9.5%
Taleb N 2019	15	5.3	8.2	15	9.1	13.7	-3.80	[-11.86; 4.26]	12.7%
**Random effects model**	**321**			**213**			**-16.17**	**[-23.78; -8.55]**	**76.1%**
Heterogeneity: $I^2 = 72.4\%$, $\tau^2 = 74.4783$, $p = 0.0014$									
**Random effects model**	**413**			**301**			**-17.68**	**[-23.93; -11.44]**	**100.0%**
**Prediction interval**								**[-37.41; 2.05]**	

Heterogeneity: $I^2 = 74.6\%$, $\tau^2 = 63.0443$, $p = 0.0001$
Test for subgroup differences: $\chi^2_1 = 2.19$, df = 1 ($p = 0.1392$)

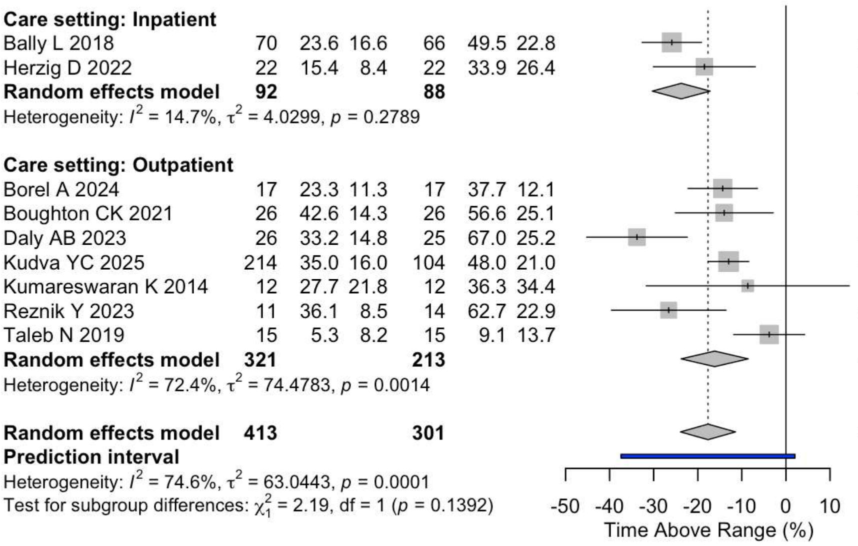

Study	Closed-loop Total	Closed-loop Mean	Closed-loop SD	Comparator Total	Comparator Mean	Comparator SD	Mean	95% CI	Weight
**Care setting: Outpatient**									
Borel A 2024	17	2.9	3.5	17	6.8	4.8	-3.90	[-6.71; -1.09]	41.7%
Kudva YC 2025	214	9.7	7.8	104	16.7	14.1	-7.00	[-9.90; -4.10]	41.4%
Reznik Y 2023	11	9.1	6.1	14	28.5	22.4	-19.40	[-31.65; -7.15]	16.9%
**Random effects model**	**242**			**135**			**-7.81**	**[-14.18; -1.44]**	**100.0%**
Heterogeneity: $I^2 = 72\%$, $\tau^2 = 23.3122$, $p = 0.0280$									
**Random effects model**	**242**			**135**			**-7.81**	**[-14.18; -1.44]**	**100.0%**
**Prediction interval**								**[-32.85; 17.23]**	

Heterogeneity: $I^2 = 72.0\%$, $\tau^2 = 23.3122$, $p = 0.0280$
Test for subgroup differences: $\chi^2_0 = 0.00$, df = 0 (p = NA)

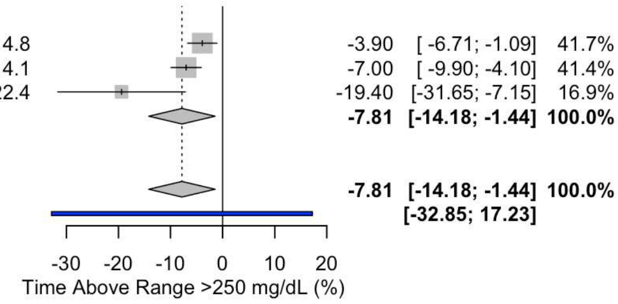

Study	Closed-loop Total	Closed-loop Mean	Closed-loop SD	Comparator Total	Comparator Mean	Comparator SD	Mean	95% CI	Weight
**Care setting: Inpatient**									
Bally L 2018	70	154.0	29.0	66	188.0	43.0	-34.00	[-46.40; -21.60]	14.3%
Herzig D 2022	22	144.0	12.6	22	169.2	45.0	-25.20	[-44.73; -5.67]	11.3%
**Random effects model**	**92**			**88**			**-31.47**	**[-41.94; -21.00]**	**25.6%**
Heterogeneity: I^2 = 0%, τ^2 = 0, p = 0.4559									
**Care setting: Outpatient**									
Borel A 2024	17	158.8	17.3	17	172.2	20.8	-13.40	[-26.26; -0.54]	14.1%
Boughton CK 2021	26	181.8	23.4	26	208.8	50.4	-27.00	[-48.36; -5.64]	10.6%
Daly AB 2023	26	165.6	21.6	25	226.8	54.0	-61.20	[-83.94; -38.46]	10.0%
Kudva YC 2025	214	170.0	23.0	104	188.0	34.0	-18.00	[-25.22; -10.78]	16.3%
Kumareswaran K 2014	12	169.2	34.2	12	174.6	25.2	-5.40	[-29.44; 18.64]	9.6%
Taleb N 2019	15	126.0	16.2	15	133.2	21.6	-7.20	[-20.86; 6.46]	13.8%
**Random effects model**	**310**			**199**			**-20.95**	**[-35.66; -6.23]**	**74.4%**
Heterogeneity: I^2 = 73%, τ^2 = 261.4109, p = 0.0024									
**Random effects model**	**402**			**287**			**-23.05**	**[-34.06; -12.03]**	**100.0%**
**Prediction interval**								**[-57.46; 11.37]**	
Heterogeneity: I^2 = 71.1%, τ^2 = 180.2801, p = 0.0010									
Test for subgroup differences: χ^2_1 = 1.30, df = 1 (p = 0.2534)									

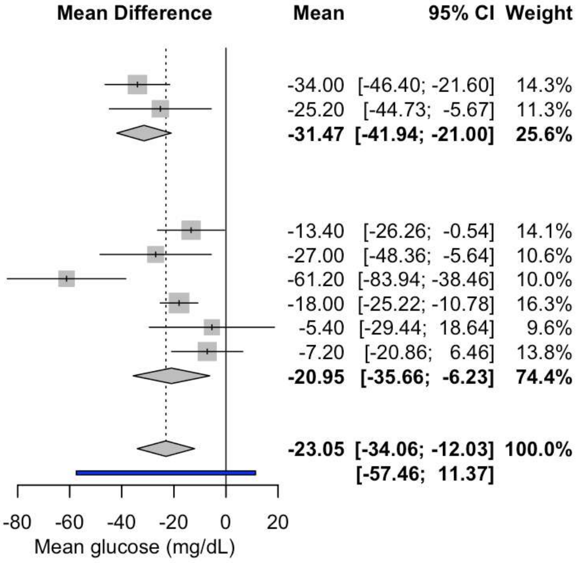

Study	Closed-loop Total	Closed-loop Mean	Closed-loop SD	Comparator Total	Comparator Mean	Comparator SD	Mean	95% CI	Weight
**Care setting: Inpatient**									
Bally L 2018	70	29.4	6.4	66	31.5	9.3	-2.10	[-4.80; 0.60]	14.7%
Herzig D 2022	22	25.4	3.9	22	28.5	7.1	-3.10	[-6.48; 0.28]	12.6%
**Random effects model**	**92**			**88**			**-2.49**	**[-4.60; -0.38]**	**27.3%**
Heterogeneity: I^2 = 0%, τ^2 = 0, p = 0.6507									
**Care setting: Outpatient**									
Borel A 2024	17	23.0	3.3	17	25.1	2.9	-2.10	[-4.19; -0.01]	16.8%
Boughton CK 2021	26	31.7	4.8	26	31.5	5.4	0.20	[-2.58; 2.98]	14.5%
Daly AB 2023	26	32.2	5.7	25	27.7	8.5	4.50	[0.51; 8.49]	10.8%
Kudva YC 2025	214	30.0	5.0	104	29.0	5.0	1.00	[-0.17; 2.17]	19.6%
Reznik Y 2023	11	31.5	5.7	14	28.2	3.9	3.30	[-0.64; 7.24]	11.0%
**Random effects model**	**294**			**186**			**0.97**	**[-1.15; 3.08]**	**72.7%**
Heterogeneity: I^2 = 67.8%, τ^2 = 3.8577, p = 0.0145									
**Random effects model**	**386**			**274**			**0.02**	**[-1.85; 1.90]**	**100.0%**
**Prediction interval**								**[-5.58; 5.63]**	
Heterogeneity: I^2 = 69.6%, τ^2 = 4.3268, p = 0.0031									
Test for subgroup differences: χ^2_1 = 5.14, df = 1 (p = 0.0234)									

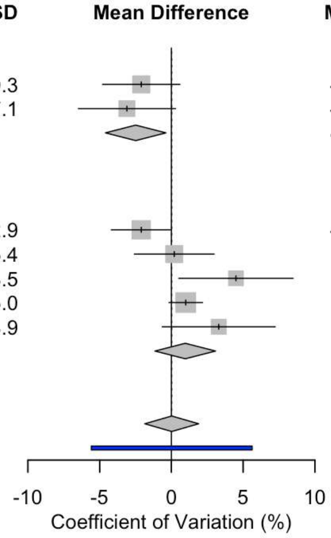

CREDIBILITY ASSESSMENT - ICEMAN

Essential preliminary considerations to define the possible effect modification of interest

State a single candidate effect modifier (e.g., age or comorbidity): inpatient vs. outpatient care setting

Was the effect modifier measured before or at randomization? [x] yes, continue [ ] no, stop here and refer to manual for further instructions

State a single outcome and time-point (e.g., mortality at 1 year follow-up): CV (%)

State a single effect measure (e.g., relative risk or risk difference): mean difference

1: Is the analysis of effect modification based on comparison within rather than between trials?

[x] Completely between	[ ] Mostly between or unclear	[ ] Mostly within	[ ] Completely within
Subgroup analysis or meta-regression comparing overall effects of each individual trial. This is typical for aggregate data meta-analysis.	*Subgroup analysis or meta-regression with most information coming from overall effects, but some trials providing within-trial subgroup information*	*Most trials providing within-trial subgroup information; or individual participant data analysis that combines within and between trial information*	*All trials providing within-trial subgroup information or individual participant data; and the analysis separates within from between trial information, e.g., meta-analysis of interactions*

Comment:

2: For within-trial comparisons, is the effect modification similar from trial to trial? [ ] Not applicable: no or one within-RCT comparison

[ ] Definitely not similar	[ ] Probably not similar or unclear	[ ] Mostly similar	[ ] Definitely similar
Effect modification reported for two or more trials and clearly different directions	*Effect modification not reported for individual trials or too imprecise to tell*	*Effect modification reported for two or more trials, mostly similar in direction, but considerable differences in magnitude*	*Effect modification reported for two or more trials, similar in direction, only some differences in magnitude*

Comment:

3: For between-trial comparisons, is the number of trials large? [ ] Not applicable: no between RCT comparison

[x] Very small	[ ] Rather small or unclear	[ ] Rather large	[ ] Large
1 or 2 or in smallest subgroup; 5 or less in continuous meta-regression	*3-4 in smallest subgroup; 6-10 in continuous meta-regression*	*5-9 in smallest subgroup; 11 to 15 in continuous meta-regression*	*10 or more in smallest subgroup; more than 15 in continuous meta-regression*

Comment:

4: Was the direction of effect modification correctly hypothesized a priori?

[ ] Definitely no	[x] Probably no or unclear	[ ] Probably yes	[ ] Definitely yes
Clearly post-hoc or results inconsistent with hypothesized direction or biologically very implausible	*Vague hypothesis or hypothesized direction unclear*	*No prior protocol available but unequivocal statement of a priori hypothesis with correct direction of effect modification*	*Prior protocol available and includes correct specification of direction of effect modification, e.g., based on a biologic rationale*

Comment:

5: Does a test for interaction suggest that chance is an unlikely explanation of the apparent effect modification? (consider irrespective of number of effect modifiers)

[ ] Chance a very likely explanation	[x] Chance a likely explanation or unclear	[ ] Chance may not explain	[ ] Chance an unlikely explanation
Interaction or meta-regression p-value >0.05	*Interaction or meta-regression p-value ≤0.05 and >0.01, or no test of interaction reported and not computable*	*Interaction or meta-regression p-value ≤0.01 and >0.005*	*Interaction or meta-regression p-value ≤0.005*

Comment: p 0.02

6: Did the authors test only a small number of effect modifiers or consider the number in their statistical analysis?

[ ] Definitely no	[x] Probably no or unclear	[ ] Probably yes	[ ] Definitely yes
Explicitly exploratory analysis or large number of effect modifiers tested (e.g., greater than 10) and multiplicity not considered in analysis	*No mention of number or 4-10 effect modifiers tested and number not considered in analysis*	*No protocol available but unequivocal statement of 3 or fewer effect modifiers tested*	*Protocol available and 3 or fewer effect modifiers tested or number considered in analysis*

Comment:

7: Did the authors use a random effects model?

[ ] Definitely no	[ ] Probably no or unclear	[ ] Probably yes	[x] Definitely yes
Fixed (or common) effect or fixed effects model explicitly stated	*Probably fixed effect(s) model*	*Probably random (or mixed) effects*	*Random (or mixed) effects explicitly stated*

Comment:

8: If the effect modifier is a continuous variable, were arbitrary cut points avoided? [x] not applicable: not continuous

[ ] Definitely no	[ ] Probably no or unclear	[ ] Probably yes	[ ] Definitely yes
Analysis based on exploratory cut point(s), e.g., picking cut point associated with highest interaction p-value	*Analysis based on cut point(s) of unclear origin*	*Analysis based on pre-specified cut point(s), e.g., suggested by prior RCT*	*Analysis based on the full continuum, e.g., assuming a linear or logarithmic relationship*

Comment:

9 Optional: Are there any additional considerations that may increase or decrease credibility? (manual section 3.9) [x] not applicable

[ ] Yes, probably decrease [] Yes, probably increase

Comment:

10: How would you rate the overall credibility of the proposed effect modification?
The overall rating should be driven by the items that decrease credibility. The following provides a sensible strategy:

- All responses definitely or probably decrease credibility or unclear → very low
- Two or more responses definitely decrease credibility → maximum usually low even if all other responses satisfy credibility criteria
- One response definitely decreases credibility → maximum usually moderate even if all other responses satisfy credibility criteria
- Two responses probably decrease credibility → maximum usually moderate even if all other responses satisfy credibility criteria
- No response options definitely or probably decrease credibility → high very likely

Place a mark on the continuous line (or type "x" in editable version)

Very low credibility	Low credibility **X**	Moderate credibility	High credibility
Minimal to no support for effect modification; Use overall effect for each subgroup	Some but insufficient support for effect modification; Use overall effect for each subgroup but note remaining uncertainty	Likely effect modification; Use separate effects for each subgroup but note remaining uncertainty	Very likely effect modification; Use separate effects for each subgroup

Comment: aggregate meta-analysis, very small number of studies in the smaller subgroup, no a priori identification of the direction of the subgroup analysis, no convincing external evidence

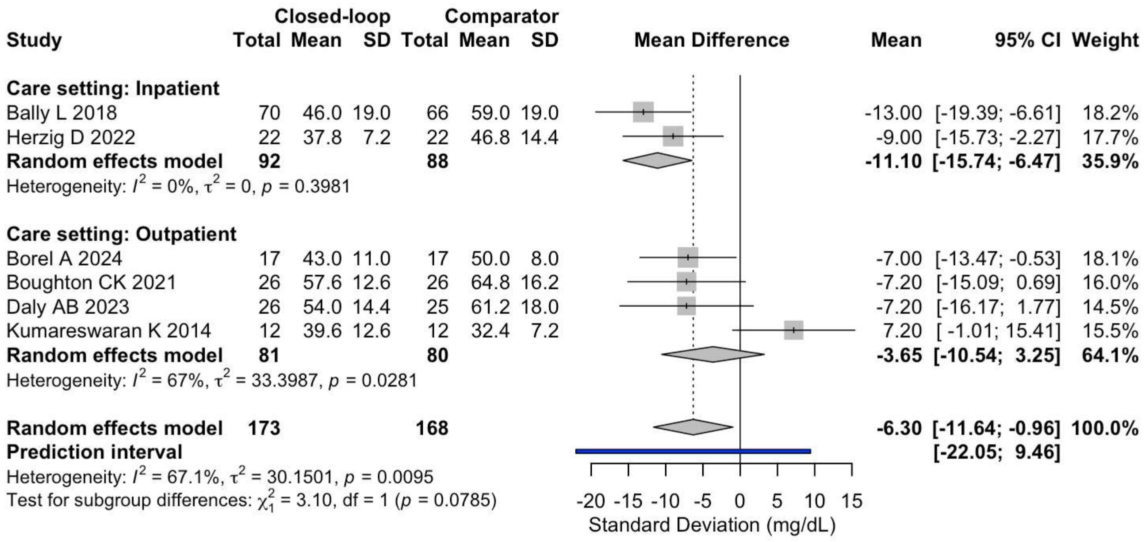
Closed-loop
Comparator
Study
Total Mean SD Total Mean SD
Mean Difference
Mean
95% CI Weight
Care setting: Inpatient
Bally L 2018 70 46.0 19.0 66 59.0 19.0 -13.00 [-19.39; -6.61] 18.2%
Herzig D 2022 22 37.8 7.2 22 46.8 14.4 -9.00 [-15.73; -2.27] 17.7%
Random effects model 92 88 -11.10 [-15.74; -6.47] 35.9%
Heterogeneity: I^2 = 0%, τ^2 = 0, p = 0.3981
Care setting: Outpatient
Borel A 2024 17 43.0 11.0 17 50.0 8.0 -7.00 [-13.47; -0.53] 18.1%
Boughton CK 2021 26 57.6 12.6 26 64.8 16.2 -7.20 [-15.09; 0.69] 16.0%
Daly AB 2023 26 54.0 14.4 25 61.2 18.0 -7.20 [-16.17; 1.77] 14.5%
Kumareswaran K 2014 12 39.6 12.6 12 32.4 7.2 7.20 [-1.01; 15.41] 15.5%
Random effects model 81 80 -3.65 [-10.54; 3.25] 64.1%
Heterogeneity: I^2 = 67%, τ^2 = 33.3987, p = 0.0281
Random effects model 173 168 -6.30 [-11.64; -0.96] 100.0%
Prediction interval [-22.05; 9.46]
Heterogeneity: I^2 = 67.1%, τ^2 = 30.1501, p = 0.0095
Test for subgroup differences: χ^2_1 = 3.10, df = 1 (p = 0.0785)
-20 -15 -10 -5 0 5 10 15
Standard Deviation (mg/dL)

CREDIBILITY ASSESSMENT - ICEMAN

Essential preliminary considerations to define the possible effect modification of interest

State a single candidate effect modifier (e.g., age or comorbidity): inpatient vs. outpatient care setting

Was the effect modifier measured before or at randomization? [x] yes, continue [ ] no, stop here and refer to manual for further instructions

State a single outcome and time-point (e.g., mortality at 1 year follow-up): CV (%)

State a single effect measure (e.g., relative risk or risk difference): mean difference

1: Is the analysis of effect modification based on comparison within rather than between trials?

[x] Completely between	[ ] Mostly between or unclear	[ ] Mostly within	[ ] Completely within
Subgroup analysis or meta-regression comparing overall effects of each individual trial. This is typical for aggregate data meta-analysis.	*Subgroup analysis or meta-regression with most information coming from overall effects, but some trials providing within-trial subgroup information*	*Most trials providing within-trial subgroup information; or individual participant data analysis that combines within and between trial information*	*All trials providing within-trial subgroup information or individual participant data; and the analysis separates within from between trial information, e.g., meta-analysis of interactions*

Comment:

2: For within-trial comparisons, is the effect modification similar from trial to trial? [ ] Not applicable: no or one within-RCT comparison

[ ] Definitely not similar	[ ] Probably not similar or unclear	[ ] Mostly similar	[ ] Definitely similar
Effect modification reported for two or more trials and clearly different directions	*Effect modification not reported for individual trials or too imprecise to tell*	*Effect modification reported for two or more trials, mostly similar in direction, but considerable differences in magnitude*	*Effect modification reported for two or more trials, similar in direction, only some differences in magnitude*

Comment:

3: For between-trial comparisons, is the number of trials large? [ ] Not applicable: no between RCT comparison

[x] Very small	[ ] Rather small or unclear	[ ] Rather large	[ ] Large
1 or 2 or in smallest subgroup; 5 or less in continuous meta-regression	*3-4 in smallest subgroup; 6-10 in continuous meta-regression*	*5-9 in smallest subgroup; 11 to 15 in continuous meta-regression*	*10 or more in smallest subgroup; more than 15 in continuous meta-regression*

Comment:

4: Was the direction of effect modification correctly hypothesized a priori?

[ ] Definitely no	[x] Probably no or unclear	[ ] Probably yes	[ ] Definitely yes
Clearly post-hoc or results inconsistent with hypothesized direction or biologically very implausible	*Vague hypothesis or hypothesized direction unclear*	*No prior protocol available but unequivocal statement of a priori hypothesis with correct direction of effect modification*	*Prior protocol available and includes correct specification of direction of effect modification, e.g., based on a biologic rationale*

Comment:

5: Does a test for interaction suggest that chance is an unlikely explanation of the apparent effect modification? (consider irrespective of number of effect modifiers)

[x] Chance a very likely explanation	[ ] Chance a likely explanation or unclear	[ ] Chance may not explain	[ ] Chance an unlikely explanation
Interaction or meta-regression p-value >0.05	*Interaction or meta-regression p-value ≤0.05 and >0.01, or no test of interaction reported and not computable*	*Interaction or meta-regression p-value ≤0.01 and >0.005*	*Interaction or meta-regression p-value ≤0.005*

Comment: p 0.07

6: Did the authors test only a small number of effect modifiers or consider the number in their statistical analysis?

[ ] Definitely no	[x] Probably no or unclear	[ ] Probably yes	[ ] Definitely yes
Explicitly exploratory analysis or large number of effect modifiers tested (e.g., greater than 10) and multiplicity not considered in analysis	*No mention of number or 4-10 effect modifiers tested and number not considered in analysis*	*No protocol available but unequivocal statement of 3 or fewer effect modifiers tested*	*Protocol available and 3 or fewer effect modifiers tested or number considered in analysis*

Comment:

7: Did the authors use a random effects model?

[ ] Definitely no	[ ] Probably no or unclear	[ ] Probably yes	[x] Definitely yes
Fixed (or common) effect or fixed effects model explicitly stated	*Probably fixed effect(s) model*	*Probably random (or mixed) effects*	*Random (or mixed) effects explicitly stated*

Comment:

8: If the effect modifier is a continuous variable, were arbitrary cut points avoided? [x] not applicable: not continuous

[ ] Definitely no	[ ] Probably no or unclear	[ ] Probably yes	[ ] Definitely yes
Analysis based on exploratory cut point(s), e.g., picking cut point associated with highest interaction p-value	*Analysis based on cut point(s) of unclear origin*	*Analysis based on pre-specified cut point(s), e.g., suggested by prior RCT*	*Analysis based on the full continuum, e.g., assuming a linear or logarithmic relationship*

Comment:

9 Optional: Are there any additional considerations that may increase or decrease credibility? (manual section 3.9) [x] not applicable

[ ] Yes, probably decrease [] Yes, probably increase

Comment:

10: How would you rate the overall credibility of the proposed effect modification?

The overall rating should be driven by the items that decrease credibility. The following provides a sensible strategy:

- All responses definitely or probably decrease credibility or unclear → very low
- Two or more responses definitely decrease credibility → maximum usually low even if all other responses satisfy credibility criteria
- One response definitely decreases credibility → maximum usually moderate even if all other responses satisfy credibility criteria
- Two responses probably decrease credibility → maximum usually moderate even if all other responses satisfy credibility criteria
- No response options definitely or probably decrease credibility → high very likely

Place a mark on the continuous line (or type “x” in editable version)

X

Very low credibility	Low credibility	Moderate credibility	High credibility
Minimal to no support for effect modification; Use overall effect for each subgroup	Some but insufficient support for effect modification; Use overall effect for each subgroup but note remaining uncertainty	Likely effect modification; Use separate effects for each subgroup but note remaining uncertainty	Very likely effect modification; Use separate effects for each subgroup

Comment: aggregate meta-analysis, very small number of studies in the smaller subgroup, chance probably an explanation, no a priori identification of the direction of the subgroup analysis, no convincing external evidence

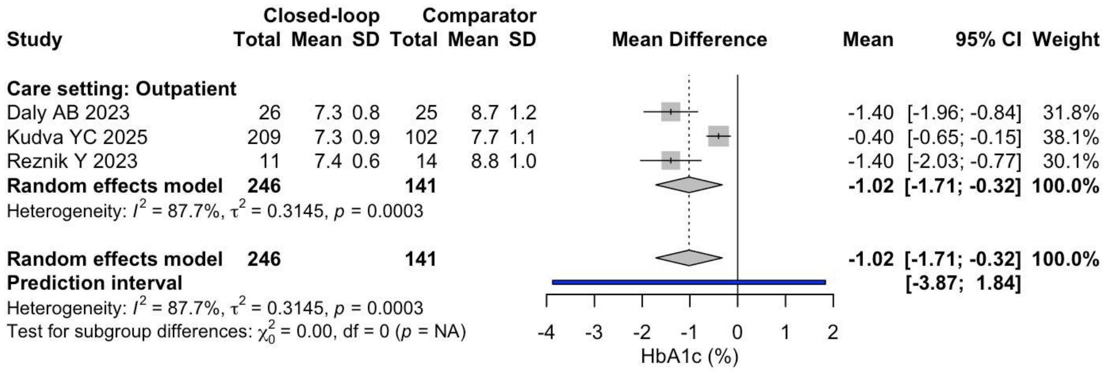

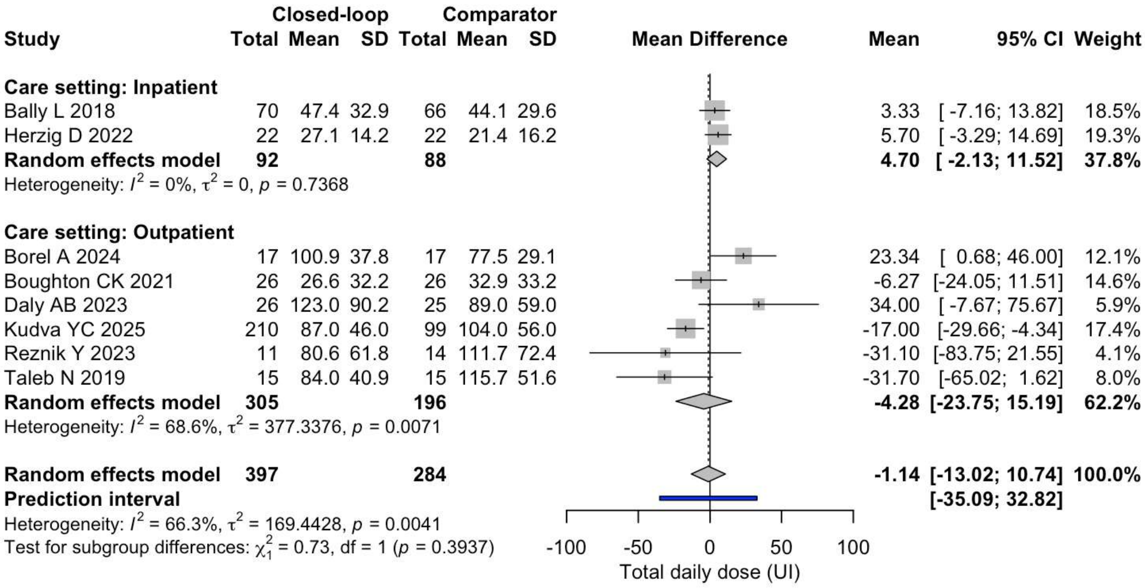

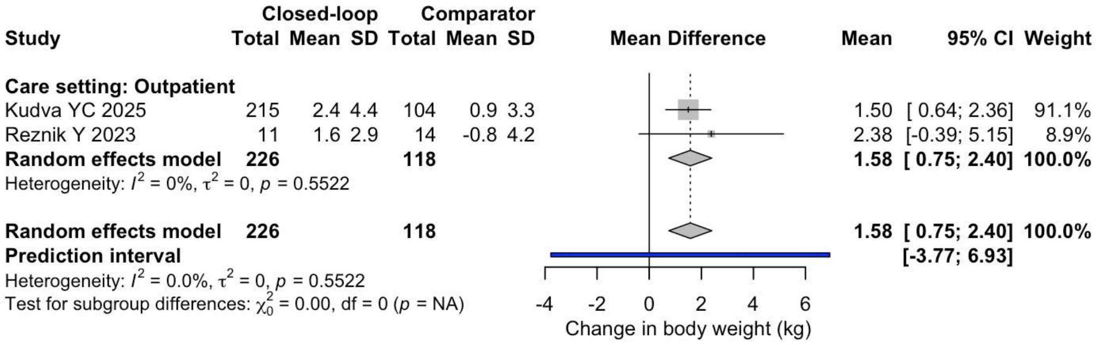

Each forest plot displays the pooled mean difference with corresponding 95% confidence intervals for each study, based on random-effects meta-analyses. The overall pooled estimate and its prediction interval are reported at the bottom of each plot. Measures of heterogeneity and subgroup differences are also provided when applicable. SD, standard deviation, CI, confidence interval.

Supplement 9. Meta-regression analysis

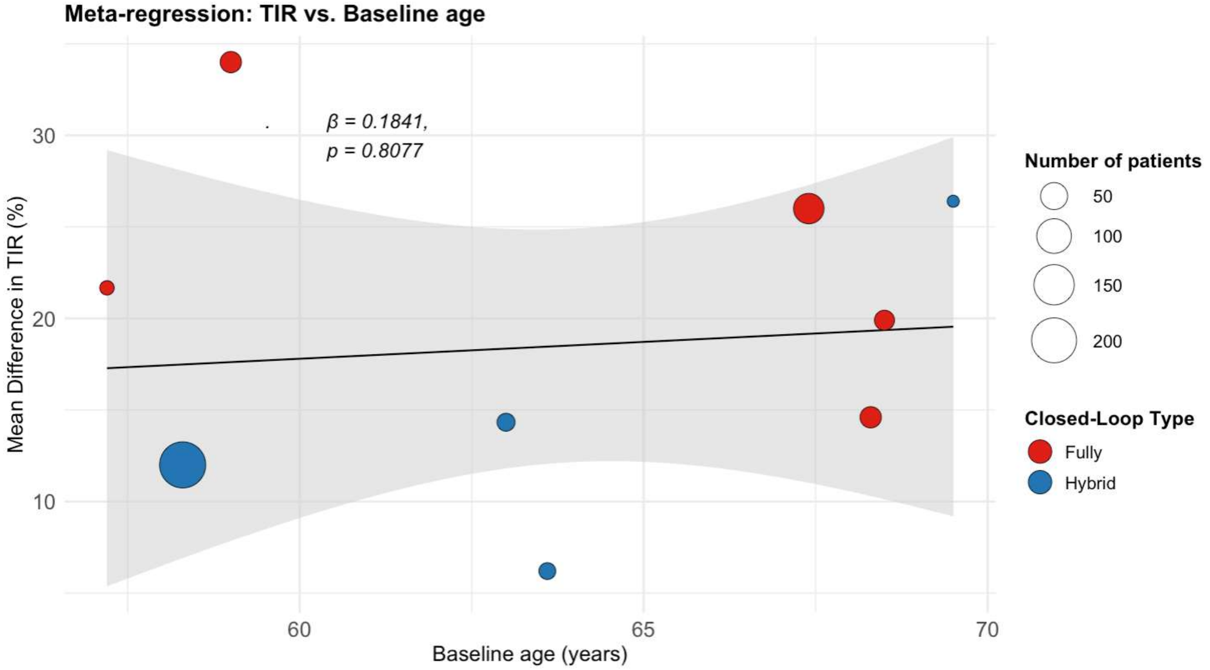

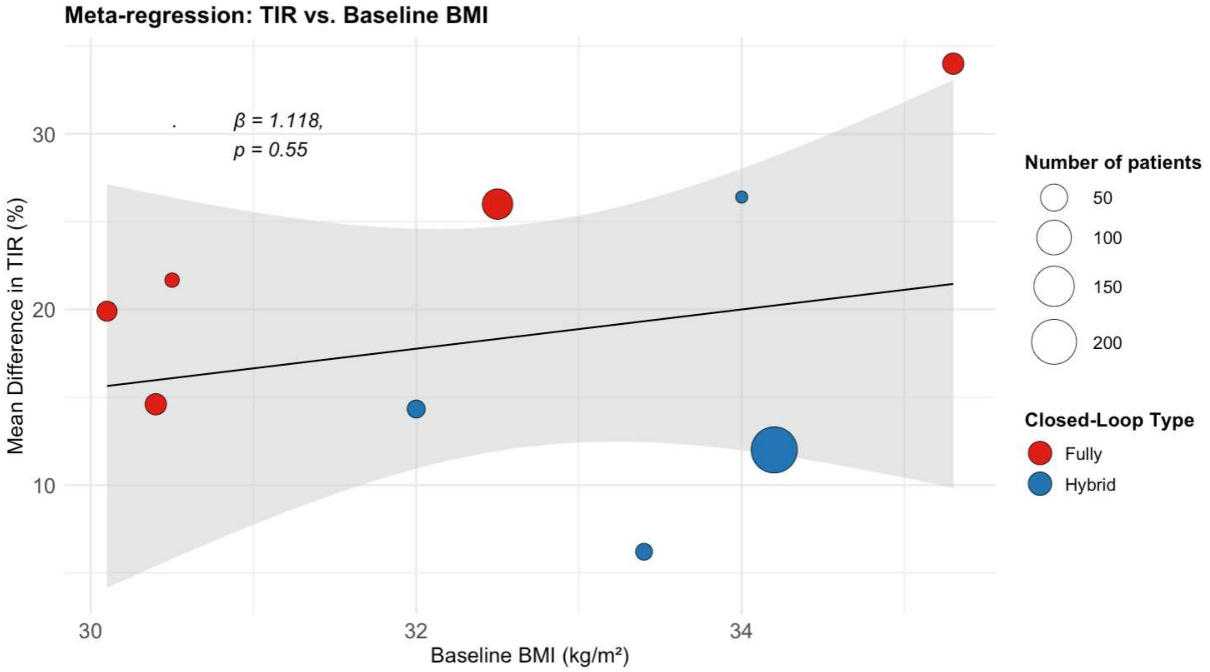

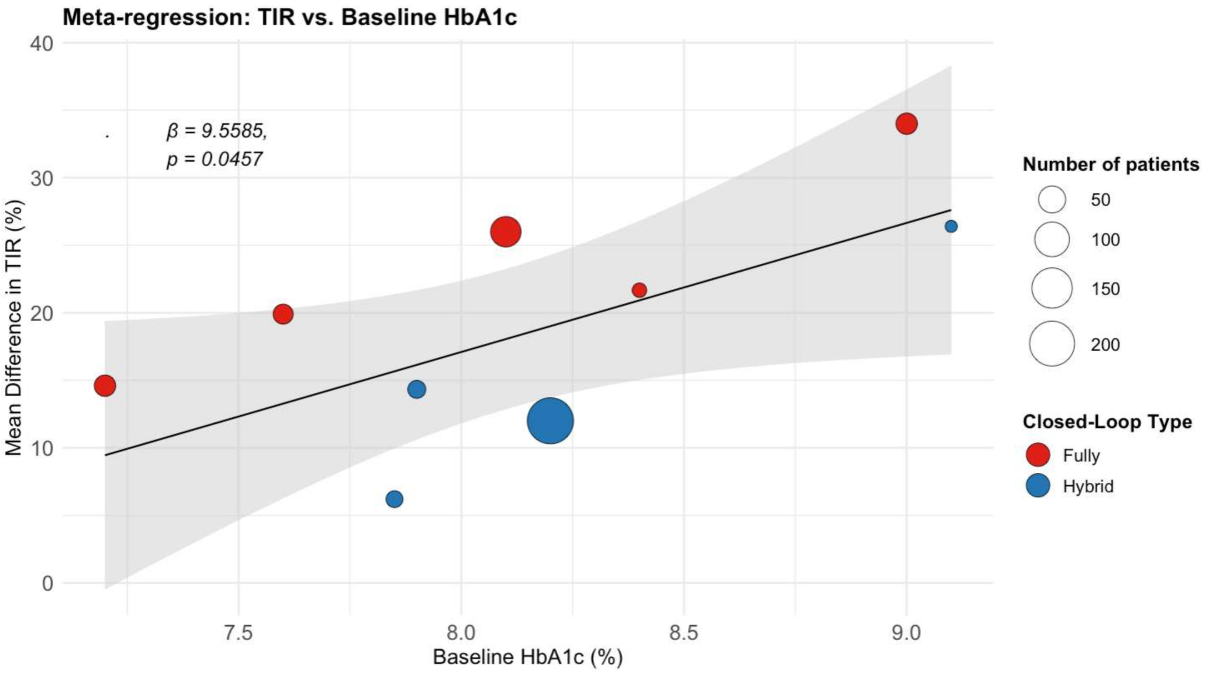

Meta-regression: TIR vs. Baseline HbA1c
β = 9.5585,
p = 0.0457
Mean Difference in TIR (%)
40
30
20
10
0
7.5
8.0
8.5
9.0
Baseline HbA1c (%)
Number of patients
50
100
150
200
Closed-Loop Type
Fully
Hybrid

CREDIBILITY ASSESSMENT - ICEMAN

Essential preliminary considerations to define the possible effect modification of interest

State a single candidate effect modifier (e.g., age or comorbidity): baseline HbA1c

Was the effect modifier measured before or at randomization? [x] yes, continue [ ] no, stop here and refer to manual for further instructions

State a single outcome and time-point (e.g., mortality at 1 year follow-up): TIR (%)

State a single effect measure (e.g., relative risk or risk difference): mean difference

1: Is the analysis of effect modification based on comparison within rather than between trials?

[x] Completely between	[ ] Mostly between or unclear	[ ] Mostly within	[ ] Completely within
Subgroup analysis or meta-regression comparing overall effects of each individual trial. This is typical for aggregate data meta-analysis.	*Subgroup analysis or meta-regression with most information coming from overall effects, but some trials providing within-trial subgroup information*	*Most trials providing within-trial subgroup information; or individual participant data analysis that combines within and between trial information*	*All trials providing within-trial subgroup information or individual participant data; and the analysis separates within from between trial information, e.g., meta-analysis of interactions*

Comment:

2: For within-trial comparisons, is the effect modification similar from trial to trial? [ ] Not applicable: no or one within-RCT comparison

[ ] Definitely not similar	[ ] Probably not similar or unclear	[ ] Mostly similar	[ ] Definitely similar
Effect modification reported for two or more trials and clearly different directions	*Effect modification not reported for individual trials or too imprecise to tell*	*Effect modification reported for two or more trials, mostly similar in direction, but considerable differences in magnitude*	*Effect modification reported for two or more trials, similar in direction, only some differences in magnitude*

Comment:

3: For between-trial comparisons, is the number of trials large? [ ] Not applicable: no between RCT comparison

[ ] Very small	[x] Rather small or unclear	[ ] Rather large	[ ] Large
1 or 2 or in smallest subgroup; 5 or less in continuous meta-regression	*3-4 in smallest subgroup; 6-10 in continuous meta-regression*	*5-9 in smallest subgroup; 11 to 15 in continuous meta-regression*	*10 or more in smallest subgroup; more than 15 in continuous meta-regression*

Comment:

4: Was the direction of effect modification correctly hypothesized a priori?

[ ] Definitely no	[x] Probably no or unclear	[ ] Probably yes	[ ] Definitely yes
Clearly post-hoc or results inconsistent with hypothesized direction or biologically very implausible	*Vague hypothesis or hypothesized direction unclear*	*No prior protocol available but unequivocal statement of a priori hypothesis with correct direction of effect modification*	*Prior protocol available and includes correct specification of direction of effect modification, e.g., based on a biologic rationale*

Comment:

5: Does a test for interaction suggest that chance is an unlikely explanation of the apparent effect modification? (consider irrespective of number of effect modifiers)

[ ] Chance a very likely explanation	[x] Chance a likely explanation or unclear	[ ] Chance may not explain	[ ] Chance an unlikely explanation
Interaction or meta-regression p-value >0.05	*Interaction or meta-regression p-value ≤0.05 and >0.01, or no test of interaction reported and not computable*	*Interaction or meta-regression p-value ≤0.01 and >0.005*	*Interaction or meta-regression p-value ≤0.005*

Comment: p 0.04

6: Did the authors test only a small number of effect modifiers or consider the number in their statistical analysis?

[ ] Definitely no	[x] Probably no or unclear	[ ] Probably yes	[ ] Definitely yes
Explicitly exploratory analysis or large number of effect modifiers tested (e.g., greater than 10) and multiplicity not considered in analysis	*No mention of number or 4-10 effect modifiers tested and number not considered in analysis*	*No protocol available but unequivocal statement of 3 or fewer effect modifiers tested*	*Protocol available and 3 or fewer effect modifiers tested or number considered in analysis*

Comment:

7: Did the authors use a random effects model?

[ ] Definitely no	[ ] Probably no or unclear	[ ] Probably yes	[x] Definitely yes
Fixed (or common) effect or fixed effects model explicitly stated	*Probably fixed effect(s) model*	*Probably random (or mixed) effects*	*Random (or mixed) effects explicitly stated*

Comment:

8: If the effect modifier is a continuous variable, were arbitrary cut points avoided? [x] not applicable: not continuous

[ ] Definitely no	[ ] Probably no or unclear	[ ] Probably yes	[ ] Definitely yes
Analysis based on exploratory cut point(s), e.g., picking cut point associated with highest interaction p-value	*Analysis based on cut point(s) of unclear origin*	*Analysis based on pre-specified cut point(s), e.g., suggested by prior RCT*	*Analysis based on the full continuum, e.g., assuming a linear or logarithmic relationship*

Comment:

9 Optional: Are there any additional considerations that may increase or decrease credibility? (manual section 3.9) [x] not applicable

[ ] Yes, probably decrease [] Yes, probably increase

Comment:

10: How would you rate the overall credibility of the proposed effect modification?

The overall rating should be driven by the items that decrease credibility. The following provides a sensible strategy:

- All responses definitely or probably decrease credibility or unclear → very low
- Two or more responses definitely decrease credibility → maximum usually low even if all other responses satisfy credibility criteria
- One response definitely decreases credibility → maximum usually moderate even if all other responses satisfy credibility criteria
- Two responses probably decrease credibility → maximum usually moderate even if all other responses satisfy credibility criteria
- No response options definitely or probably decrease credibility → high very likely

Place a mark on the continuous line (or type "x" in editable version)

X

**Very low credibility**	**Low credibility**	**Moderate credibility**	**High credibility**
Minimal to no support for effect modification;<br>Use overall effect for each subgroup	Some but insufficient support for effect modification;<br>Use overall effect for each subgroup but note remaining uncertainty	Likely effect modification;<br>Use separate effects for each subgroup but note remaining uncertainty	Very likely effect modification;<br>Use separate effects for each subgroup

Comment: aggregate meta-analysis, very small number of studies in the smaller subgroup, no a priori identification of the direction of the subgroup analysis, no convincing external evidence

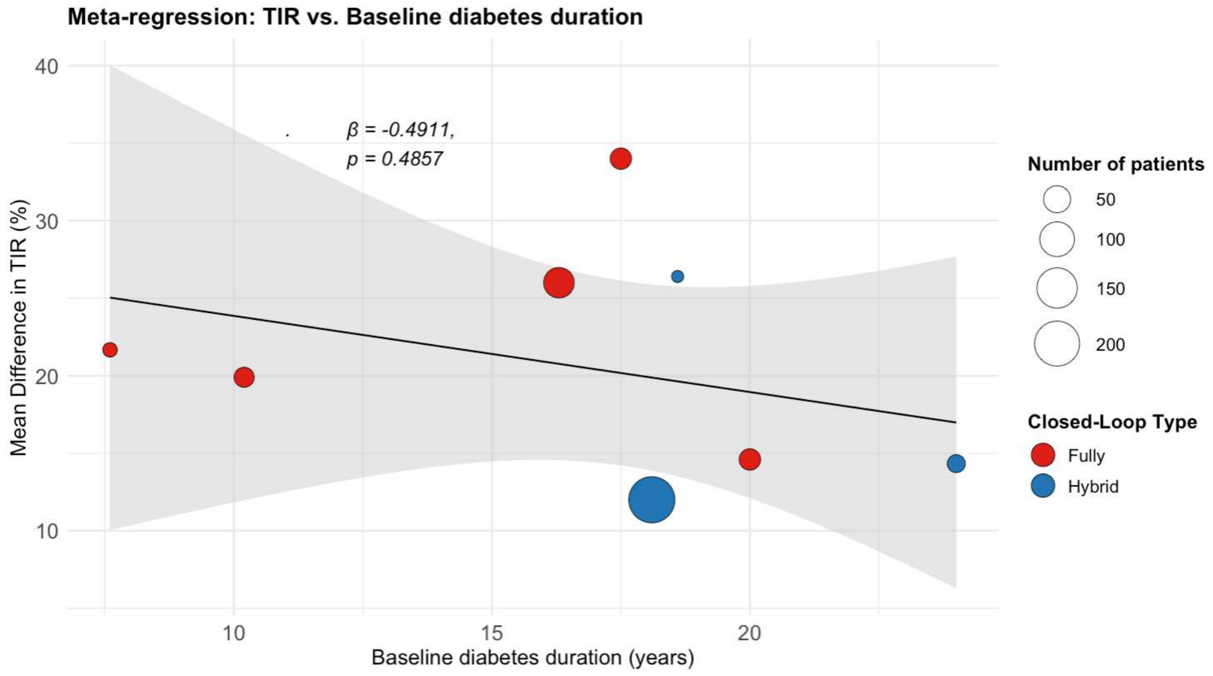
Meta-regression: TIR vs. Baseline diabetes duration
β = -0.4911,
p = 0.4857
Mean Difference in TIR (%)
40
30
20
10
10
15
20
Baseline diabetes duration (years)
Number of patients
50
100
150
200
Closed-Loop Type
Fully
Hybrid

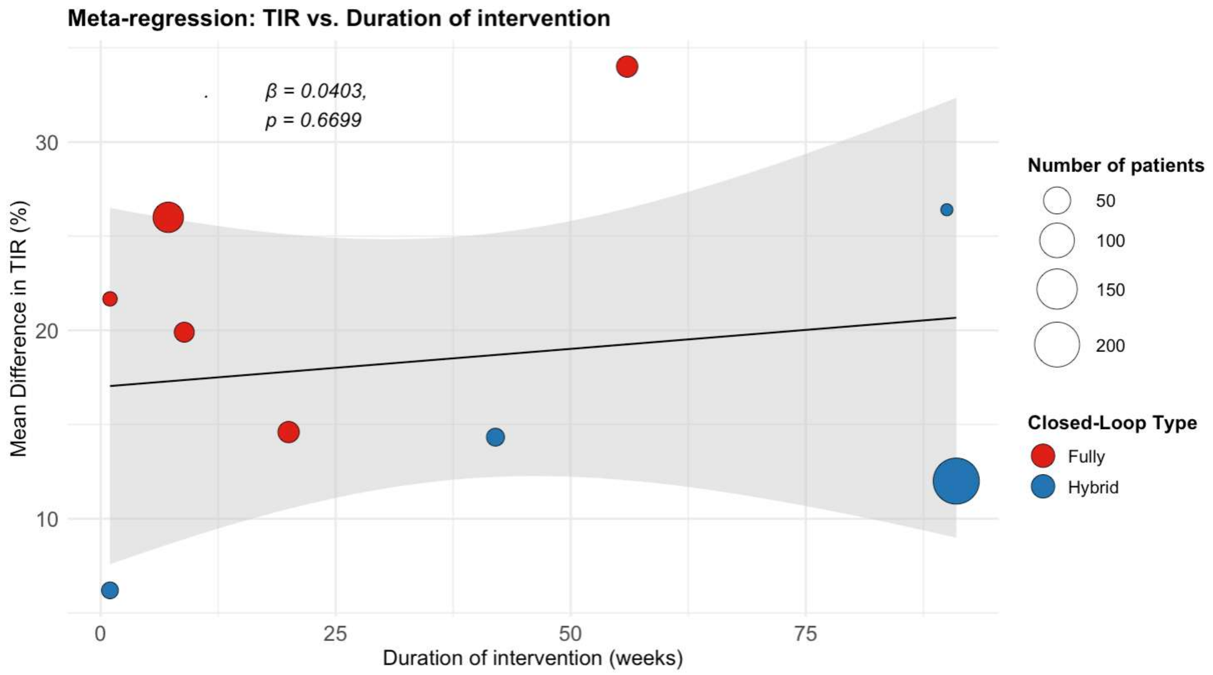
Meta-regression: TIR vs. Duration of intervention
β = 0.0403,
p = 0.6699
Mean Difference in TIR (%)
30
20
10
0
25
50
75
Duration of intervention (weeks)
Number of patients
50
100
150
200
Closed-Loop Type
Fully
Hybrid

Supplement 10. Severe adverse events

Author	Year	SH	DKA	All cause death	MACE	AMI	Stroke	Gangrene	Infected ulcer	Lower limb amputation	New/Prog retinopathy	New/Prog nephropathy
Kumareswaran K[1]	2014	0	0	NA	NA	NA	NA	NA	NA	NA	NA	NA
Bally L[2]	2018	0	0	no	no	no	no	no	no	no	no	no
Taleb N[3]	2019	NA	NA	no	no	no	no	no	no	no	no	no
Boughton CK[4]	2021	1 CL	0	1 *	no	no	1 control period	no	1 * §	1 control period	no	no
Herzig D[5]	2022	0	0	no	no	1 control	no	no	no	no	no	no
Daly AB[6]	2023	0	0	1 †	no	no	no	1 CL #	1 control	1 CL §	no	no
Reznik Y[7]	2023	0	0	no	no	no	no	no	no	no	no	no
Borel A[8]	2024	0	0	no	no	no	no	no	no	no	no	no
Kudva YC[9]	2025	1 CL	0	1 control	no	no	no	no	no	no	no	no

* washout or pre-study start; † pre-randomization; § not pre-specified event; # vascular intervention. SH, severe hypoglycemia; DKA, diabetic ketoacidosis; MACE, major adverse cardiovascular events; AMI, acute myocardial infarction; New, new incidence; Prog, worsening/progression; CL, closed loop; NA, not available.

Supplement 11. Patient-reported outcomes measures

Author	Year	Experience with CL system	EQ-5D-5L	DTSQs total	Perceived hyperglycemia	Perceived hypoglycemia	HCS	HFS-II	DIDS	PROMIS Sleep Impairment	PAID
Kumareswaran K[1]	2014	NA	NA	NA	NA	NA	NA	NA	NA	NA	NA
Bally L[2]	2018	Positive	NA	NA	NA	NA	NA	NA	NA	NA	NA
Taleb N[3]	2019	NA	NA	NA	NA	NA	NA	NA	NA	NA	NA
Boughton CK[4]	2021	Positive	NA	NA	NA	NA	Better	Equal	NA	NA	Equal
Herzig D[5]	2022	NA	NA	NA	NA	NA	NA	NA	NA	NA	NA
Daly AB[6]	2023	Positive	NA	NA	NA	NA	Equal	Worse	NA	NA	Equal
Reznik Y[7]	2023	Positive	Equal	NA	NA	NA	NA	Equal	NA	NA	NA
Borel A[8]	2024	Positive	NA	Equal	Better	Equal	NA	NA	NA	NA	NA
Kudva YC[9]	2025	NA	NA	NA	NA	NA	NA	NA	Equal *	Equal *	NA

* trend for better. CL, closed-loop; EQ-5D-5L, EuroQol 5 Dimensions 5 Levels; DTSQs, Diabetes Treatment Satisfaction Questionnaire - status version; HCS, Hypoglycemia Confidence Scale; HFS-II, Hypoglycemia Fear Survey, version II; DIDS, Diabetes Intrusiveness Scale; PROMIS Sleep Impairment, Patient-Reported Outcomes Measurement Information System – Sleep Impairment; PAID, Problem Areas in Diabetes.

Supplement 12. Prisma 2020 Checklist

PRISMA 2020 Main Checklist

Topic	No.	Item	Location where item is reported
**TITLE**			
**Title**	1	Identify the report as a systematic review.	1
**ABSTRACT**			
**Abstract**	2	See the PRISMA 2020 for Abstracts checklist	
**INTRODUCTION**			
**Rationale**	3	Describe the rationale for the review in the context of existing knowledge.	4
**Objectives**	4	Provide an explicit statement of the objective(s) or question(s) the review addresses.	4
**METHODS**			
**Eligibility criteria**	5	Specify the inclusion and exclusion criteria for the review and how studies were grouped for the syntheses.	5
**Information sources**	6	Specify all databases, registers, websites, organisations, reference lists and other sources searched or consulted to identify studies. Specify the date when each source was last searched or consulted.	5
**Search strategy**	7	Present the full search strategies for all databases, registers and websites, including any filters and limits used.	Supplementary
**Selection process**	8	Specify the methods used to decide whether a study met the inclusion criteria of the review, including how many reviewers screened each record and each report retrieved, whether they worked independently, and if applicable, details of automation tools used in the process.	6
**Data collection process**	9	Specify the methods used to collect data from reports, including how many reviewers collected data from each report, whether they worked independently, any processes for obtaining or confirming data from study investigators, and if applicable, details of automation tools used in the process.	6
**Data items**	10a	List and define all outcomes for which data were sought. Specify whether all results that were compatible with each outcome domain in each study were sought (e.g. for all measures, time points, analyses), and if not, the methods used to decide which results to collect.	6
	10b	List and define all other variables for which data were sought (e.g. participant and intervention characteristics, funding sources). Describe any assumptions made about any missing or unclear information.	6
**Study risk of bias assessment**	11	Specify the methods used to assess risk of bias in the included studies, including details of the tool(s) used, how many reviewers assessed each study and whether they worked independently, and if applicable, details of automation tools used in the process.	6
**Effect measures**	12	Specify for each outcome the effect measure(s) (e.g. risk ratio, mean difference) used in the synthesis or presentation of results.	6
**Synthesis methods**	13a	Describe the processes used to decide which studies were eligible for each synthesis (e.g. tabulating the study intervention characteristics and comparing against the planned groups for each synthesis (item 5)).	7
	13b	Describe any methods required to prepare the data for presentation or synthesis, such as handling of missing summary statistics, or data conversions.	7

	13c	Describe any methods used to tabulate or visually display results of individual studies and syntheses.	Supplementary
	13d	Describe any methods used to synthesize results and provide a rationale for the choice(s). If meta-analysis was performed, describe the model(s), method(s) to identify the presence and extent of statistical heterogeneity, and software package(s) used.	7
	13e	Describe any methods used to explore possible causes of heterogeneity among study results (e.g. subgroup analysis, meta-regression).	7
	13f	Describe any sensitivity analyses conducted to assess robustness of the synthesized results.	7
**Reporting bias assessment**	14	Describe any methods used to assess risk of bias due to missing results in a synthesis (arising from reporting biases).	6
**Certainty assessment**	15	Describe any methods used to assess certainty (or confidence) in the body of evidence for an outcome.	7
**RESULTS**			
**Study selection**	16a	Describe the results of the search and selection process, from the number of records identified in the search to the number of studies included in the review, ideally using a flow diagram.	7-8
	16b	Cite studies that might appear to meet the inclusion criteria, but which were excluded, and explain why they were excluded.	Supplementary
**Study characteristics**	17	Cite each included study and present its characteristics.	Supplementary
**Risk of bias in studies**	18	Present assessments of risk of bias for each included study.	-
**Results of individual studies**	19	For all outcomes, present, for each study: (a) summary statistics for each group (where appropriate) and (b) an effect estimate and its precision (e.g. confidence/credible interval), ideally using structured tables or plots.	8-9, Supplementary
**Results of syntheses**	20a	For each synthesis, briefly summarise the characteristics and risk of bias among contributing studies.	8-10, Supplementary
	20b	Present results of all statistical syntheses conducted. If meta-analysis was done, present for each the summary estimate and its precision (e.g. confidence/credible interval) and measures of statistical heterogeneity. If comparing groups, describe the direction of the effect.	8-10, Supplementary
	20c	Present results of all investigations of possible causes of heterogeneity among study results.	8-10, Supplementary
	20d	Present results of all sensitivity analyses conducted to assess the robustness of the synthesized results.	8-10, Supplementary
**Reporting biases**	21	Present assessments of risk of bias due to missing results (arising from reporting biases) for each synthesis assessed.	8-10, Supplementary
**Certainty of evidence**	22	Present assessments of certainty (or confidence) in the body of evidence for each outcome assessed.	8-10, Supplementary
**DISCUSSION**			
**Discussion**	23a	Provide a general interpretation of the results in the context of other evidence.	10-13
	23b	Discuss any limitations of the evidence included in the review.	12-13
	23c	Discuss any limitations of the review processes used.	13
	23d	Discuss implications of the results for practice, policy, and future research.	13-14
**OTHER INFORMATION**			

**Registration and protocol**	24a	Provide registration information for the review, including register name and registration number, or state that the review was not registered.	5
	24b	Indicate where the review protocol can be accessed, or state that a protocol was not prepared.	https://www.crd.york.ac.uk/PROSPERO/view/CRD420251083874
	24c	Describe and explain any amendments to information provided at registration or in the protocol.	5
**Support**	25	Describe sources of financial or non-financial support for the review, and the role of the funders or sponsors in the review.	15
**Competing interests**	26	Declare any competing interests of review authors.	14-15
**Availability of data, code and other materials**	27	Report which of the following are publicly available and where they can be found: template data collection forms; data extracted from included studies; data used for all analyses; analytic code; any other materials used in the review.	15

PRIMSA Abstract Checklist

Topic	No.	Item	Reported?
**TITLE**			
**Title**	1	Identify the report as a systematic review.	Yes
**BACKGROUND**			
**Objectives**	2	Provide an explicit statement of the main objective(s) or question(s) the review addresses.	Yes
**METHODS**			
**Eligibility criteria**	3	Specify the inclusion and exclusion criteria for the review.	Yes
**Information sources**	4	Specify the information sources (e.g. databases, registers) used to identify studies and the date when each was last searched.	Yes
**Risk of bias**	5	Specify the methods used to assess risk of bias in the included studies.	Yes
**Synthesis of results**	6	Specify the methods used to present and synthesize results.	Yes
**RESULTS**			
**Included studies**	7	Give the total number of included studies and participants and summarise relevant characteristics of studies.	Yes
**Synthesis of results**	8	Present results for main outcomes, preferably indicating the number of included studies and participants for each. If meta-analysis was done, report the summary estimate and confidence/credible interval. If comparing groups, indicate the direction of the effect (i.e. which group is favoured).	Yes
**DISCUSSION**			
**Limitations of evidence**	9	Provide a brief summary of the limitations of the evidence included in the review (e.g. study risk of bias, inconsistency and imprecision).	Yes
**Interpretation**	10	Provide a general interpretation of the results and important implications.	Yes
**OTHER**			
**Funding**	11	Specify the primary source of funding for the review.	Yes
**Registration**	12	Provide the register name and registration number.	Yes

From: Page MJ, McKenzie JE, Bossuyt PM, Boutron I, Hoffmann TC, Mulrow CD, et al. The PRISMA 2020 statement: an updated guideline for reporting systematic reviews. MetaArXiv. 2020, September 14. DOI: 10.31222/osf.io/v7gm2. For more information, visit: www.prisma-statement.org

References

1. Kumareswaran K, Thabit H, Leelarathna L, et al. Feasibility of closed-loop insulin delivery in type 2 diabetes: a randomized controlled study. *Diabetes Care*. 2014;37(5):1198-1203.

2. Bally L, Thabit H, Hartnell S, et al. Closed-loop insulin delivery for glycemic control in noncritical care. *N Engl J Med*. 2018;379(6):547-556.

3. Taleb N, Carpentier AC, Messier V, Ladouceur M, Haidar A, Rabasa-Lhoret R. Efficacy of artificial pancreas use in patients with type 2 diabetes using intensive insulin therapy: A randomized crossover pilot trial. *Diabetes Care*. 2019;42(7):e107-e109.

4. Boughton CK, Tripyla A, Hartnell S, et al. Fully automated closed-loop glucose control compared with standard insulin therapy in adults with type 2 diabetes requiring dialysis: an open-label, randomized crossover trial. *Nat Med*. 2021;27(8):1471-1476.

5. Herzig D, Suhner S, Roos J, et al. Perioperative fully closed-loop insulin delivery in patients undergoing elective surgery: An open-label, randomized controlled trial. *Diabetes Care*. 2022;45(9):2076-2083.

6. Daly AB, Boughton CK, Nwokolo M, et al. Fully automated closed-loop insulin delivery in adults with type 2 diabetes: an open-label, single-center, randomized crossover trial. *Nat Med*. 2023;29(1):203-208.

7. Reznik Y, Carvalho M, Fendri S, et al. Should people with type 2 diabetes treated by multiple daily insulin injections with home health care support be switched to hybrid closed-loop? The CLOSE AP+ randomized controlled trial. *Diabetes Obes Metab*. 2024;26(2):622-630.

8. Borel AL, Lablanche S, Waterlot C, et al. Closed-loop insulin therapy for people with type 2 diabetes treated with an insulin pump: A 12-week multicenter, open-label randomized, controlled, crossover trial. *Diabetes Care*. 2024;47(10):1778-1786.

9. Kudva YC, Raghinaru D, Lum JW, et al. A randomized trial of automated insulin delivery in type 2 diabetes. *N Engl J Med*. 2025;392(18):1801-1812.
